# Deciphering the genetic basis of wheat germination under ZnO nano priming and drought stress through integrated QTL mapping and network analyses

**DOI:** 10.1186/s12870-026-08898-9

**Published:** 2026-05-11

**Authors:** Mennatalla R. I. Mahmoud, Ahmed Sallam, Mohamed A. Karam, Yasser S. Moursi

**Affiliations:** 1 Department of Botany, Faculty of Science, Fayoum University, Fayoum, 63514 Egypt; 2 School of Biotechnology, Badr University in Assiut (BUA), Assiut, Egypt; 3 Department of Genetics, Faculty of Agriculture, Assiut University, Assiut, 71526 Egypt

**Keywords:** Germination and seedling traits, Drought‑responsive genes, Quantitative trait loci (QTLs), Wheat (*Triticum aestivum L*.), ZnO nanoparticle priming, Network analysis

## Abstract

**Background:**

Wheat (*Triticum aestivum L.*) is one of the most important staple crops worldwide. However, its productivity, grain quality, and nutritional value are increasingly threatened by climate change, particularly drought stress. Rapid global population growth, coupled with these climatic challenges, is expected to intensify malnutrition and food insecurity, thereby increasing the risk of famine in vulnerable regions. Nanotechnology‑based approaches, especially zinc oxide nanoparticle (ZnO-NPs) seed nano priming, have emerged as promising strategies to enhance seed germination, early seedling growth, and drought tolerance in wheat.

**Results:**

This study phenotypically evaluated 113 doubled haploid (DH) wheat genotypes, along with two parental lines, for 22 germination‑ and seedling‑related traits under optimal (C; 0% PEG 6000) and drought stress (D; 18% PEG 6000) conditions, with and without ZnO nanoparticles (ZnO-NPs) priming. Subsequently, to identify genomic regions and candidate genes associated with drought‑responsive phenotypes, quantitative trait loci (QTL) mapping was performed using Inclusive Composite Interval Mapping (ICIM‑ADD) on a subset of 98 DH genotypes, following exclusion of 15 lines due to low quality marker data. All measured traits were significantly affected by drought stress; however, under both control and drought conditions, ZnO-NP nano priming consistently improved seed germination and seedling establishment. Overall, 51 QTLs associated with 22 traits were detected across the four treatments, and 86 candidate genes were located within the confidence intervals of the identified QTLs. Functional annotation indicated that these genes encode metal-binding proteins, transcription factors, ion transporters, enzymes, and zinc‑binding proteins involved in stress tolerance. Notably, both primed and unprimed conditions revealed stable and reliable QTLs associated with drought tolerance. Functional connectivity, key hub genes, and protein clusters were further revealed through network analysis.

**Conclusion:**

More effective wheat breeding programs can be achieved through the integration of molecular breeding approaches with nanotechnology. Furthermore, the incorporation of gene–gene, gene–protein, and protein–protein interaction network analyses provide deeper insights into the regulatory modules and functional connectivity underlying drought tolerance. These network‑based approaches facilitate the identification of key hub genes and protein clusters that coordinate stress signaling and metabolic pathways, thereby offering powerful and promising targets for molecular breeding and crop improvement strategies.

**Supplementary Information:**

The online version contains supplementary material available at 10.1186/s12870-026-08898-9.

## Background

Climate change poses substantial challenges to agriculture, water resources, and global food security [1, 2]. Wheat (*Triticum aestivum L*.) is the most widely cultivated cereal crop worldwide and plays a pivotal role in sustaining global food security [3]. However, wheat productivity is increasingly threatened by the rising frequency and severity of drought events driven by climate warming, thereby jeopardizing food security, economic stability, and crop production systems [4]. The magnitude of drought‑induced damage in wheat is influenced by multiple factors, including stress intensity and duration, the developmental stage at which drought occurs, and the genetic constitution of the cultivated genotype [5–7].

Wheat is the primary staple food for approximately 2.5 billion people worldwide and represents a major source of carbohydrates, proteins, and essential micronutrients, particularly iron (Fe) and zinc (Zn) [8, 9]. Beyond its direct contribution to human nutrition, wheat also provides straw, which serves as an important feed resource for livestock [10, 11]. The crop contributes substantially to total agricultural output and imports, underscoring its pivotal role in national economies and food security systems. In many countries, wheat accounts for nearly 20% of total agricultural imports and approximately 10% of the overall value of agricultural production [12]. Globally, wheat remains one of the most important cereal crops, supporting both domestic food supplies and international trade and thereby playing a central role in global food security [13].

By 2050, the global population is projected to reach approximately 9.7 billion, substantially increasing the demand for food and adequate nutrition [14]. The rising global population has already led to increased demand for wheat, underscoring the vital role of wheat in ensuring food security [13, 14]. Accelerating gains in wheat yield productivity is therefore essential to meet projected food requirements by 2030, highlighting the urgent need to intensify efforts in wheat production and breeding programs [15]. However, achieving this goal is increasingly constrained by climate change and the ongoing loss of arable land. Despite rapid population growth in recent decades, the expansion of cultivated land has remained limited, increasing by only about 80 million hectares (~ 5%) between 2001 and 2023, with opportunities for further large‑scale expansion becoming progressively restricted [16]. Under these conditions, enhancing wheat resilience to environmental stresses, particularly drought, represents a sustainable and effective strategy for maintaining stable yields in the face of increasing climatic challenges [17, 18].

Innovative approaches are increasingly being adopted in modern agriculture to address climate‑related challenges, particularly drought stress. Among these, nanotechnology offers promising strategies using nanoparticles (1–100 nm) with unique physicochemical properties that can enhance plant growth, productivity, and stress tolerance [19]. However, the interaction between nanoparticles and crops is complex, as their effects can range from growth stimulation to potential phytotoxicity depending on the concentration, particle size, and plant species [20]. Seed nano‑priming, although a relatively recent technique, has shown considerable potential in improving germination performance and overall plant growth under adverse environmental conditions [21]. Seed priming alleviates drought stress by enhancing the mobilization of seed reserves and activating metabolic processes prior to germination, thereby promoting faster, more uniform, and robust seedling establishment [22]. Although nano‑priming has not yet been extensively studied in wheat, emerging evidence suggests its effectiveness in improving germination and early growth under water‑limited conditions. Among essential micronutrients, zinc (Zn) plays a crucial role in plant growth, yield formation, and human nutrition, functioning as a co‑factor for hundreds of enzymes and increasingly recognized as a limiting nutrient in many cropping systems [23, 24]. Zinc oxide nanoparticles (ZnO-NPs) have attracted considerable attention due to their unique physicochemical properties and their ability to enhance zinc uptake, seedling growth, and crop yield [25, 26]. ZnO nano‑priming has also been shown to modulate stress‑ and defense‑related gene expression, thereby strengthening plant resilience and potentially reducing the dependence on chemical pesticide applications [27]. In this context, the evaluation of wheat genetic resources is crucial for future crop improvement under changing environmental conditions [28, 29]. Substantial gains in wheat productivity can be achieved by integrating pre‑breeding materials and elite commercial cultivars within genomics‑driven breeding strategies [30]. As climate change intensifies, the development of drought‑tolerant wheat varieties has become increasingly imperative. The conservation and effective utilization of wheat’s broad genetic diversity are fundamental to ensuring long‑term productivity and global food security. Extensive genetic variation in wheat underpins drought adaptation through its effects on root system architecture, water‑use efficiency, and the regulation of drought‑responsive metabolic pathways [31]. This genetic diversity facilitates the development of cultivars with enhanced drought resilience and stable productivity under water‑limited conditions, enabling breeders to produce durable, high‑performing wheat varieties capable of sustaining yield and quality in increasingly variable climates [32].

Drought stress represents one of the most severe constraints to wheat productivity worldwide, making the accelerated development of drought‑tolerant wheat varieties imperative [33]. However, the genetic mechanisms underlying drought tolerance are highly complex, posing a major challenge to the development of resilient cultivars. Advances in molecular breeding strategies, including genomic selection, genome‑wide association studies (GWAS), and quantitative trait loci (QTL) mapping, have provided powerful tools for improving complex quantitative traits related to drought adaptation [34]. The establishment of high‑resolution linkage maps across crop species has further enabled the dissection of the genetic architecture governing drought responses. Numerous QTLs associated with germination and seedling traits, such as germination percentage, mean germination time, seedling vigor, shoot and root length, and fresh and dry biomass, have been identified in wheat under drought‑stress conditions [35]. These findings have substantially advanced our understanding of the genetic basis underlying germination and early seedling responses to water deficit in wheat [34]. Moreover, the application of modern gene‑editing technologies, such as CRISPR‑Cas9, to manipulate major QTLs presents a promising strategy for the development of drought‑tolerant transgenic wheat cultivars [36]. As climate change intensifies, breeding drought‑resilient wheat hybrids has become a critical objective for sustainable crop improvement. In this regard, network‑based approaches, including gene–gene, gene–protein, and protein–protein interaction analyses, are increasingly recognized as effective tools for unraveling the complex regulatory architecture of drought tolerance. These integrative frameworks facilitate the identification of key hub genes and protein networks that coordinate stress signaling and metabolic pathways, thereby providing valuable targets for molecular breeding. For instance, recent genome-wide association studies coupled with gene network analysis have successfully identified core regulatory modules governing drought resilience during wheat’s early growth stages, pinpointing specific hub genes that synchronize physiological and molecular responses [37]. Recent studies and reviews highlight that integrating QTL mapping with network biology enhances the discovery of functional regulatory modules and accelerates the development of resilient cultivars under water‑deficit conditions [38–40].

As yield is the most important target in wheat breeding programs, relatively few studies have focused on quantitative trait loci (QTLs) associated with drought stress during the germination and seedling stages. Although numerous QTLs have been reported for yield‑related traits and agronomic performance under water‑deficit conditions, the early developmental phases, particularly germination and seedling establishment, have received comparatively limited attention, despite their critical importance for crop stand establishment and subsequent productivity. Previous studies have demonstrated that drought‑responsive QTLs at the germination stage are commonly associated with traits such as germination percentage, root length, and seedling vigor, highlighting the genetic complexity of early‑stage drought tolerance and indicating that multiple loci contribute to variation in seedling establishment under water‑limited conditions [34, 39, 40]. Addressing this knowledge gap is essential for improving our understanding of the genetic basis of drought resilience during the earliest growth phases. Furthermore, genetic validation plays a pivotal role in ensuring that discovered genomic regions are robust enough for practical application in breeding, acting as a bridge between basic research and crop improvement [41]. Therefore, identifying and validating stable QTLs at these early stages is crucial for developing cultivars with enhanced field establishment under water-limited conditions.

Accordingly, the objectives of this study were to: (i) investigate the effects of drought stress on seed germination and seedling development in a wheat doubled haploid (DH) population; (ii) evaluate the impact of zinc oxide nanoparticle (ZnO-NPs) priming on germination performance under water‑deficit conditions; (iii) identify genomic regions (QTLs) and candidate genes associated with germination and seedling traits in primed and unprimed wheat DH populations under both control and drought conditions; and (iv) explore gene–gene, gene–protein, and protein–protein interaction networks associated with drought tolerance.

## Materials and methods

**Fig. 1:**
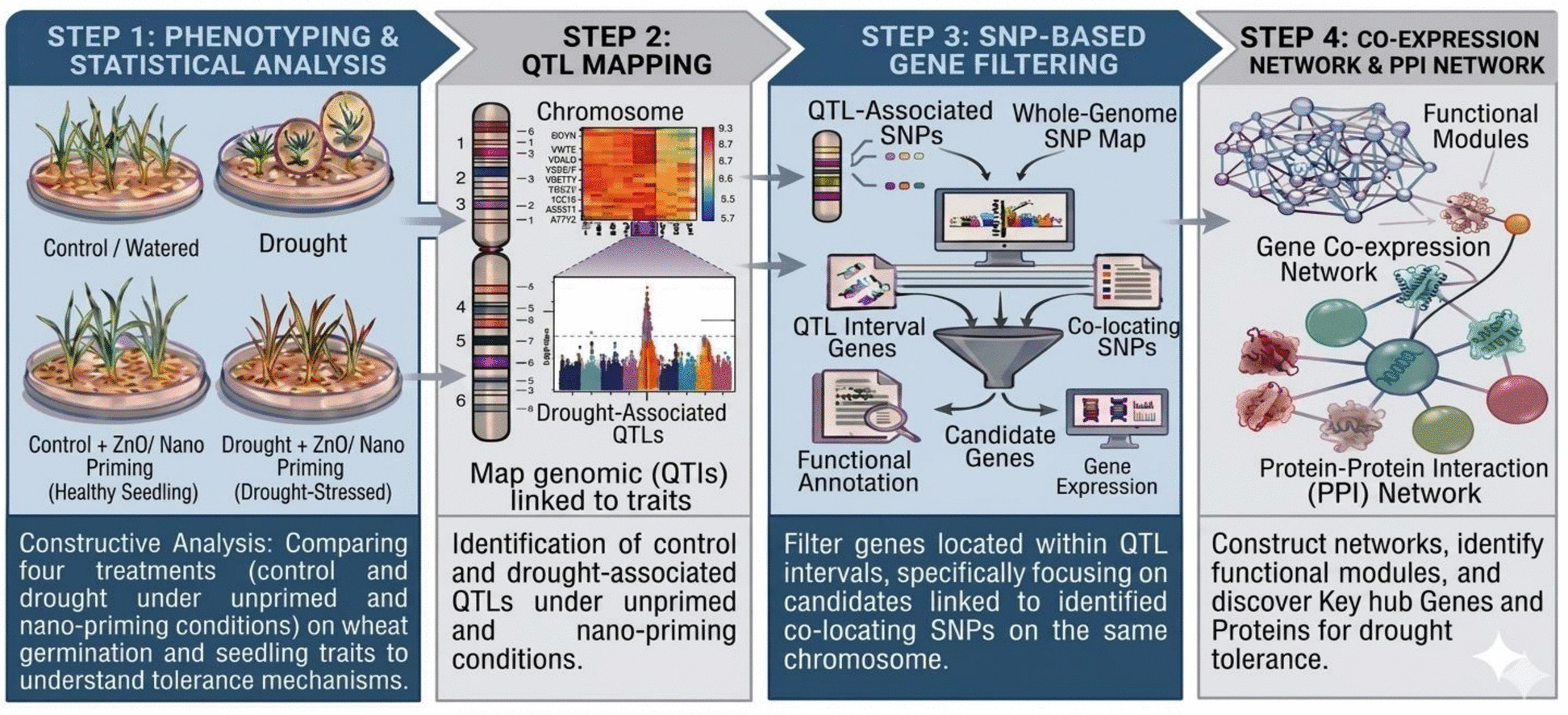
Schematic representation of the integrated research workflow employed in this study. The workflow outlines the sequential analytical steps, beginning with phenotyping and statistical analyses of germination and seedling traits under contrasting water and ZnO-nano priming conditions, followed by QTL mapping to identify drought-associated genomic regions. Candidate genes were subsequently filtered based on QTL intervals, functional annotation, and gene-expression profiles. Co-expression and protein–protein interaction networks were then constructed to identify functional modules, culminating in the identification of key hub genes and proteins associated with drought tolerance

To integrate and synthesize the multi-faceted approach of this study, a comprehensive schematic flowchart outlining the sequential steps from phenotyping to hub-gene identification is presented Fig. 1. Schematic flowchart illustrates the comprehensive research workflow. The sequential steps encompass phenotyping and statistical analyses, QTL mapping to identify the genomic regions, candidate-gene filtering based on integration of annotation, gene expression analysis, co-expression network construction, and final identification of key hub genes and proteins.

### Plant material

The plant material used in this study consisted of a wheat doubled haploid (DH) population derived from two parental lines that were selected from a genetically diverse panel representing different geographical origins. The spring wheat double haploid (DH) population was developed by crossing the accessions TRI-10703 (parent-A) x TRI-5310 (parent-B) [42]. The parental lines exhibited contrasting responses to drought stress, with TRI-10703 identified as (drought-tolerant), whereas TRI-5310 was characterized as (drought-sensitive). The seeds of the accessions used in the current study, were taken from the gene bank at Leibniz Institute of Plant Genetics and Crop Plant Research (IPK) Gatersleben, Germany. The DH population and its parents were genotyped with the wheat 15 k Infinium single nucleotide polymorphism (SNP) array developed at TraitGenetics GmbH http://www.traitgenetics.com/en/.

### Experimental layout

In the current study, a total of 113 double haploid genotypes together with their two parental lines were phenotypically evaluated in a laboratory germination experiment under drought stress conditions, with and without ZnO nano‑priming (ZnO-NPs). Drought experiments were conducted in the Plant Genetics Lab, Faculty of Science, Fayoum University, following the experimental design previously established by Mahmoud et al. [43].

#### Optimization of PEG 6000 and ZnO-NPs treatments

The selection of the optimal stress levels and priming conditions was based on preliminary experiments and our prior optimization protocols [43].

Drought Induction: To identify the discriminative concentration of Polyethylene glycol 6000 (PEG 6000), a series of solutions (0%, 10%, 15%, 18%, and 20% W/V) was tested. While 10% and 15% caused mild effects and 20% proved lethal, the 18% PEG 6000 solution was selected as it effectively differentiated drought tolerance among the genotypes.

Nano-priming Optimization: The objective was to determine the optimal ZnO nano priming concentration and duration that maintain high germination rates while maximizing the phenotypic variation between control and drought stress treatments, as well as among genotypes under drought stress. The ZnO-NPs used for nano-priming were purchased from Sigma Aldrich (St. Louis, MO, USA). According to the manufacturer’s specification, these ZnO-NPs are high-purity ZnO nanoparticles with a nominal primary particle size of 20–30 nm, indicating a nanoscale particle-size distribution within this range. The ZnO-NPs (Sigma-Aldrich, 20–30 nm) were freshly prepared by dispersing particles in deionized water using ultrasonic vibration (100 W, 40 kHz) for 30 min to ensure a stable suspension and minimize aggregation. Based on our previous screening of concentrations (50 ppm, 100 ppm, and 150 ppm) and priming durations (6 h, 12 h, and 18 h), the 100 ppm ZnO-NPs for 6 h was chosen, as described by Mahmoud et al. [43]. This combination was the most discriminative, as longer durations (12 h – 18 h) led to premature germination during the priming process.

#### Main experiment

All genotypes were evaluated under control (0% PEG 6000) and drought stress (18% PEG 6000) during seed germination and seedling establishment with and without ZnO-NPs nano priming in a randomized complete block design (RCBD) with three replications. Twenty grains from each genotype were washed with water and sterilized in 1% sodium hypochloride (NaOCl) for 10 min, then washed several times with deionized distilled water. The grains were primed by soaking for 6 h at room temperature at 25 °C in (100 ppm – ZnO-NPs) and kept on shaking at low speed during priming. Drought stress treatments were induced using 18% W/V of PEG 6000, while 0% PEG 6000 was used as a control (unstressed grains). From each genotype, 20 grains were placed in a 9 cm petri dish on two layers of filter papers under four treatments: unprimed conditions, T1 = control (10 mL of distilled water) and T2 = drought stress (10 mL of 18% PEG 6000) and for ZnO-NPs-primed conditions T3 = control (10 mL of distilled water) and T4 = drought stress (10 mL of 18% PEG 6000). The petri dishes were incubated in a growth chamber at 20 °C in the darkness.

Traits scoring: Seed germination was scored at 24-h intervals on daily basis up to 12 days. The seeds were considered germinated when the radicle reached at least 2 mm in length. After 12 days, the experiment was terminated. Several germination and seedling-establishment parameters were evaluated in accordance with the rules of the International Seed Testing Association (ISTA).

##### Measurement of traits

Twenty‑two morphological parameters related to seed germination and seedling establishment were recorded from three biological replicates for each accession under each treatment. The names and abbreviations of all evaluated traits are listed in Table 1.

**Table 1:** Full names, abbreviations and equations for all traits of seed germination and seedling growth in wheat

Abbreviation					
	Unprimed conditions		Nano primed conditions		
Treatments	Control	Drought	Drought and Nano	Control and Nano	Description
Traits					
1.Final Germination Percentage (%)	FG %_C	FG %_D	FG %_DN	FG %_CN	$$FG \%=\frac{Number of germinated grains at {12}^{th}day }{Total number of grains}\times 100$$
2.Initial Germination Percentage (%)	IGP_C	IGP_D	IGP_DN	IGP_CN	$$IG \%=\frac{Number of germinated grains at {1}^{th}day }{Total number of grains}\times 100$$
3.Germination Pace (%)	GP_C	GP_D	GP_DN	GP_CN	$GP=\frac{N}{\sum n\times g}\times 100$
4.Mean Germination Time (hours)	MGT_C	MGT_D	MGT_DN	MGT_CN	$MGT=\sum \frac{nd}{n}$
5.Mean Germination Rate (day^−1^)	MGR_C	MGR_D	MGR_DN	MGR_CN	$MGR=\frac{1}{MGT}$
6.Uncertainity of Germination Process	U_C	U_D	U_DN	U_CN	$U=-\sum_{i=1}^{k}{f}_{i} {\mathit{log}}_{2}{f}_{i}$, being${f}_{i}=\frac{{n}_{i}}{\sum_{i=1}^{k}{n}_{i}}$
7.Synchrony of Germination process	Z_C	Z_D	Z_DN	Z_CN	$$Z=\frac{\sum_{i=1}^{k}{C}_{{n}_{i},2}}{{C}_{\sum {n}_{i},2}}$$, being$${C}_{{n}_{i},2}=\raisebox{1ex}{${n}_{i}({n}_{i}-1)$}\!\left/ \!\raisebox{-1ex}{$2$}\right.$$
8.Coefficient of Variation of Germination Time	CVt_C	CVt_D	CVt_DN	CVt_CN	$C{V}_{t}=\frac{{s}_{t}}{\overline{t} } 100$
9.Seed Vigor Index (%)	SVI_C	SVI_D	SVI_DN	SVI_CN	$SVI=Seedling Length\times G\%$
10.Fresh Weight (g)	FW_C	FW_D	FW_DN	FW_CN	The weight of germinated grains (including root and shoot)
11.Shoot Length (cm)	SL_C	SL_D	SL_DN	SL_CN	The distance between the top of the seed tip to the end of the shoot
12.Root Length (cm)	RL_C	RL_D	RL_DN	RL_CN	The distance between the bottom of the seed tip to the end of the root
13.Root Number	RNo_C	RNo_D	RNo_DN	RNo_CN	The actual count of the number of roots
14.Shoot Root Ratio	SRR_C	SRR_D	SRR_DN	SRR_CN	The ratio of the shoot length to the root length
15.Fresh Weight Drought Tolerance Index	FWDTI_D		FWDTI_DN		$DTI=\frac{FW under drought}{FW under control}\times 100$
16.Shoot Length Drought Tolerance Index	SLDTI_D		SLDTI_DN		$DTI=\frac{SL under drought}{SL under control}\times 100$
17.Root Length Drought Tolerance Index	RLDTI_D		RLDTI_DN		$DTI=\frac{RL under drought}{RL under control}\times 100$
18.Root Number Drought Tolerance Index	RNoDTI_D		RNoDTI_DN		$DTI=\frac{RNo under drought}{RNo under control}\times 100$
19.Reduction of Fresh Weight (g)	RFW_D		RFW_DN		$RFW=FW under control- FW under drought$
20.Reduction of Shoot Length (cm)	RSL_D		RSL_DN		$RSL=SL under control- SL under drought$
21.Reduction of Root Length (cm)	RRL_D		RRL_DN		$RRL=RL under control- RL under drought$
22.Reduction of Root Number	RRNo_D		RRNo_DN		$RRNo=RRNo under control- RRNo under drought$

C and CN denote Control without and with Nano priming, respectively, while D and DN denote Drought without and with Nano priming, respectively

Seed germination parameters such as final and initial germination percentage (FG % and IG %), germination pace (GP), mean germination time (MGT), mean germination rate (MGR), uncertainty of the germination process (U), synchrony of germination (Z), coefficient of variation of germination time (CVt), and seed vigor index (SVI) were calculated for the four treatments. Seedling establishment parameters, including shoot and root lengths (SL and RL), were evaluated using the rolling paper method as described by Hetz *et al.* [44]. For this purpose, 20 grains from each genotype were germinated, and the resulting rolls were placed in 1 L beakers containing the appropriate solutions (0% PEG 6000 for control and 18% PEG 6000 for drought stress). To ensure consistent drought stress and solution volume, the solutions were refreshed every two days until the completion of the experiment. The shoot–root ratio (SRR) was calculated for each genotype as the ratio of shoot length to root length (cm) under the four treatments. Root number (RNo) was visually determined for each genotype as the total number of roots per seedling on the 12th day. Fresh biomass weight (FW) was recorded for each genotype (g) by weighing the germinated seedlings, including both shoots and roots, using a sensitive digital balance (Sartorius AC 1215, Germany). Drought tolerance indices and reduction percentages were calculated under both unprimed and nano‑primed conditions for fresh weight (FWDTI and RFW), shoot length (SLDTI and RSL), root length (RLDTI and RRL), and root number (RNoDTI and RRNo). A detailed description of the calculated reduction parameters, drought tolerance indices, and phenotypic trait measurements is provided in Table 1.

### Statistical analysis of the data

Phenotypic data for the two parents and the population lines were subjected to analysis of variance (ANOVA) and correlation analysis to assess the phenotypic variation. The parental lines were included in these phenotypic data for a comprehensive assessment. However, they were strictly excluded from the QTL analysis.

#### Phenotypic data analysis

Analysis of variance (ANOVA) and broad-sense heritability (${H}^{2}$) were calculated using PLABSTAT software [45]. A statistical model was employed to analyze the phenotypic data within each of the four treatments separately:

$${Y}_{ij}=\mu +{g}_{i}+{r}_{j}+g{r}_{ij}$$

Where ${Y}_{ij}$ is the observation of genotype *i* in replication *j*, *µ* is the general average, ${g}_{i}$ and ${r}_{j}$ are the main effects of genotypes and replication, respectively. The $g{r}_{ij}$ is the interaction between genotype *i* and replication *j*. For this data, genotypes and replications were considered random effects. Broad-sense heritability (${H}^{2}$) was estimated for each trait on an entry-mean basis across three replications for each treatment separately and were calculated ِusing the following equation:

$${H}^{2}=\frac{{{\sigma }^{2}}_{G}}{{{\sigma }^{2}}_{G}+(\frac{{{\sigma }^{2}}_{GR}}{r})}$$

where ${{\sigma }^{2}}_{G}$ is the genotypic variance and $(\frac{{{\sigma }^{2}}_{GR}}{r})$ is the phenotypic variance.

Microsoft Office Excel 365 [46] and R software [47] were used to compute Pearson correlation coefficient for data analysis and visualization.

### QTL mapping

A total of 3457 SNP markers distributed on all the chromosomes were used for linkage map construction, producing a total map length of 2601.11 cM. The A, B, and D sub-genomes harbored different numbers of SNPs, 1476 (1.246 per cM), 1810 (1.64 per cM) and 171 SNP (0.54 per cM). The SNP markers ranged from 7 (chromosome 4D) to 391 (chromosome 5B) (Table 2). The genetic map was used to identify significant associations between single SNP markers and germination‑ and seedling‑establishment traits.

**Table 2:** Summary of chromosome number, number of SNPs per chromosome, number of SNPs per genome, map length, marker coverage density and map density in a double haploid population of wheat

Chromosome	No. of Markers	Map Length (cM)	Marker Coverage Density	Map Density
-Ch1-1A	142	107.31	1.32	0.76
-Ch2-1B	210	118.81	1.77	0.57
-Ch3-1D	75	101.17	0.74	1.35
-Ch4-2A	112	79.18	1.41	0.71
-Ch5-2B	276	148.85	1.85	0.54
-Ch6-2D	13	59.33	0.22	4.56
-Ch7-3A	246	222.42	1.11	0.90
-Ch8-3B	214	211.02	1.01	0.99
-Ch9-3D	9	1.91	4.71	0.21
-Ch10-4A	162	150.00	1.08	0.93
-Ch11-4B	147	119.52	1.23	0.81
-Ch12-4D	7	13.66	0.51	1.95
-Ch13-5A	264	238.79	1.11	0.90
-Ch14-5B	391	201.16	1.94	0.51
-Ch15-5D	25	13.77	1.82	0.55
-Ch16-6A	265	156.58	1.69	0.59
-Ch17-6B	303	133.04	2.28	0.44
-Ch18-6D	25	29.95	0.83	1.20
-Ch19-7A	285	230.09	1.24	0.81
-Ch20-7B	269	167.73	1.60	0.62
-Ch21-7D	17	96.82	0.18	5.70
A genome	1476	1184.37	1.25	0.80
B genome	1810	1100.13	1.65	0.61
D genome	171	316.61	0.54	1.85
Total	3457	2601.11	1.33	0.75

To identify candidate QTLs, the mean phenotypic values of the 98-genotype subset (with 15 lines excluded due to low quality marker data) were analyzed alongside genetic markers. This analysis was performed using the Inclusive Composite Interval Mapping (ICIM) approach implemented in the QTL ICIMapping software, version 4.2.53 [48]. Kosambi mapping function has been used for localization of markers. QTL identification was performed using a default LOD score of 2.5 as an initial threshold. To determine the statistical significance of the detected QTLs, a 1000-permutation test was conducted at a significance level of *P* < 0.05, which yielded trait-specific thresholds ranging from LOD 3.1 to 3.3. In this study, QTLs meeting or exceeding these permutation-derived thresholds were classified as significant QTLs. Additionally, to avoid overlooking potentially important loci with minor effects, QTLs with a LOD score between 2.0 and 3.1 were reported as exploratory QTLs. This dual-threshold approach ensures a balance between statistical rigor and the detection of minor-effect QTLs.

### Mining for candidate genes (Gene annotation and gene expression)

To predict candidate genes associated with the identified QTLs, flanking sequences of the closest linked markers were obtained from Grain Genes http://graingenes.org/index.html, (Visited at 23–9–2025) and were used to blast the database of the Chinese Spring reference genome (IWGSC RefSeqv1.1, https://urgi.versailles.inra.fr/blast_iwgsc/blast.php, (Visited at 15–10–2025) assembly from Ensemble Plants http://plants.ensembl.org/index.html, (Visited at 15–10–2025) and their physical intervals on the reference genome sequences. Then, the annotation of genes was found in the physical intervals of the SNP markers in Ensemble Plants http://plants.ensembl.org/index.html, (Visited at 20–10–2025) and Knetminer https://app.knetminer.com/plants-lite/Triticum_aestivum, (Visited at 5–11–2025). The gene expression values were calculated as the transcript per million (tpm), for each gene under control and abiotic stress based on previously mapped RNA-sequences from the RefSeqv1.1 www.wheatexpression.com, (Visited at 5–11–2025).

### Network analyses

#### Overview of candidate gene filtering and network construction strategy

**Fig. 2:**
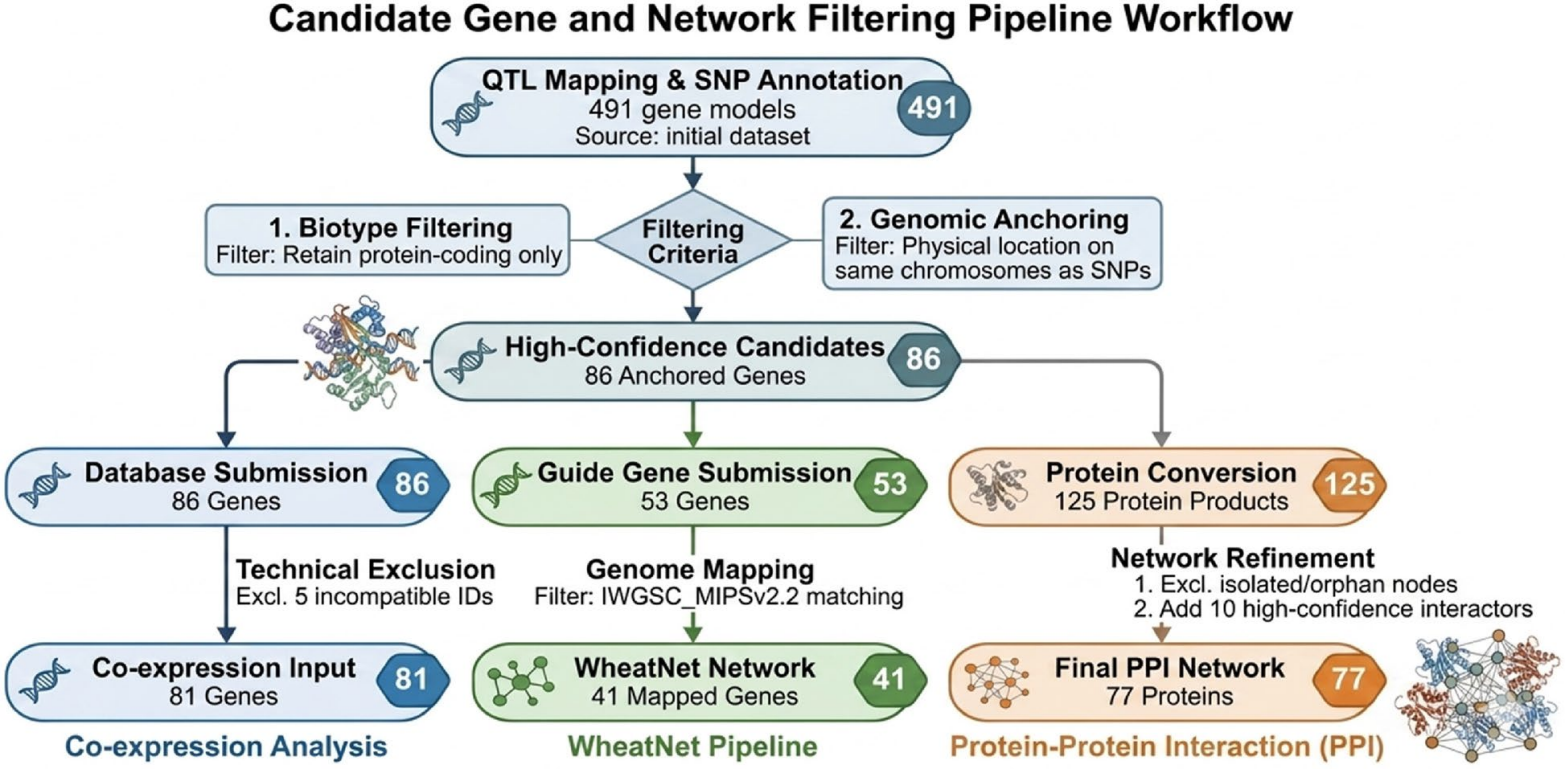
Schematic illustration of the candidate gene filtering and network construction pipeline. SNP annotation following QTL mapping yielded an initial set of 491 gene models, which was progressively filtered based on biotype and genomic anchoring to identify 86 high-confidence candidate genes. These genes were subsequently allocated to co-expression, WheatNet, and protein–protein interaction (PPI) analyses according to database compatibility. Technical exclusions, genome mapping constraints, and network refinement steps resulted in final datasets of 81 genes for co-expression analysis, 41 genes for WheatNet network construction, and 77 proteins for the refined PPI network

Through the annotation of SNPs identified in QTL mapping, an initial set of 491 gene models was generated. Of these, 486 were protein-coding. To apply a strict biological filter, we narrowed this list down to 86 high-confidence candidate genes that were physically anchored to the same chromosomes as the identified SNPs. Subsequent network analyses utilized specific subsets of these 86 anchored genes based on database compatibility requirements. For general co-expression analysis, 5 genes were excluded due to incompatible ID versions, leaving 81 genes for analysis. For the specialized WheatNet pipeline, a subset of 53 genes was selected from the 86 anchored candidates and submitted; of these, 41 genes were successfully recognized and mapped to the IWGSC_MIPSv2.2 reference genome (invalid or unmapped IDs were excluded to prevent disconnected or unsupported nodes within the network structure). Finally, for the Protein–Protein Interaction (PPI) analysis, the 86 anchored genes yielded 125 protein products. This initial interactome was refined by excluding isolated or disconnected nodes (biologically non-informative proteins) to focus on the functionally connected cluster. To further enhance the robustness and significance of this core network, 10 high-confidence interactor proteins were added, resulting in a final, statistically significant PPI interactome of 77 proteins (Fig. 2).

Gene prioritization was performed using WheatNet https://www.inetbio.org/wheatnet/, (Visited at 8–11–2025) under the “Drought Conditions” option. Genes identified from the QTL analysis were submitted as guide genes to predict additional candidate genes functionally connected within the drought-related network context. WheatNet aggregates candidate genes into groups based on network connectivity and ranks them by summing the edge scores directly linked to the guide genes.

Gene co-expression data were obtained from the Wheat Gene Regulatory Network (WGRN) https://wheat.cau.edu.cn/wGRN/w9/, (Visited at 15–11–2025). Co-expressed partners of the candidate genes were extracted based on WGRN’s integrated correlation scores, and a moderate threshold of 0.6 was applied to retain co-expressed gene pairs. The resulting co-expression network was analyzed to identify highly connected gene clusters and potential regulatory modules, and central (hub) genes were noted as they may play key roles in regulating drought-related pathways. These co-expression clusters and hub genes were subsequently used to support functional interpretation of candidate genes and their network relationships.

Protein–protein interaction (PPI) analysis was performed using STRING https://string-db.org/, (Visited at 20–11–2025) to explore interactions among the protein products of the selected candidate genes. High-confidence interactions were retrieved and prepared for network construction to investigate key protein relationships relevant to drought stress. The resulting PPI network was analyzed to identify clusters of interacting proteins and central (hub) proteins, which may play important roles in drought-related pathways.

Hub genes and proteins were identified separately in the co-expression and PPI networks, respectively. Genes whose protein products were hubs in the PPI network and appeared as hubs in the co-expression network were noted as potentially central candidates, highlighting their importance in both protein interactions and gene expression contexts.

Additional gene/protein relationships and functional associations were retrieved from KnetMiner https://app.knetminer.com/plants-lite/Triticum_aestivum, (Visited at 23–11–2025) to expand the interaction landscape of the candidate genes. The candidate genes were subsequently classified into functional categories, including metal transporters, transcriptional regulators, signaling components, and genes involved in metabolic and enzymatic activities. Based on this classification, multiple functional subnetworks were constructed for each category. These subnetworks captured gene–gene, gene–protein, and protein–trait associations relevant to drought response. Each subnetwork was visualized to explore functional connectivity and to elucidate regulatory relationships among genes, proteins, and associated phenotypic traits.

All networks generated from WheatNet, WGRN, and STRING were imported into Cytoscape https://cytoscape.org/, (Visited at 25–11–2025) for visualization and topological analysis. Cytoscape was used to examine network structure, compare connectivity patterns, and highlight key gene clusters, hub genes, and protein interactions.

## Results

### Phenotypic variation and evaluation under all treatments

The frequency distribution plots generated for all traits under the four treatments (control and drought, with and without nano-priming) showed clear patterns of phenotypic variation among genotypes. All traits were negatively affected by drought stress in both unprimed and nano-primed conditions, although the reduction was consistently milder in nano-priming conditions, indicating the beneficial role of ZnO-NPs in improving early growth performance under water deficit. Most traits exhibited approximately normal distribution under all treatments except most germination-related traits under control treatments and FG % under drought treatments, supporting their quantitative nature and suitability for QTL analysis. Overall, the continuous variation and wide distribution ranges across treatments highlight the strong genotype-dependent responses to drought stress and nano-priming (Figures S1–S3).

#### Analysis of variance (ANOVA)

All the evaluated traits showed highly significant variation in all treatments (*P* < 0.01) (Tables 3, 4 and 5). The analysis of variance (ANOVA) revealed high genetic differences among genotypes for all traits under all treatments (Tables 3, 4 and 5). The replications effect was not significant, except for U (*P* < 0.05) under control unprimed, IGP (*P* < 0.1) and MGR (*P* < 0.05) under control primed, Z (*P* < 0.1), CVt (*P* < 0.1), RL (*P* < 0.05) under drought unprimed, RRL (*P* < 0.1), and RRNo (*P* < 0.1) under unprimed and nano primed conditions, respectively (Table 3, 4 and 5).

**Table 3:** Analysis of variance (Single ANOVA) and Heritability for all traits under control conditions with and without nano priming

Treatments	Control Conditions							
Sources of variance	Genotypes (G)		Replicates (R)		RG		H^2^	
DF	114		2		228			
Traits	Unprimed	Nano priming	Unprimed	Nano priming	Unprimed	Nano priming	Unprimed	Nano priming
FW	552.63**	701.06**	0.09	0.11	0.0000	0.0000	99.82	99.86
SL	125.59**	123.06**	0.76	0.35	0.0053	0.0046	99.20	99.19
RL	986.06**	609.59**	0.14	0.69	0.0041	0.0055	99.90	99.84
RNo	35.65**	48.31**	0.03	0.78	0.0014	0.0014	97.20	97.93
FG %	322.91**	14.47**	1.09	1.00	0.0030	0.0811	99.69	93.09
IG %	10.66**	149.26**	0.55	2.36 +	2.4014	0.0801	90.62	99.33
GP	8.07**	51.21**	1.03	1.50	1.9025	0.1509	87.61	98.05
SVI	668.33**	484.24**	0.81	0.26	98.0024	104.7835	99.85	99.79
SRR	667.37**	292.32**	0.43	1.05	0.0001	0.0001	99.85	99.66
MGR	33.98**	15.22**	1.14	4.09*	0.0001	0.0001	97.06	93.43
MGT	301.51**	911.21**	0.41	0.32	0.0055	0.0007	99.67	99.89
U	389.80**	54.45**	3.62*	1.71	0.0001	0.0003	99.74	98.16
Z	34.34**	29.70**	0.41	1.42	0.0002	0.0001	97.09	96.63
CVt	84.13**	476.77**	1.90	0.44	0.0071	0.1137	99.99	99.79

*FW* Fresh Weight, *SL* Shoot Length, *RL* Root Length, *RNo* Root Number, *FG* *%* Final Germination Percentage, *IG* *%* Initial Germination Percentage, *GP* Germination Pace, *SVI* Seed Vigor Index, *SRR* Shoot/Root Ratio, *MGT* Mean Germination Time, *MGR* Mean Germination Rate, *U* Uncertainty ofGermination Process, *Z* Synchrony of Germination Process, and *CVt* Coefficient of Variation of Germination Time

The degree of significance is indicated as ^+^*P*,0.1; *P,0.05; **P,0.01; ***P,0.001

With and without nano priming, the heritability estimates for traits under control were higher than their corresponding values under drought (Tables 3 and 4). Under unprimed control conditions *H*^*2*^ ranged from 87.61 for GP to 99.99 for CVt and under nano priming control conditions *H*^*2*^ ranged from 93.09 for FG % to 99.89 for MGT (Table 3). Similarly, under unprimed drought conditions *H*^*2*^ ranged from 93.1 for MGT to 99.57 for SL and under nano priming drought conditions *H*^*2*^ ranged from 93.15 for Z to 99.54 for SL (Table 4). The heritability estimates for drought tolerance index and reduction traits under unprimed and nano primed were very high ranged from 97.57 for RRNo to 99.84 for RLDTI and from 97.84 for RNoDTI to 99.78 for RLDTI, respectively (Table 5).

**Table 4:** Analysis of variance (Single ANOVA) & Heritability for all traits under drought conditions with and without nano priming

Treatments	Drought Conditions							
Sources of variance	Genotypes (G)		Replicates (R)		RG		H^2^	
Traits	14		2		228			
Traits	Unprimed	Nano priming	Unprimed	Nano priming	Unprimed	Nano priming	Unprimed	Nano priming
FW	170.10**	136.25**	1.13	1.00	0.0000	0.0000	99.41	99.27
SL	232.52**	215.85**	0.41	0.29	0.0036	0.0033	99.57	99.54
RL	109.80**	202.97**	3.09*	2.06	0.0087	0.0050	99.09	99.51
RNo	85.99**	92.12**	1.45	0.76	0.0006	0.0007	98.84	98.91
FG %	160.59**	95.42**	0.20	1.00	0.0340	0.0203	99.38	98.95
IG %	16.98**	18.91**	0.85	1.02	5.3789	4.4928	94.11	94.71
GP	22.57**	60.25**	0.16	0.50	1.7839	0.7061	95.57	98.34
SVI	56.34**	93.30**	1.77	0.30	479.0371	293.0037	98.23	98.93
SRR	112.80**	106.05**	0.82	1.13	0.0000	0.0000	99.11	99.06
MGR	15.00**	48.74**	1.76	0.10	0.0003	0.0001	93.33	97.95
MGT	14.49**	88.21**	1.93	0.34	0.6544	0.0892	93.10	98.87
U	21.12**	95.85**	1.25	2.00	0.0018	0.0004	95.26	98.96
Z	17.19**	14.60**	2.90 +	0.98	0.0005	0.0006	94.18	93.15
CVt	96.20**	162.75**	2.94 +	0.97	0.3393	0.2613	98.96	99.39

*FW* Fresh Weight, *SL* Shoot Length, *RL* Root Length, *RNo* Root Number, *FG* *%* Final Germination Percentage, *IG* *%* Initial Germination Percentage, *GP* Germination Pace, *SVI* Seed Vigor Index, *SRR* Shoot/Root Ratio, *MGT* Mean Germination Time, *MGR* Mean Germination Rate, *U* Uncertainty ofGermination Process, *Z* Synchrony of Germination Process, and *CVt* Coefficient of Variation of Germination Time

The degree of significance is indicated as ^+^*P*,0.1; *P,0.05; **P,0.01; ***P,0.001

**Table 5:** Analysis of variance (Single ANOVA) & Heritability for drought tolerance index and reduction traits under unprimed and nano priming conditions

Sources of variance	Genotypes (G)		Replicates (R)		RG		H^2^	
DF	114		2		228			
Traits	Unprimed	Nano priming	Unprimed	Nano priming	Unprimed	Nano priming	Unprimed	Nano priming
Treatments								
FWDTI	134.54**	189.46**	0.26	0.02	0.4469	0.3755	99.26	99.47
SLDTI	166.04**	201.33**	0.2	0.35	0.4542	0.3438	99.40	99.50
RLDTI	610.77**	463.32**	0.48	0.16	1.6215	0.847	99.84	99.78
RNoDTI	67.65**	46.32**	0.22	1.66	0.6657	1.095	98.52	97.84
RFW	236.13**	251.29**	0.55	0.15	0.0001	0.0001	99.58	99.60
RSL	131.48**	193.13**	0.24	0.08	0.0098	0.0074	99.24	99.48
RRL	311.51**	355.99**	2.46 +	0.29	0.0133	0.0114	99.68	99.72
RRNo	41.07**	49.97**	0.02	2.42 +	0.0019	0.0018	97.57	98.00

*FW* Fresh Weight, *SL* Shoot Length, *RL* Root Length, *RNo* Root Number, *R* Reduction, and *DTI* Drought Tolerance Index

The degree of significance is indicated as ^+^*P*,0.1; *P,0.05; **P,0.01; ***P,0.001

#### Correlation analysis

Pearson’s correlation analysis among the studied traits under all treatments revealed distinct differences in their responses to drought stress under both nano‑primed and unprimed conditions.

Under control conditions without ZnO-NPs, most correlations were moderate to high and statistically significant (P ≤ 0.05 and P ≤ 0.01). Mostly, the correlations between the germination-related traits and the seedling traits were positive and significant. The germination-related traits showed positive correlations among each other than the seedling-related traits. U_C and CVt_C had the highest significant and positive correlations with *r* = 0.96***. MGT_C showed the highest negative significant correlations with MGR_C with *r* = – 0.97*** (Figure S4a). Under control with ZnO-NPs, a similar pattern was observed, and the positive correlations were higher than their corresponding values under control without ZnO-NPs. For example, U_CN and CVt_CN had the highest significant and positive correlations with *r* = 0.95***. MGT_CN showed the highest negative significant correlations with GP_CN with *r* = – 0.96*** (Figure S4b). Most seedling related traits were moderately positively and highly significantly correlated with each other except RL and SRR. The correlation between germination and seedling related traits was positive except SVI, MGT, U, and CVt.

Under drought conditions without ZnO-NPs, the germination-related traits showed highly significant correlations. For example, Z_D showed negative significant correlations with U_D (*r* = – 0.92***), while MGR_D was positively correlated with GP_D (*r* = 0.95***) (Figure S5a). Under drought with ZnO-NPs, a similar pattern was observed, and the positive correlations were higher than their corresponding values under control without ZnO-NPs. For example, GP_DN and MGR_DN had the highest significant and positive correlations with *r* = 0.98***. MGT_DN showed the highest negative significant correlations with GP_DN with *r* = – 0.96*** (Figure S5b).

Drought tolerance indices showed strong negative correlations with reduction traits (e.g., SLDTI and RSL, *r* = –0.97*** under nano priming and *r* = – 0.95*** under unprimed conditions) and strong positive correlations with seedling traits such as SL (*r* = 0.88*** under nano priming and *r* = 0.85*** under unprimed) (Figure S6).

Overall, these findings indicate that germination traits are tightly associated, while seedling and drought tolerance indices exhibit consistent interrelationships reflecting adaptive responses to drought stress.

### QTL mapping

Quantitative trait loci (QTL) mapping, through Inclusive Composite Interval Mapping (ICIM-ADD) was performed for all 22 germination and seedling-establishment parameters under the four treatments.

**Fig. 3:**
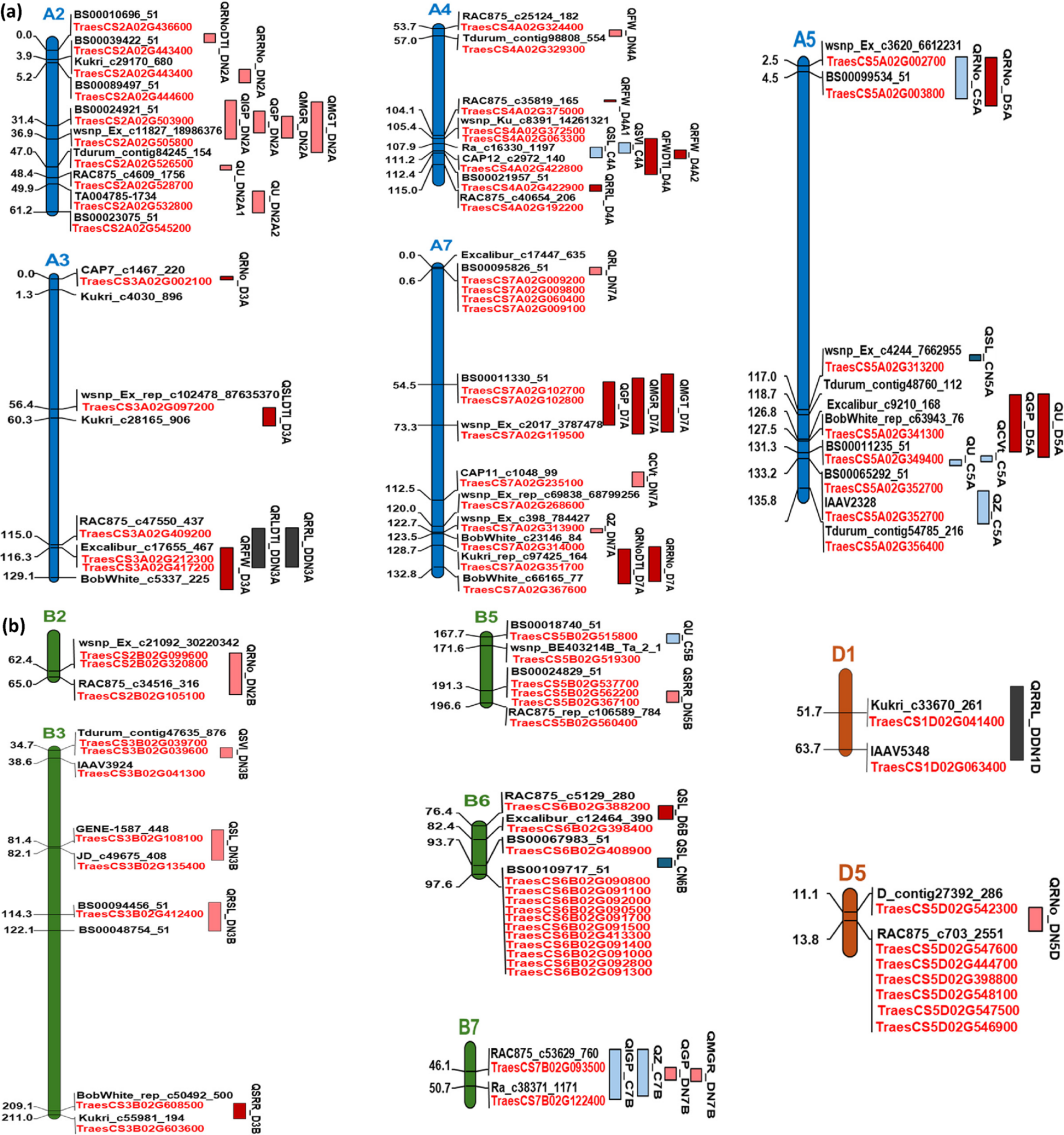
QTL mapping and localization of genes for germination and seedling-establishment traits under control and drought conditions with and without nano priming. Marker locus name and positions (cM) are located to the left and right of the vertical bars, respectively. **a** QTL mapping and localization of genes for genome A. **b** QTL mapping and localization of genes for genome B and D

In total, 51 QTLs associated with wheat germination and seedling‑establishment traits were identified, explaining 1.52–17.91% of the phenotypic variation, with the lowest and highest contributions observed for *QRFW_D3A* and *QRFW_D4A*, respectively. The chromosomal positions of all detected QTLs are illustrated in Fig. 3, while detailed information on significant and exploratory QTLs, including additive effects, flanking markers, phenotypic variation explained (PVE %), and logarithm of odds (LOD) scores, is summarized in Table 6. The LOD scores ranged from 2.03 to 16.66, and the identified QTLs were distributed across 12 chromosomes, namely 2A, 3A, 4A, 5A, 7A, 2B, 3B, 5B, 6B, 7B, 1D, and 5D. Chromosomes 2A, 5A, and 7A harbored the highest number of QTLs, with eight QTLs detected on each chromosome, followed by chromosome 4A with seven QTLs and chromosome 3A with five QTLs. Chromosomes 3B and 7B each contained four QTLs, whereas chromosomes 5B and 6B each harbored two QTLs. The lowest number of QTLs (one per chromosome) was detected on chromosomes 2B, 1D, and 5D. Based on the portion of phenotypic variation explained by each QTL, they are classified into three classes: 30 major QTLs (PVE = 10–20%), 20 moderate QTLs (PVE = 5–10%), and one minor QTLs (PVE < 5%).

**Table 6:** Additive QTLs for germination and seedling establishment parameters in wheat DH lines primed with ZnO-NPs under drought stress

	QTL	Trait Name	Chromosome		Position	Left Marker	Right Marker	LOD	PVE (%)	Add	LeftCI	RightCI	Environment	QTL Type
1	*QSL_C4A*	SL_C	10	4A	111	Ra_c16330_1197	CAP12_c2972_140	2.79	13.46	−0.51	107.50	112.50	Control	Exploratory
2	*QRNo_C5A*	RNo_C	13	5A	4	wsnp_Ex_c3620_6612231	BS00099534_51	2.61	11.71	0.14	2.50	16.50	Control	Exploratory
3	*QIG %_C7B*	IG %_C	20	7B	50	RAC875_c53629_760	Ra_c38371_1171	2.18	9.92	3.03	38.50	54.50	Control	Exploratory
4	*QSVI_C4A*	SVI_C	10	4A	111	Ra_c16330_1197	CAP12_c2972_140	2.50	11.55	−149.12	107.50	112.50	Control	Exploratory
5	***QU_C5A***	**U_C**	**13**	**5A**	**133**	**BS00011235_51**	**BS00065292_51**	**4.12**	13.17	**0.13**	**131.50**	**133.50**	**Control**	**Significant**
6	*QU_C5B*	U_C	14	5B	171	BS00018740_51	wsnp_BE403214B_Ta_2_1	2.76	8.55	−0.11	167.50	171.50	Control	Exploratory
7	*QZ_C5A*	Z_C	13	5A	134	IAAV2328	Tdurum_contig54785_216	2.21	9.29	−0.05	131.50	142.50	Control	Exploratory
8	*QZ_C7B*	Z_C	20	7B	50	RAC875_c53629_760	Ra_c38371_1171	2.14	9.04	0.05	40.50	55.50	Control	Exploratory
9	*QCVt_C5A*	CVt_C	13	5A	133	BS00011235_51	BS00065292_51	2.77	14.04	5.66	131.50	133.50	Control	Exploratory
10	*QSL_D6B*	SL_D	17	6B	82	RAC875_c5129_280	Excalibur_c12464_390	2.44	12.42	−0.60	76.50	82.50	Drought	Exploratory
11	*QRNo_D3A*	RNo_D	7	3A	1	CAP7_c1467_220	Kukri_c4030_896	2.60	11.52	0.15	0.00	1.50	Drought	Exploratory
12	*QRNo_D5A*	RNo_D	13	5A	3	wsnp_Ex_c3620_6612231	BS00099534_51	2.12	8.53	0.13	1.50	17.50	Drought	Exploratory
13	*QGP_D5A*	GP_D	13	5A	127	Excalibur_c9210_168	BobWhite_rep_c63943_76	2.19	8.01	−3.52	118.50	137.50	Drought	Exploratory
14	*QGP_D7A*	GP_D	19	7A	63	BS00011330_51	wsnp_Ex_c2017_3787478	2.84	13.59	4.64	53.50	73.50	Drought	Exploratory
15	*QSRR_D3B*	SRR_D	8	3B	211	BobWhite_rep_c50492_500	Kukri_c55981_194	2.09	9.39	0.04	203.50	211.00	Drought	Exploratory
16	*QMGR_D7A*	MGR_D	19	7A	62	BS00011330_51	wsnp_Ex_c2017_3787478	2.33	10.75	0.04	50.50	76.50	Drought	Exploratory
17	*QMGT_D7A*	MGT_D	19	7A	62	BS00011330_51	wsnp_Ex_c2017_3787478	2.27	10.68	−1.98	49.50	76.50	Drought	Exploratory
18	*QU_D5A*	U_D	13	5A	127	Excalibur_c9210_168	BobWhite_rep_c63943_76	2.04	9.12	0.11	118.50	139.50	Drought	Exploratory
19	*QFW_DN4A*	FW_DN	10	4A	56	RAC875_c25124_182	Tdurum_contig98808_554	2.61	11.73	−0.05	53.50	56.50	Drought and Nano	Exploratory
20	*QSL_DN3B*	SL_DN	8	3B	82	GENE-1587_448	JD_c49675_408	2.25	10.07	0.50	74.50	89.50	Drought and Nano	Exploratory
21	*QRL_DN7A*	RL_DN	19	7A	0	Excalibur_c17447_635	BS00095826_51	2.19	10.10	0.60	0.00	3.50	Drought and Nano	Exploratory
22	*QRNo_DN2B*	RNo_DN	5	2B	63	wsnp_Ex_c21092_30220342	RAC875_c34516_316	2.09	10.73	−0.16	46.50	64.50	Drought and Nano	Exploratory
23	*QRNo_DN5D*	RNo_DN	15	5D	13	D_contig27392_286	RAC875_c703_2551	2.19	11.06	0.16	5.50	13.00	Drought and Nano	Exploratory
24	*QIG %_DN2A*	IGP_DN	4	2A	36	BS00024921_51	wsnp_Ex_c11827_18986376	2.34	10.39	5.48	29.50	45.50	Drought and Nano	Exploratory
25	***QGP_DN2A***	**GP_DN**	**4**	**2A**	**34**	**BS00024921_51**	**wsnp_Ex_c11827_18986376**	**3.18**	12.29	**4.40**	**31.50**	**40.50**	**Drought and Nano**	**Significant**
26	*QGP_DN7B*	GP_DN	20	7B	50	RAC875_c53629_760	Ra_c38371_1171	2.62	9.69	3.89	46.50	50.50	Drought and Nano	Exploratory
27	*QSVI_DN3B*	SVI_DN	8	3B	38	Tdurum_contig47635_876	IAAV3924	2.03	9.41	95.04	35.50	40.50	Drought and Nano	Exploratory
28	*QSRR_DN5B*	SRR_DN	14	5B	195	BS00024829_51	RAC875_rep_c106589_784	2.77	11.80	0.05	191.50	196.50	Drought and Nano	Exploratory
29	***QMGR_DN2A***	**MGR_DN**	**4**	**2A**	**34**	**BS00024921_51**	**wsnp_Ex_c11827_18986376**	**3.34**	12.87	**0.04**	**31.50**	**40.50**	**Drought and Nano**	**Significant**
30	*QMGR_DN7B*	MGR_DN	20	7B	50	RAC875_c53629_760	Ra_c38371_1171	2.57	9.53	0.04	46.50	50.50	Drought and Nano	Exploratory
31	*QMGT_DN2A*	MGT_DN	4	2A	35	BS00024921_51	wsnp_Ex_c11827_18986376	2.41	10.50	−1.74	24.50	45.50	Drought and Nano	Exploratory
32	*QU_DN2A1*	U_DN	4	2A	47	Tdurum_contig84245_154	RAC875_c4609_1756	**9.44**	15.02	−0.24	46.50	48.50	Drought and Nano	**Significant**
33	***QU_DN2A2***	**U_DN**	**4**	**2A**	**61**	**TA004785-1734**	**BS00023075_51**	**4.80**	6.82	**0.16**	**52.50**	**61.50**	**Drought and Nano**	**Significant**
34	*QZ_DN7A*	Z_DN	19	7A	123	wsnp_Ex_c398_784427	BobWhite_c23146_84	2.78	12.24	−0.06	121.50	123.50	Drought and Nano	Exploratory
35	*QCVt_DN7A*	CVt_DN	19	7A	118	CAP11_c1048_99	wsnp_Ex_rep_c69838_68799256	2.05	8.24	3.99	112.50	119.50	Drought and Nano	Exploratory
36	***QSL_CN5A***	**SL_CN**	**13**	**5A**	**118**	**wsnp_Ex_c4244_7662955**	**Tdurum_contig48760_112**	**3.26**	11.23	**−0.49**	**116.50**	**118.50**	**Control and Nano**	**Significant**
37	***QSL_CN6B***	**SL_CN**	**17**	**6B**	**95**	**BS00067983_51**	**BS00109717_51**	**5.00**	17.53	**−0.61**	**93.50**	**97.50**	**Control and Nano**	**Significant**
38	*QFWDTI_D4A*	FWDTI_D	10	4A	111	Ra_c16330_1197	CAP12_c2972_140	2.07	13.24	4.58	108.50	125.50	Control vs Drought	Exploratory
39	*QSLDTI_D3A*	SLDTI_D	7	3A	59	wsnp_Ex_rep_c102478_87635370	Kukri_c28165_906	2.09	9.40	5.17	52.50	60.50	Control vs Drought	Exploratory
40	*QRNoDTI_D7A*	RNoDTI_D	19	7A	132	Kukri_rep_c97425_164	BobWhite_c66165_77	2.05	9.49	3.61	125.50	141.50	Control vs Drought	Exploratory
41	*QRFW_D3A*	RFW_D	7	3A	129	Excalibur_c17655_467	BobWhite_c5337_225	2.28	1.53	−0.08	118.50	136.50	Control vs Drought	Exploratory
42	***QRFW_D4A1***	**RFW_D**	**10**	**4A**	**105**	**RAC875_c35819_165**	**wsnp_Ku_c8391_14261321**	**10.45**	**9.67**	**0.20**	**104.50**	**105.50**	**Control vs Drought**	**Significant**
43	***QRFW_D4A2***	**RFW_D**	**10**	**4A**	**111**	**Ra_c16330_1197**	**CAP12_c2972_140**	**16.66**	**17.92**	**−0.27**	**108.50**	**112.50**	**Control vs Drought**	**Significant**
44	*QRRL_D4A*	RRL_D	10	4A	115	BS00021957_51	RAC875_c40654_206	2.47	11.33	−1.23	112.50	115.50	Control vs Drought	Exploratory
45	*QRRNo_D7A*	RRNo_D	19	7A	132	Kukri_rep_c97425_164	BobWhite_c66165_77	2.12	9.82	−0.16	125.50	141.50	Control vs Drought	Exploratory
46	*QRNoDTI_DN2A*	RNoDTI_DN	4	2A	3	BS00010696_51	BS00039422_51	2.45	12.70	−4.33	0.00	3.50	Control Nano vs Drought Nano	Exploratory
47	*QRSL_DN3B*	RSL_DN	8	3B	119	BS00094456_51	BS00048754_51	2.06	9.02	−0.70	108.50	122.50	Control Nano vs Drought Nano	Exploratory
48	*QRRNo_DN2A*	RRNo_DN	4	2A	4	Kukri_c29170_680	BS00089497_51	2.25	12.54	0.18	0.00	5.50	Control Nano vs Drought Nano	Exploratory
49	*QRLDTI_DDN3A*	RLDTI_DDN	7	3A	116	RAC875_c47550_437	wsnp_Ex_c14202_22145805	2.08	10.17	−6.10	109.50	126.50	Drought Nano vs Drought	Exploratory
50	*QRRL_DDN1D*	RRL_DDN	3	1D	53	Kukri_c33670_261	IAAV5348	2.18	9.16	−0.69	39.50	63.50	Drought Nano vs Drought	Exploratory
51	*QRRL_DDN3A*	RRL_DDN	7	3A	116	RAC875_c47550_437	wsnp_Ex_c14202_22145805	2.03	8.39	−0.66	109.50	126.50	Drought Nano vs Drought	Exploratory

*MGT* Mean Germination Time, *MGR* Mean Germination Rate, *U* Uncertainty of Germination Process, *CVt* Coefficient of Variation of Germination Time, *FG %* Final Germination Percentage, *IG %* Initial Germination Percentage, *GP* Germination Pace, *SVI* Seed Vigor Index, *Z* Synchrony of Germination Process, *FW* Fresh Weight, *SL* Shoot Length, *RL* Root Length, *RNo* Root Number, *SRR* Shoot/Root Ratio, *C* Control without nano priming, *D* Drought without nano priming, *DN* Drought with nano priming, *CN* Control with nano priming, *PVE (%)* Percentage of Phenotypic Variance explained by the QTL, and Add = Additive Effect. QTLs that were identified at LOD = 2.5 or 2 and named exploratory and QTLs that passed the permutation threshold were named significant and labelled bold

### QTLs for control with and without priming

Nine QTLs were identified under control conditions without nano‑priming, comprising five major and four moderate QTLs. The QTL *QSL_C4A*, associated with SL on chromosome 4A, explained 13.46% of the phenotypic variation and exhibited a negative additive effect. In contrast, *QRNo_C5A*, controlling RNo on chromosome 5A, showed a positive additive effect, accounting for 11.71% of the phenotypic variation. The QTLs *QU_C5A* (LOD Score = 4.12, classified as significant) and *QCVt_C5A*, both mapped to chromosome 5A and associated with U and CVt, respectively, co‑localized to the same genomic region, indicating a pleiotropic effect; they explained 13.16% and 14.03% of the phenotypic variation, respectively, with positive additive effects. Conversely, *QU_C5B*, located on chromosome 5B and associated with U, explained 8.55% of the phenotypic variation and exhibited a negative additive effect (Table 6; Fig. 3).

Under control conditions with nano-priming, two QTLs (*QSL_CN5A* and *QSL_CN6B*) were detected on chromosomes 5A and 6B for SL with LOD Score = 3.25 and 5.00, respectively, and were classified as significant QTLs. *QSL_CN5A* and *QSL_CN6B* explained 11.23% and 17.52% of the phenotypic variation, respectively, and both exhibited negative additive effects (Table 6; Fig. 3).

### QTLs for drought with and without priming

Similarly, nine QTLs associated with germination and seedling‑establishment traits were identified under drought conditions without nano‑priming, including five major and four moderate QTLs. The QTLs *QRNo_D3A* and *QGP_D7A* were detected on chromosomes 3A and 7A for RNo and GP with LOD scores of 2.59 and 2.84, respectively, and were classified as exploratory QTLs. Both *QRNo_D3A* and *QGP_D7A* showed positive additive effects and explained 11.52% and 13.59% of the phenotypic variation, respectively (Table 6; Fig. 3).

Under drought conditions with nano‑priming, a total of 17 QTLs associated with germination and seedling‑establishment traits were identified, including 12 major and five moderate QTLs. Among these, *QFW_DN4A*, associated with FW on chromosome 4A, *QU_DN2A1,* controlling U on chromosome 2A (LOD = 9.44, classified as significant), and *QZ_DN7A*, associated with Z on chromosome 7A,, exhibited negative additive effects and explained 11.73%, 15.02%, and 12.23% of the phenotypic variation, respectively. In contrast, *QGP_DN2A*, controlling GP on chromosome 2A (LOD = 3.18, classified as significant), *QGP_DN7B*, associated with GP on chromosome 7B, *QMGR_DN2A*, controlling MGR on chromosome 2A (LOD = 3.24, classified as significant), *QMGR_DN7B*, associated with MGR on chromosome 7B, *QU_DN2A2*, associated with U on chromosome 2A (LOD = 4.79, classified as significant), and *QSRR_DN5B*, associated with SRR on chromosome 5B, exhibited positive additive effects, explaining 12.29%, 9.68%, 12.87%, 9.53%, 6.81%, and 11.80% of the phenotypic variation, respectively. Notably, *QGP_DN2A and QMGR_DN2A*, both mapped to chromosome 2A, co‑localized to the same genomic region, indicating a pleiotropic effect. Similarly, *QGP_DN7B and QMGR_DN7B*, located on chromosome 7B, corresponded to the same QTL interval, also exhibiting pleiotropy (Table 6; Fig. 3).

### QTLs for drought tolerance indices and reduction parameters

Fourteen QTLs were identified for drought tolerance indices and reduction parameters. Five QTLs were associated with drought tolerance index traits related to FW, SL, RL, and RNo, whereas nine QTLs were linked to reduction parameters for FW, RL, and RNo. Among these, *QRFW_D4A1* and *QRFW_D4A2*, both detected-on chromosome 4A, were associated with RFW and exhibited LOD scores of 10.45 and 16.66, respectively, and were classified as significant QTLs. The QTL *QRFW_D4A1* showed a positive additive effect and explained 9.66% of the phenotypic variation, whereas *QRFW_D4A2* exhibited a negative additive effect and accounted for 17.91% of the phenotypic variation (Table 6; Fig. 3).

### Gene annotation, expression and mapping of the candidate genes

All flanking markers of the identified QTLs were subjected to BLAST analysis against the Ensembl Plants genomic database to predict candidate genes and their corresponding protein‑coding sequences. In total, 491 distinct gene models were identified across all traits and experimental conditions (Table S1). These candidate gene models encode 486 unique proteins exhibiting diverse biological and molecular functions. Of the 486 annotated genes, 86 gene models were physically anchored to the flanking markers of the detected QTLs, indicating strong concordance between the genetic mapping results and the reference genome annotation. All identified gene models encode functional proteins; however, twelve were annotated as uncharacterized proteins. The predicted proteins encoded by the 86 candidate genes physically anchored to the QTL regions were functionally annotated and classified into several categories, including DNA replication and repair (5 genes), signal transduction and kinase activity (15 genes), protein degradation via the ubiquitin pathway (5 genes), ion transport (5 genes), transcription factors (15 genes), protein folding and chaperone activity (5 genes), secondary metabolism (8 genes), and enzymes involved in various metabolic processes (25 genes).

**Fig. 4:**
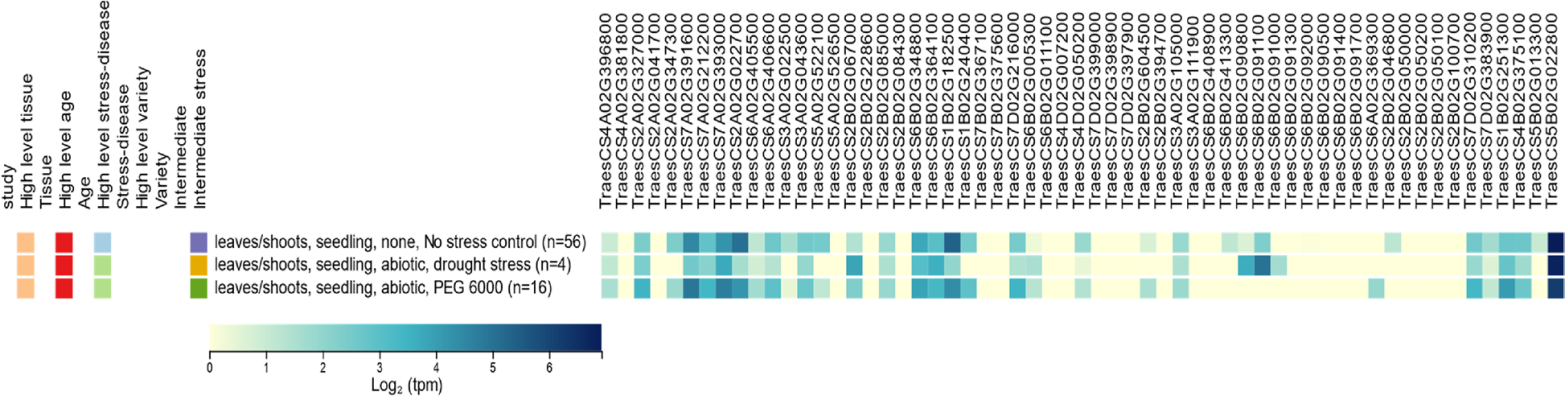
Expression profiles of the candidate genes associated with germination and seedling‑establishment traits in wheat leaves/shoot tissues at the seedling stage under three conditions: control, drought, and PEG‑induced osmotic stress. Expression values were retrieved from the Wheat Expression database, and normalized transcript abundance is shown for each gene across the tested stress treatments

The expression profiles of all identified gene models were obtained from the wheat expression database at the seedling stage under both non‑stress and abiotic stress conditions (Table S2; Fig. 4). Expression analysis revealed that, under PEG 6000‑induced drought stress, 19 genes were upregulated, while 16 genes were downregulated in wheat seedlings. Overall, the gene expression patterns could be categorized into three distinct response profiles: upregulated, downregulated, and stably expressed genes.

Upregulated genes could be classified into two main categories. The first category comprised genes that were expressed under normal conditions but significantly overexpressed during drought stress, indicating the activation of pre‑existing stress‑response mechanisms (e.g., *TraesCS3A02G002100* and *TraesCS5B02G537700*). The second category included inducible genes that were expressed exclusively under drought conditions, suggesting specific activation in response to water deficit (e.g., *TraesCS2A02G503900* and *TraesCS4A02G192200*). Genes in both categories are primarily associated with osmotic adjustment, antioxidant defense, and stress‑signaling pathways.

Downregulated genes also exhibited two distinct expression patterns. Some genes were expressed under control conditions but showed significantly reduced expression under drought stress, reflecting the suppression of growth‑related or metabolic processes (e.g., *TraesCS7A02G102700*, *TraesCS4A02G329300*, and *TraesCS7B02G122400*). In contrast, a second group of genes was completely silenced under drought conditions, suggesting energy‑saving strategies or regulatory shifts favoring stress adaptation (e.g., *TraesCS4A02G375000* and *TraesCS7A02G009800*).

In addition, several genes maintained stable expression levels under both control and drought conditions, indicating constitutive roles in stress tolerance. Such expression stability likely contributes to the maintenance of essential defense and metabolic functions, thereby supporting physiological homeostasis under water‑limited conditions. For instance, *TraesCS5A02G002700*, *TraesCS7A02G314000*, and *TraesCS3B02G608500* exhibited consistent expression despite their association with drought‑responsive pathways, suggesting their contribution to baseline resilience mechanisms.

### Network analyses

#### Gene prioritization (Gene network direct neighborhood)

Gene prioritization analysis identified twelve high‑confidence candidate gene groups exhibiting strong network connectivity with the guide gene set (Figure S7). A total of 53 guide genes were initially submitted to WheatNet for co‑expression network construction, of which 41 genes were successfully recognized and mapped to the IWGSC_MIPSv2.2 reference genome. Invalid or unmapped gene identifiers were excluded prior to analysis to ensure accurate network inference and to prevent the inclusion of disconnected or unsupported nodes within the network structure. All 41 remaining guide genes were confirmed to be present in the WheatNet interactome database, resulting in complete coverage (1.00). The interaction quality assessment demonstrated reliable co‑expression performance, with an area under the receiver operating characteristic curve (AUC) value of 0.63, indicating moderately strong predictive accuracy of the inferred interactions. Notably, the resulting network exhibited high statistical significance (*P* < 0.001). These outcomes collectively suggest the potential suitability of the curated gene set for subsequent co-expression network visualization, clustering analysis, and hub gene identification. Among the prioritized clusters, *Genegroup_2122* ranked first, with the highest network score (7.04), supported by co‑expression and co‑citation evidence, and was connected to three guide gene groups (*Genegroup_5413*, *Genegroup_7609*, and *Genegroup_8357*). Functional enrichment analysis suggested that this group may be primarily associated with responses to cadmium ions and translational elongation. The remaining eleven gene groups exhibited variable network scores and connectivity patterns, with higher‑ranked clusters tending to show stronger co‑expression and co‑citation support compared with lower‑ranked modules. Functional enrichment analysis indicated potential involvement of these groups in diverse stress‑related processes, including oxidation–reduction reactions, protein folding, and translational regulation. Overall, the twelve prioritized gene clusters are predicted to capture both general stress‑responsive signatures and specific regulatory mechanisms that may contribute to cellular stability and protein synthesis under dehydration stress. These findings provide a computational framework and hypothesis‑generating basis for downstream functional characterization and experimental validation.

### Gene co-expression and hub genes identification (Gene–Gene Interaction)

Co‑expression analysis of 81 candidate genes revealed a network consisting of 59 nodes and 283 edges, organized into seven connected components, suggesting coordinated expression among a subset of genes (Figure S8). Node degree distribution and betweenness centrality were analyzed and visualized to assess gene connectivity patterns and to identify putative hub genes within the network (Figure S9). Clustering analysis identified three co‑expressed gene modules, including one large module comprising 20 nodes and 188 edges, as well as two smaller modules containing 3–4 nodes each (Figure S10). The large module is predicted to represent a tightly connected, core drought‑responsive network, whereas the smaller modules may function as peripheral or satellite signaling groups. Network analysis of the 20‑node core module revealed a highly cohesive structure, characterized by a high average number of neighbors, a short characteristic path length, and a clustering coefficient approaching 1, collectively indicating strong putative functional relationships among its constituent genes. This core module exhibited high network density with low heterogeneity and centralization, suggesting a relatively homogeneous structure with uniformly distributed connections. The formation of a single connected component further supports its predicted integrity as a drought‑responsive regulatory module. To prioritize key candidate genes for downstream investigation, the top 20 genes were selected based on network connectivity and centrality measures (Table S3).

### Protein–Protein Interaction (PPI) Network Analysis and hub proteins identification

Protein–protein interaction (PPI) analysis was conducted using the STRING database for the 125 protein products encoded by the prioritized candidate genes. Initially, the PPI network constructed from these proteins comprised 80 nodes and 58 edges, with an average node degree of 1.45 and an average local clustering coefficient of 0.22. This network showed significant enrichment of interactions (PPI enrichment *P* < 0.01), suggesting that the observed associations were more frequent than would be expected by random chance. To enhance network resolution, an additional set of ten high‑confidence interactor proteins was incorporated, resulting in an expanded network of 90 nodes and 365 edges. This updated network showed a markedly increased average node degree (8.11) and clustering coefficient (0.36), along with highly significant interaction enrichment (PPI enrichment *P* = 1.08 × 10⁻⁸), indicating a substantially higher level of predicted connectivity among the proteins. Proteins lacking detectable interactions were subsequently excluded, yielding a refined network of 77 proteins for detailed analysis (Figure S11). The resulting PPI network consisted of 77 nodes and 365 edges, with an average number of neighbors of 9.48, a clustering coefficient of 0.38, network density of 0.12, a diameter of 6, a radius of 3, and a characteristic path length of 2.82, forming a single connected component. Network topology metrics, including node degree distribution and betweenness centrality, were examined to assess connectivity patterns and to identify putative hub proteins within the network (Figure S12). Clustering analysis further revealed four distinct protein modules, which are predicted to represent groups of proteins potentially acting in shared or related biological processes (Figure S13). The largest module comprised 14 nodes and 27 edges, while the remaining modules contained 10, 6, and 4 nodes, respectively. Collectively, these modules are inferred to capture a core drought‑responsive interaction framework, whereas the smaller clusters may correspond to peripheral or auxiliary signaling components within the broader network. To prioritize proteins for downstream investigation, the top 20 candidates were selected based on connectivity and centrality metrics (Table S4), representing high‑confidence targets for future functional validation.

### Functional enrichment analyses

Functional enrichment analyses were performed using Gene Ontology (GO), KEGG pathways, and Reactome pathways to explore potential biological processes, molecular functions, and metabolic or signaling pathways associated with the identified genes. In the Biological Process (BP) category, enrichment analysis suggested a potential overrepresentation of pathways related to secondary metabolism and stress responses. Defense‑related terms, including regulation of defense response (GO:0031347), were among the enriched categories. Metabolism‑associated GO terms, such as sucrose metabolic process (GO:0005985) and cellular carbohydrate metabolic process (GO:0044262), were also detected as enriched, along with ion transport (GO:0006811) and broader biosynthetic processes, including cellular biosynthetic process (GO:0044249) and organic substance biosynthetic process (GO:1901576). Collectively, these enrichments indicate a diverse predicted functional spectrum of the identified hub genes (Figure S14a; Table S5). In the Molecular Function (MF) category, enrichment analysis indicated potential associations with several functional activities. Notably, terpene synthase activity (GO:0010333), magnesium ion binding (GO:0000287), and inorganic phosphate transmembrane transporter activity (GO:0005315) emerged as enriched terms. Additional functions, including beta‑fructofuranosidase activity (GO:0004564) and secondary active transmembrane transporter activity (GO:0015291), were also highlighted, reflecting the predicted functional diversity of the analyzed genes (Figure S14b; Table S6). KEGG pathway enrichment analysis suggested potential involvement of the genes in several metabolic pathways, with carbohydrate‑related routes, such as starch and sucrose metabolism (MAP‑00500) and galactose metabolism (MAP‑00052), appearing among the enriched pathways (Figure S15a; Table S7). Similarly, Reactome pathway enrichment analysis indicated possible enrichment in pathways related to inositol phosphate metabolism (MAP‑1483249) and the synthesis of inositol bisphosphate (IP2), inositol phosphates (IP), and inositol (Ins) in the cytosol (MAP‑1855183). Transport‑associated pathways, including organic anion transporters (MAP‑428643), solute carrier (SLC)‑mediated transmembrane transport (MAP‑425407), and the transport of inorganic cations/anions and amino acids/oligopeptides (MAP‑425393), were also predicted to be represented among the hub genes (Figure S15b; Table S8). Overall, functional enrichment analyses suggest that the identified genes and proteins may be involved in defense‑related processes, metabolic regulation, signaling pathways, and ion transport. These results provide a computational, hypothesis‑generating framework highlighting putative biological functions and pathways that warrant further experimental validation.

### Cross-layer integration of hub genes and hub proteins

To explore potential key molecular players shared between gene‑level and protein‑level regulation, a cross‑layer comparison was conducted between the hub genes identified from the gene co‑expression network and the hub proteins predicted within the PPI network. This integrative analysis was designed to highlight candidate molecules that may occupy central positions at both the transcriptional and post‑translational regulatory levels. The overlap between hub genes and hub proteins suggested the presence of a subset of putative core regulators that repeatedly appeared as central nodes across both network layers. These shared hubs are therefore predicted to represent highly connected elements that may integrate gene expression dynamics with protein interaction architecture. The cross‑layer analysis identified three candidates that were consistently highlighted across both gene‑level and protein‑level network analyses: *TraesCS3A02G417200* (Aldehyde dehydrogenase 1), *TraesCS5D02G542300* (Elongation factor Tu), and *TraesCS2A02G528700* (Double‑strand break repair protein). The recurrent identification of these genes as hubs in both co‑expression and PPI networks provides integrative, prediction‑based evidence suggesting their potential functional importance. These overlapping hubs are predicted to be associated with stress‑related responses and specialized metabolic processes. Based on functional annotation and prior biological knowledge, *TraesCS3A02G417200* (Aldehyde dehydrogenase 1) may contribute to detoxification processes and scavenging of reactive aldehydes, thereby potentially enhancing oxidative stress tolerance under drought conditions. *TraesCS5D02G542300* (Elongation factor Tu) is predicted to be involved in translational processes and protein synthesis, which may support cellular function during stress. Similarly, *TraesCS2A02G528700* (Double‑strand break repair protein) is likely associated with DNA repair pathways and genome stability maintenance, potentially helping to mitigate stress‑induced genomic damage. Collectively, these cross‑layer connections represent hypothesis‑generating candidates whose centrality across multiple network layers suggests possible regulatory relevance. However, these inferences are based on computational network analyses and require targeted experimental validation to confirm their functional roles in drought stress responses.

### Gene/protein relationships across functional gene groups

To explore the potential functional relationships among the candidate genes, gene–gene, protein–protein, and gene/protein networks were generated using the KnetMiner platform. These networks were constructed to predict possible biological associations, co‑regulated nodes, and shared functional pathways that may be relevant to drought tolerance. Based on their putative biological roles, the genes were classified into nine functional groups: (1) DNA replication and repair, (2) signal transduction and kinases, (3) protein degradation via the ubiquitin pathway, (4) ion transporters, (5) transcription factors and translation‑related genes, (6) protein folding and chaperones, (7) secondary or specialized metabolism, (8) enzymes involved in metabolic processes, and (9) hypothetical or uncharacterized proteins. A total of ten networks were generated across these functional categories. The ninth group, comprising hypothetical or uncharacterized proteins, was excluded from network construction due to insufficient functional annotation. From the remaining eight functional groups, ten networks were constructed, with two separate networks generated for each of the enzymes/metabolism (2 networks) and transcription factors (2 networks) groups to accommodate their greater complexity. Collectively, these network‑based representations provide a predictive framework for examining functional connectivity and generating testable hypotheses regarding the roles of these gene groups in drought‑responsive processes.

The gene/protein network associated with DNA replication and repair comprised five genes, among which *TraesCS3A02G097200* (*ETG1*) was connected to a mini‑chromosome maintenance complex‑binding protein. This protein is predicted to exhibit molecular functions related to chromatin binding, suggesting a potential role in genome maintenance processes. The inferred gene–protein interactions were associated with both physiological traits (e.g., grain protein content and oxidative stress response) and phenotypic traits (e.g., root length), based on integrated network and annotation evidence. Notably, *TraesCS3A02G097200* (*ETG1*) was predicted to interact with *TaMYB3R‑2*, a transcription factor previously implicated in regulatory networks controlling protein turnover and stress responses. In the present dataset, this gene was detected under drought conditions without nano‑priming, where it showed an association with SL, while annotation‑based evidence suggested a potential relationship with root length. Collectively, these observations highlight *ETG1* as a putative regulatory component linking DNA replication/repair processes with growth‑related and stress‑responsive traits under drought conditions at seedling stage (Figure S16).

**Fig. 5:**
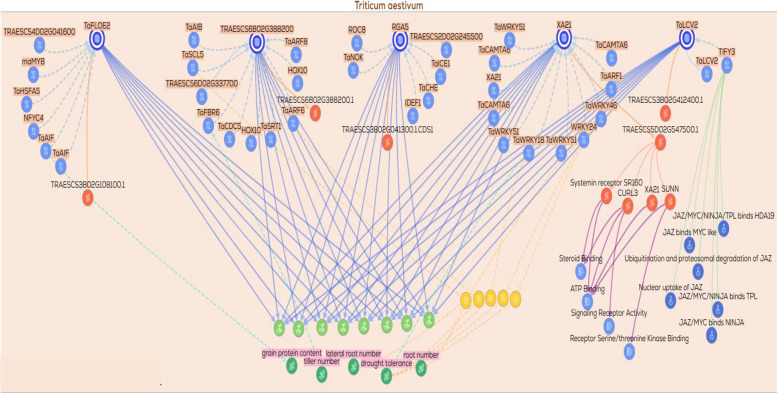
Gene/protein interaction network of signal transduction/kinases related genes

The signal transduction/kinase network comprised five genes, among which *TraesCS5D02G547500* (*XA21*) was predicted to be connected to a non‑specific serine/threonine protein kinase. This protein is inferred to exhibit molecular functions related to ATP binding, signaling receptor activity, and serine/threonine kinase binding, suggesting a potential role in signal transduction processes. The inferred gene/protein nodes were associated, based on network and annotation evidence, with both physiological traits (e.g., grain protein content and drought tolerance) and phenotypic traits (e.g., tiller number, lateral root number, and root number). Notably, *TraesCS5D02G547500* (*XA21*) was predicted to display regulatory interactions with several *WRKY* transcription factors (*TaWRKY51*, *TaWRKY18*, *TaWRKY46*, and *TaWRKY24*), which have been previously implicated in stress signaling and drought‑response pathways. In the present dataset, this gene was associated with RNo under drought conditions with nano‑priming, while concordant annotation‑based evidence suggested potential links to additional root‑related traits (lateral root number and root length) and physiological attributes (drought tolerance, transpiration rate, and chlorophyll content). Collectively, these observations highlight *XA21* as a putative regulatory component involved in signal transduction networks that may participate in root system modulation under drought stress, warranting further experimental validation (Fig. 5).

**Fig. 6:**
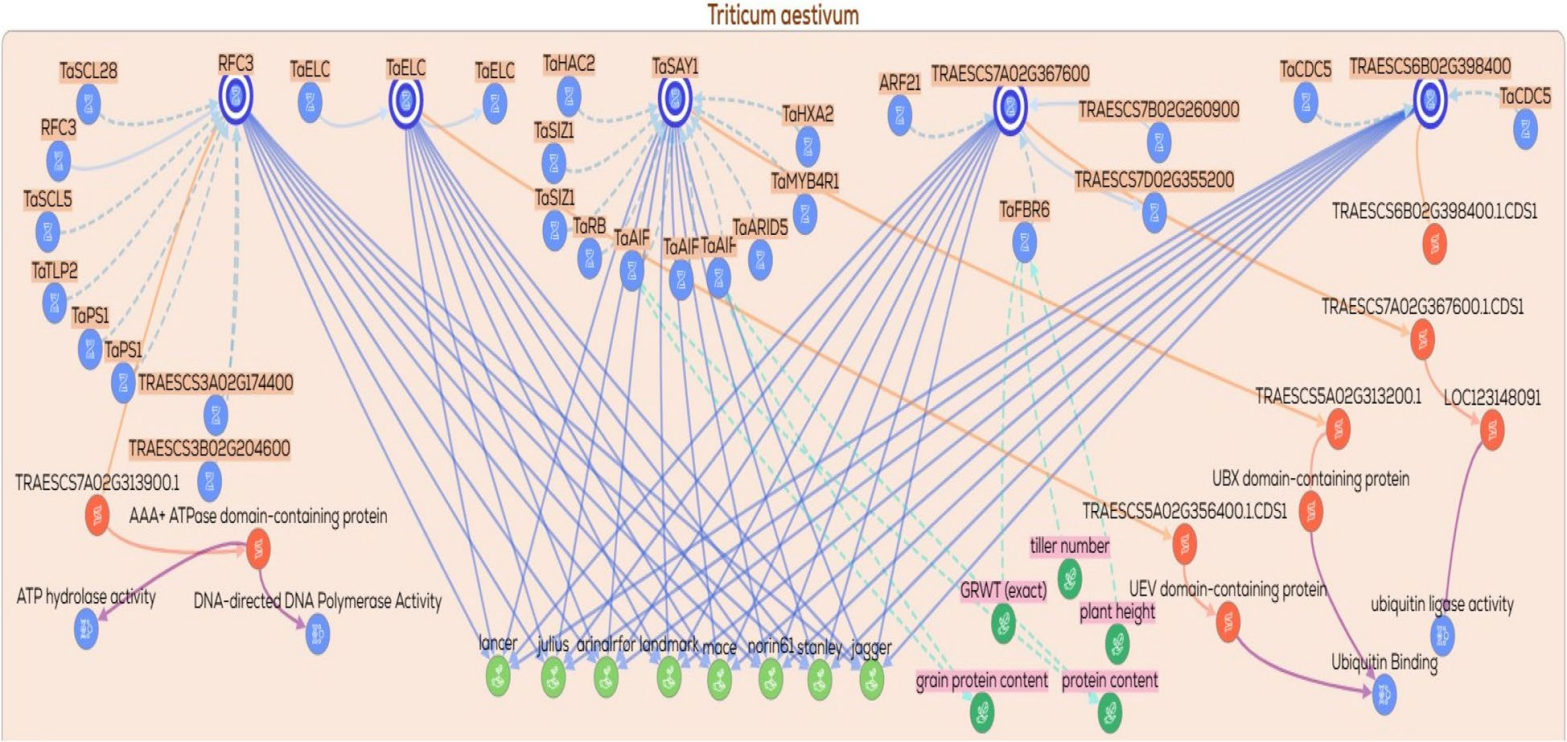
Gene/protein interaction network of protein degradation/ubiquitin pathway related genes

The third network corresponds to the protein degradation/ubiquitin pathway. This category comprises five genes predicted to be involved in ubiquitin‑mediated protein degradation (Fig. 6). Among these, *TraesCS5A02G313200* (*SAY1*) was predicted to exhibit regulatory interactions with *TaMYBR1*, a transcription factor previously implicated in regulatory networks controlling protein turnover and stress responses. In the present dataset, *SAY1* was detected under control conditions with nano‑priming, where it showed an association with SL, while annotation‑based evidence suggested a potential link to plant height. Moreover, the inferred gene/protein nodes were associated, based on integrated network and annotation evidence, with both phenotypic traits (e.g., tiller number and plant height) and physiological traits (e.g., protein content and grain protein content). Collectively, these observations highlight *SAY1* as a putative regulatory component of the ubiquitin‑mediated proteostasis pathway that may contribute to growth‑ and stress‑related trait variation, warranting further experimental validation.

**Fig. 7:**
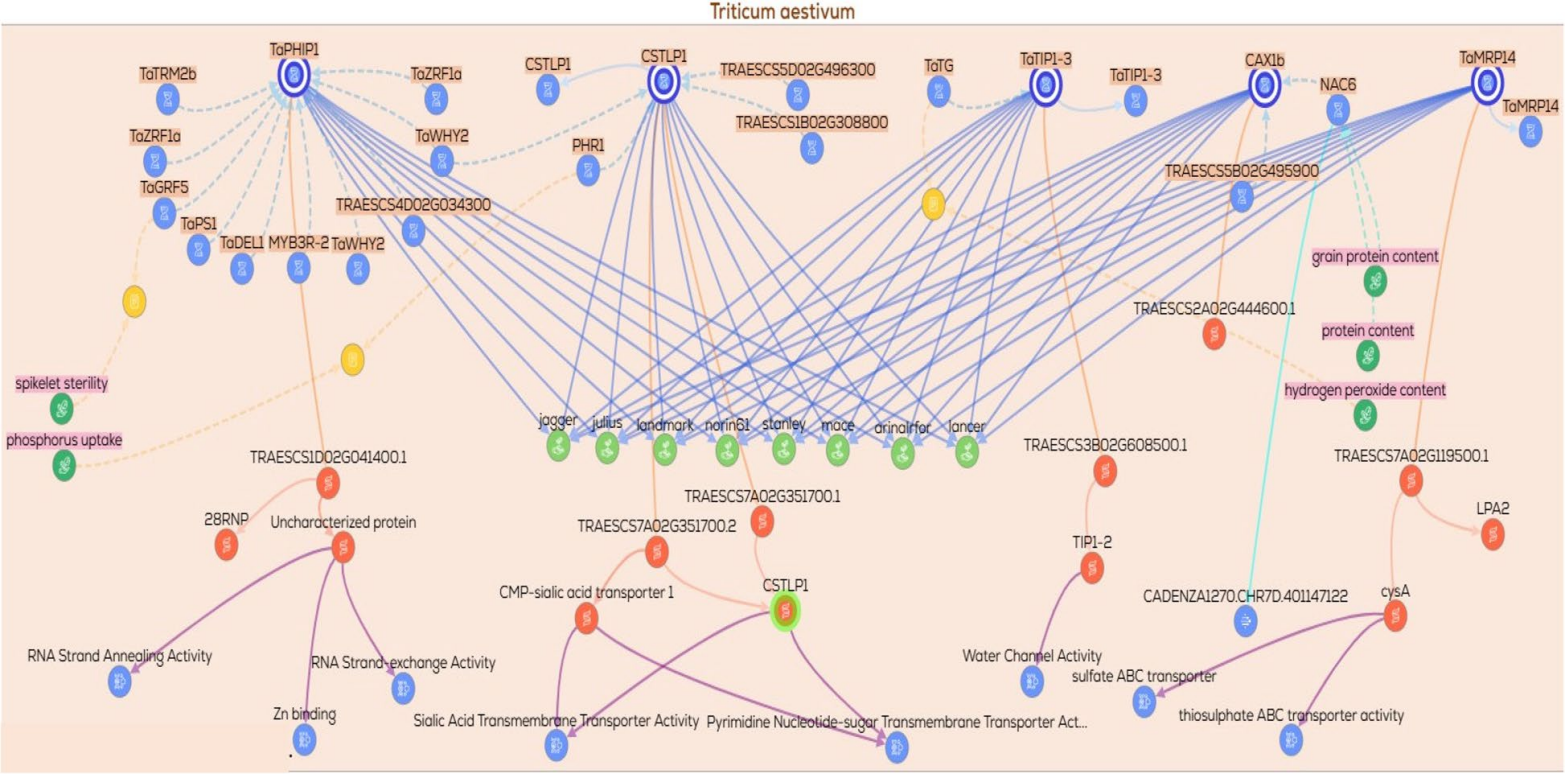
Gene/protein interaction network of ion transporter related genes

The ion transporter network comprised genes such as *TraesCS2A02G444600* (*CAX1b*), *TraesCS3B02G608500* (*TIP1‑3*), and *TraesCS1D02G041400* (*PHIP1*), which were predicted to be connected to proteins including a vacuolar cation/proton exchanger, a tonoplast intrinsic protein, and an uncharacterized protein. Collectively, these proteins are inferred to exhibit diverse molecular functions, including calcium/proton antiporter activity, water channel activity, and zinc binding as well as RNA strand annealing and exchange activity. Based on integrated network and annotation evidence, the inferred gene-protein nodes were associated with several physiological traits, including hydrogen peroxide content, protein content, and grain protein content, in addition to developmental and reproductive traits such as phosphorus uptake and spikelet sterility. Notably, *PHIP1* was annotated with zinc‑binding activity and, in the present dataset, was detected specifically under drought conditions with ZnO-NPs nano‑priming, suggesting a potential role in zinc‑mediated stress responses. Furthermore, *PHIP1* was predicted to exhibit regulatory interactions with *MYB3R‑2*, while *CAX1b* showed inferred regulatory connections with members of the *NAC* transcription factor family, which is widely implicated in drought‑stress signaling. In this study, both genes were associated with root‑related traits, including RRL and RNo, under drought conditions with nano‑priming. Collectively, these observations suggest a potential functional relevance of these ion‑transporter‑related genes in the modulation of adaptive root system architecture under water‑deficit conditions (Fig. 7).

**Fig. 8:**
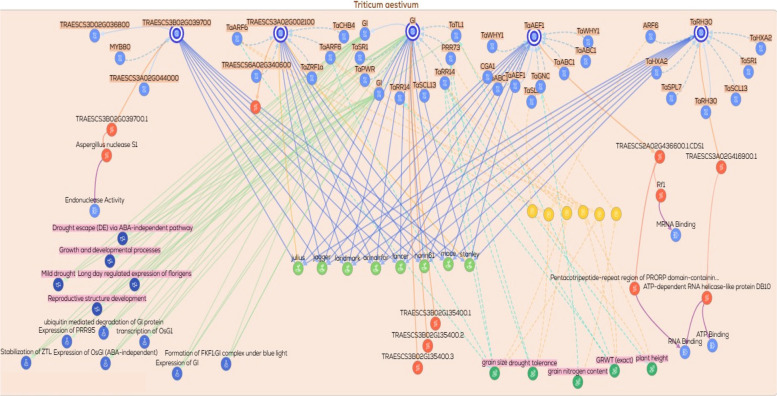
Gene/protein interaction network of transcription factors related genes

The transcription factor networks were divided into two sub‑networks to highlight their distinct functional associations. The first sub‑network comprised transcription factors predicted to be linked to translation, protein synthesis, photosynthesis, and chloroplast‑related processes. This sub‑network included five genes, among which *TraesCS6B02G408900* (encoding response regulators 29; *RR29*) and *TraesCS2B02G105100* (encoding heat shock factor A2-5; *HSFA2‑5*) were inferred to be connected to proteins such as response regulatory domain‑containing proteins, HTH MYB‑type domain‑containing proteins, and *HSFA2h*. Collectively, these proteins are predicted to exhibit molecular functions related to DNA‑binding transcription factor activity. Based on integrated network and annotation evidence, the inferred gene-protein nodes were associated with several phenotypic and physiological traits, including seed coat color, glume color, germination rate, and carotenoid content. Notably, *HSFA2‑5* was predicted to exhibit regulatory interactions with *TaDREB2*, a drought‑responsive element‑binding factor widely implicated in stress‑signaling pathways. In the present dataset, this gene was consistently detected under drought conditions with nano‑priming, where it showed an association with root number. Similarly, *RR29* displayed inferred regulatory connections with *TaNAC044* and, in this study, was detected under control conditions with nano‑priming, where it was associated with SL (Figure S17). The second sub‑network comprised transcription factors predicted to be involved in RNA‑processing‑related pathways. This group included *TraesCS3B02G039700*, which was inferred to connect to an Aspergillus nuclease S1‑like protein exhibiting endonuclease activity. The inferred gene-protein nodes were associated, based on network and annotation evidence, with agronomic and physiological traits such as drought tolerance, grain size, grain nitrogen content, and plant height. Pathway analysis further suggested potential involvement of this sub‑network in drought escape through ABA‑independent signaling, mild drought responses, and long‑day‑regulated expression of florigens. Within this network, *TraesCS3B02G039700* was predicted to exhibit regulatory interactions with *MYB80*. In the present dataset, this gene was consistently detected under drought conditions with nano‑priming and was associated with SVI (Fig. 8).

The protein folding and chaperone–cytoskeleton/cell division network comprised five genes, among which *TraesCS7A02G235100* was predicted to be connected to a J‑domain‑containing protein. Functional annotation suggested potential molecular functions, including metal binding, indicating a possible role in protein folding and cellular stress responses. Based on integrated network and annotation evidence, the inferred gene-protein nodes were associated with several agronomic and physiological traits, including drought tolerance, plant height, grain size, and root length. Notably, *TraesCS7A02G235100* was predicted to exhibit regulatory interactions with MADS-box transcription factor 51 (*MADS51)*, a member of the MADS‑boxfamily that has been widely implicated in environmental adaptation. In the present dataset, this gene was consistently detected under drought conditions with nano‑priming and showed an association with CVt. Collectively, these observations suggest that *TraesCS7A02G235100* may represent a putative regulatory component linking chaperone‑mediated proteostasis and developmental responses under drought stress, warranting further experimental validation (Figure S18). 

The secondary metabolism network comprised the genes *TraesCS6B02G091400* and *TraesCS6B02G091000* (both encoding *Triticum aestivum* trehalose-6-phosphate synthase 14; *TaTPS14)*, which were predicted to be connected to proteins such as linalool synthase and terpene synthase containing metal‑binding domains. Functional annotation suggested potential molecular functions, including terpene synthase activity and magnesium ion binding, indicating a possible role in specialized metabolic pathways. Pathway analysis further indicated potential involvement of these genes in auxin signaling, hormone‑related signaling pathways, metabolite transport and regulation, and carotenoid biosynthesis. Based on integrated network and annotation evidence, the inferred gene-protein nodes were associated with several agronomic and physiological traits, including drought tolerance, plant height, root length, grain protein content, and chlorophyll content. Notably, *TraesCS6B02G091400* (*TaTPS14*) was predicted to exhibit regulatory interactions with *TaNAC011*. Similarly, *TraesCS6B02G091000* (*TaTPS14*) showed inferred regulatory connections with multiple *WRKY* transcription factors (*TaWRKY11*, *TaWRKY18*, *TaWRKY24*, *TaWRKY27*, *TaWRKY33*, *TaWRKY46*, and *TaWRKY51*) and additionally displayed putative associations with *MYB* transcription factors. In the present dataset, both genes were consistently associated with SL under control conditions with nano‑priming. Collectively, these observations suggest that *TaTPS14*‑related genes may represent putative components linking secondary metabolism, hormonal regulation, and growth‑related traits under nano‑priming conditions (Figure S19).

**Fig. 9:**
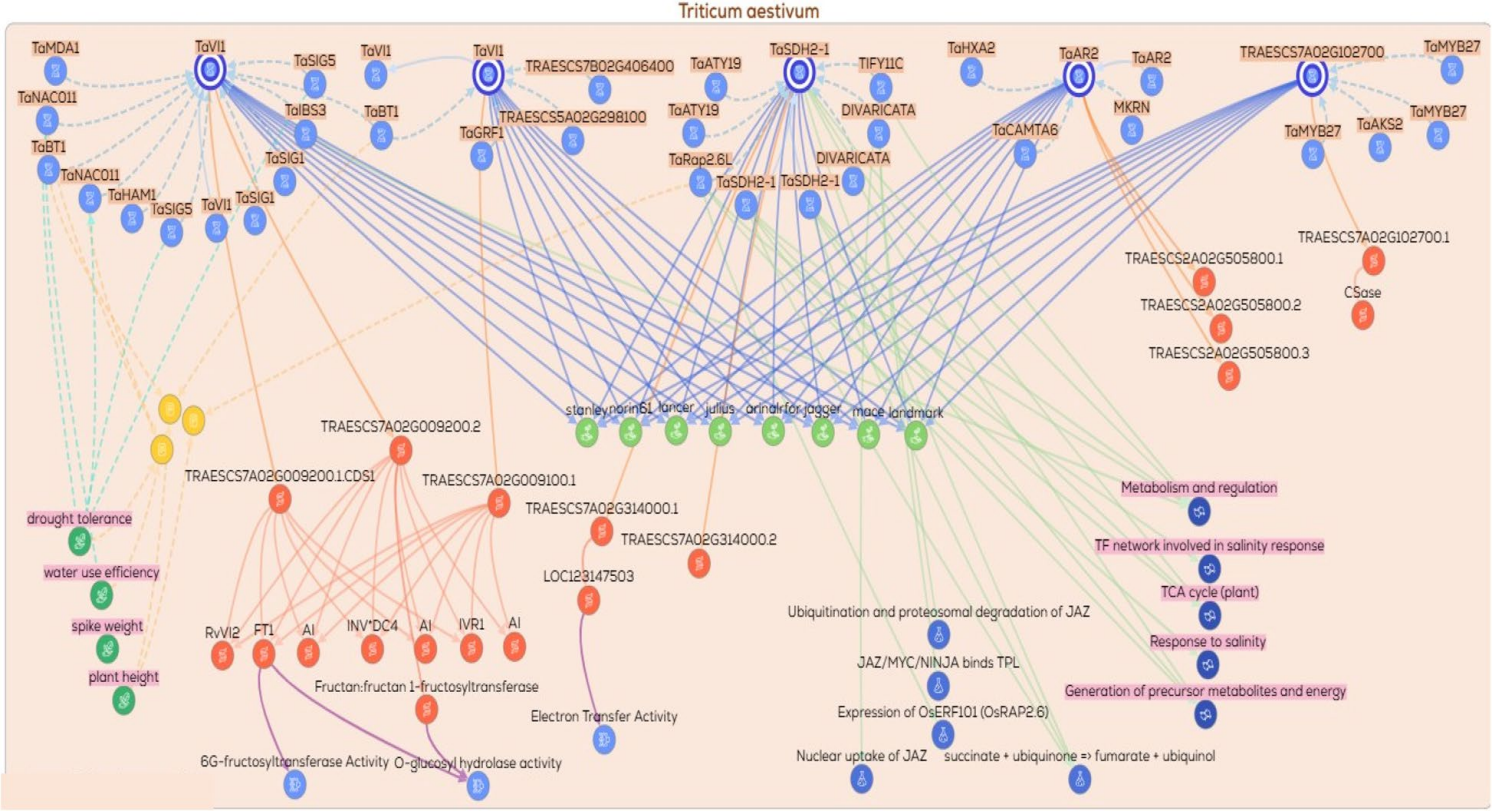
Gene/protein interaction network 1 of enzymes/metabolism related genes

The enzymes/metabolism subnetwork I comprised the genes *TraesCS7A02G102700*, *TraesCS7A02G314000* (*TaSDH2-1*), and *TraesCS7A02G009200* (*TaVI2*), which were predicted to connect to proteins such as NADPH–cytochrome P450 reductase, tryptophan synthase, succinate dehydrogenase, and glycosyl hydrolase. Functional annotation indicated potential molecular functions, including oxidoreductase activity, transferase activity, hydrolase activity, and electron carrier activity. According to network‑based inference, the gene/protein nodes were associated with agronomic and physiological traits such as drought tolerance, water‑use efficiency, spike weight, and plant height. Notably, *TraesCS7A02G009200* (*TaVI2*) and *TraesCS7A02G314000* (*TaSDH2-1*) were predicted to exhibit regulatory interactions with *TaNAC011* and *DIVARICATA*, respectively, and were consistently detected under drought conditions with nano‑priming, where they were associated with RL and Z traits. In contrast, *TraesCS7A02G102700* was inferred to display regulatory interactions with *MYB27* and was specifically detected under drought conditions without nano‑priming, suggesting a potential link between MYB‑mediated transcriptional regulation, growth suppression, and metabolic adjustment under water‑deficit conditions (Fig. 9). The enzymes/metabolism subnetwork II comprised the genes *TraesCS5A02G003800* (*HIPL2*) and *TraesCS3A02G212300* (*REF1*), which were predicted to be connected to proteins such as glucose/sorbosone dehydrogenase and aldehyde dehydrogenase 1. Functional annotation suggested potential molecular functions, including redox activity and dehydrogenase activity, indicating possible roles in maintaining cellular redox balance, detoxification, and energy‑linked metabolic fluxes. Based on integrated network and annotation evidence, the inferred gene-protein nodes were associated with several agronomic and physiological traits, including leaf rolling, drought tolerance, oxidative stress response, and starch content. Notably, *HIPL2* was predicted to exhibit regulatory interactions with *MYB31*. In the present dataset, this gene was detected under both drought and control conditions without nano‑priming and showed an association with RNo, suggesting a potential role in root system architecture and adaptive resilience. In addition, *REF1* displayed inferred regulatory interactions with several transcription factors, including *MYB93*, *MYB80*, *MYB2*, *MAD27*, and *MYC2*. In the present dataset, *REF1* was specifically detected under drought conditions and was associated with RFW, based on integrated network and trait annotations (Figure S20).

## Discussion

### Phenotypic variation and analysis of variance

Drought stress markedly affected seed germination and early seedling traits, reflecting the well‑documented adverse effects of water deficit on metabolic processes, membrane integrity, and cell expansion [49]. These findings are consistent with previous studies demonstrating that drought stress disrupts physiological homeostasis during early growth stages. In contrast, nano‑priming with zinc oxide nanoparticles (ZnO-NPs) substantially alleviated the negative effects of drought stress, supporting earlier reports that ZnO-NP priming enhances germination performance, seedling vigor, and antioxidant capacity under water‑limited conditions [50, 51]. The near‑normal distribution observed for most germination and seedling traits indicates the quantitative, polygenic nature of drought‑responsive traits, which is a common feature reported in wheat QTL studies conducted under PEG‑induced drought stress (Figure S1–S3) [35]. Deviations from normality detected for certain traits under both control and drought conditions may reflect ceiling effects in optimal environments or differential genotype sensitivity under stress, phenomena frequently associated with plant stress responses [49, 52].

Analysis of variance (ANOVA) revealed highly significant genetic variation among genotypes across all treatments, confirming that germination and seedling traits under both control and drought conditions are strongly genotype‑dependent (Tables 3, 4 and 5). Similar pronounced genotypic effects under PEG‑induced drought stress have been reported in earlier wheat seedling studies, underscoring the genetic control of early‑stage drought responses [53]. Broad‑sense heritability estimates were high for all evaluated traits, with consistently higher values under control compared with drought conditions. This pattern is expected, as environmental variance typically increases under stress, thereby reducing heritability estimates. Comparable trends have been observed in wheat, where heritability of growth and physiological traits generally declines under drought relative to well‑watered conditions [54]. Overall, the combination of strong genotypic effects, substantial phenotypic variation, and consistently high heritability values supports the reliability and genetic tractability of the investigated germination and seedling traits. These characteristics demonstrate their suitability for QTL identification and genetic dissection of drought tolerance during early developmental stages, providing a strong foundation for downstream mapping and breeding applications [55].

### QTL mapping

Drought tolerance is a complex quantitative trait controlled by multiple genes, and QTL analysis represents a powerful approach for uncovering its underlying genetic architecture [56]. This strategy has been widely and effectively applied to identify QTLs and molecular markers associated with drought tolerance during early developmental stages in major cereal crops, including wheat, rice, barley, and maize, underscoring its broad utility in the genetic dissection of abiotic stress responses [57].

ICIM was applied to 22 germination and seedling traits under four treatments using 98-genotypes subset, we acknowledge that this falls at the lower end of the recommended range for QTL detection (100–500 individuals) [58]. While this limitation might affect the overall statistical power to detect rare or minor-effect loci, high heritability estimates and a dense SNP marker set (3,457 markers) strengthen the reliability of major QTLs identified in this study. Furthermore, the use of a high‑density marker set substantially improves mapping accuracy and helps offset some of the limitations associated with smaller population sizes, ensuring that the identified major-effect QTLs provide robust candidates for future functional analysis. These findings provide a solid foundation for the prioritized candidate loci; however, further validation across larger and more diverse populations is recommended to confirm the consistency of these loci. Additionally, functional studies such as CRISPR-Cas9 or gene knockout will be instrumental in definitively characterizing the molecular mechanisms of these candidates in wheat under drought conditions.

Moreover, several significant markers identified in the present study exhibited strong concordance with previously reported loci associated with drought tolerance and related agronomic traits in wheat, reinforcing the robustness and biological relevance of our findings. For example, the marker *Ra_c16330_1197*, which was associated in this study with SL, SVI, FWDTI, and RFW under control and drought conditions without nano‑priming, has previously been linked to drought tolerance in a panel of 299 winter wheat lines [59]. Similarly, *wsnp_Ex_c11827_18986376*, which was associated here with IGP, GP, MGR, and MGT under drought conditions with nano‑priming, has also been reported in association with grain yield components in a panel of 198 bread wheat genotypes [60], productive spikelet rate in 192 wheat lines [61], and calcium micronutrient concentration in 105 diverse wheat accessions [62]. The marker *IAAV3924*, associated with SVI under drought with nano‑priming in the present study, had likewise been reported in 400 winter wheat recombinant inbred lines for its association with normalized difference vegetation index (NDVI) [63]. A similar pattern was observed for *BS00024829_51*, which was associated here with SRR under drought with nano‑priming and previously linked to NDVI and plant height in the same winter wheat population [63]. Additionally, *TA004785_1734*, identified here in association with U under drought with nano‑priming, had been reported in 92 single chromosome recombinant doubled haploid lines for its association with the stem length tolerance index (IT_StL) [64]. Likewise, *RAC875_c40654_206*, associated in this study with RRL under drought without nano‑priming, had previously been linked to seed maturity date in spring wheat evaluated across irrigated and rainfed environments in northern Kazakhstan [65]. Furthermore, *RAC875_c47550_437*, which was associated here with RLDTI and RRL, has been reported in association with grain yield and grains m⁻^2^ in 400 winter wheat recombinant inbred lines [63]. Finally, *Kukri_c33670_261*, associated in this study with RRL under drought conditions, was previously identified in a population of 149 hard red spring wheat recombinant inbred lines for its association with tiller number [66].

In this study, a total of 51 QTLs associated with germination and seedling traits were identified across all experimental treatments. Major QTLs with high phenotypic variance explained (PVE) were predominantly distributed across the A and B genomes. Specifically, QTLs were detected on chromosomes 2A (8), 3A (5), 4A (7), 5A (8), and 7A (8), in addition to chromosomes 2B (1), 3B (4), 5B (2), 6B (2), and 7B (4), while fewer loci were identified on the D genome, including chromosomes 1D (1) and 5D (1). Collectively, these QTLs were linked to a broad range of germination and seedling‑related traits (Table 6; Fig. 3).

The QTLs discussed below are compared with previously reported genomic regions based on chromosomal co-localization rather than direct correspondence. While these regions have been associated with various agronomic, yield-related, or stress-responsive traits in previous studies, they were primarily identified at different developmental stages, such as the vegetative or reproductive phases. In contrast, the QTLs identified in the present study represent newly detected loci specifically for germination, seedling development, and early-stage drought response under the studied conditions. Accordingly, these overlapping QTLs are considered putative and highlight genomic regions of interest that merit further investigation through fine mapping and independent validation to confirm their stage-specific effects. As highlighted in recent discussions on genetic validation [41], such validation steps are essential to bridge the gap between basic QTL discovery and their practical application in crop improvement, ensuring that these genomic regions are robust enough for breeding programs.

Eleven QTLs associated with control conditions, with and without nano‑priming, were mapped to chromosomes 4A (2), 5A (5), 5B (1), 6B (1), and 7B (2) and were linked the traits SL, RNo, IG %, SVI, U, Z, and CVt (Table 6).

On chromosome 4A, two QTLs (*QSL_C4A* and *QSVI_C4A*) were detected under control conditions without nano‑priming and were associated with SL and SVI, respectively, indicating a pleiotropic effect. These QTLs were mapped to the same genomic interval at 111.0 cM (confidence interval: 107.5–112.5 cM). Notably, this interval overlaps with chromosomal regions previously reported to harbor QTLs for several yield‑related traits, including kernel length (104.9–112.0 cM) [67], spike length (111.2–117.8 cM), spike number per spike (110.8–111.2 cM) [68], fertile spike number per spike (108.0 cM) [69], sterile spike number per spike (105.7–110.5 cM) [70], and grain yield (111.2–115.4 cM) [71]. The overlap between early seedling vigor traits and yield‑related components suggests a potential genetic linkage between early growth performance and later productivity.

In addition, a QTL for shoot length (*QSL_CN5A*) detected under nano‑priming was mapped at 118.0 cM, proximal to genomic regions reported for kernel length (105.5–116.5 cM) [63], sterile spikelets (107.1–120.3 cM) [66], and grain width (117.0 cM) [68]. Furthermore, another QTL for shoot length (*QSL_CN6B*) under nano‑priming was identified on chromosome 6B at 95.0 cM, falling within regions previously linked to plant height (95.48–97.05 cM) [69] and near loci associated with grain zinc content (~ 100–106 cM) [70, 71]. Collectively, these overlapping intervals indicate shared genomic regions potentially influencing early vigor, plant architecture, and micronutrient‑related traits.

On chromosome 5A, two QTLs (*QU_C5A* and *QCVt_C5A*) were detected under control conditions without nano‑priming and were associated with U and CVt, respectively. These QTLs were mapped to a common genomic interval at 133.0 cM (CI: 131.5–133.5 cM). Notably, this region overlaps with chromosomal intervals previously reported to harbor QTLs for tiller number (131.9–153.2 cM) [69]. This overlap points to a possible genetic relationship between early seedling establishment and tillering capacity. In addition, a QTL for SL (*QSL_CN5A*) under nano‑priming was mapped to 118.0 cM, proximal to regions previously reported for kernel length (105.5–116.5 cM) [67], sterile spikelets (107.1–120.3 cM) [70], and grain width (117.0 cM) [72]. Furthermore, a QTL for SL (*QSL_CN6B*) under nano‑priming was identified on chromosome 6B at 95.0 cM, coinciding with previously reported QTLs for plant height (95.48–97.05 cM) [73] and located near loci associated with grain zinc content (~ 100–106 cM) [74, 75]. Collectively, these overlapping intervals indicate shared genomic regions potentially influencing early vigor, plant architecture, and micronutrient‑related traits.

Twenty‑six QTLs associated with drought conditions, with and without nano‑priming, were identified across multiple chromosomes, including 2A (6), 3A (1), 4A (1), 5A (3), 7A (6), 2B (1), 3B (3), 5B (1), 6B (1), 7B (2), and 5D (1), and collectively spanned all evaluated germination and seedling traits (Table 6).

On chromosome 2A, four QTLs (*QIG %_DN2A*, *QGP_DN2A*, *QMGR_DN2A*, and *QMGT_DN2A*) were detected under drought conditions with nano‑priming and were associated with IG %, GP, MGR, and MGT, respectively, indicating a pleiotropic effect. These QTLs were mapped to a shared genomic interval ranging from 34 to 36 cM (CI: 24.5–45.5 cM). Notably, this region overlaps with chromosomal intervals previously reported to harbor QTLs for plant height (30 cM) [76], grain filling duration (33.72–39.7 cM) [77], grain yield (30.92 cM) [78], cell membrane stability (27.1 cM) [79], grains per spike (38.08 cM) [80], and grain zinc content (30–38.5 cM) [81]. In addition, two QTLs (*QU_DN2A1* and *QU_DN2A2*) associated with U were identified on chromosome 2A under drought conditions with nano‑priming. *QU_DN2A1*, mapped at 47 cM (CI: 46.5–48.4 cM), co‑localized with regions previously associated with root depth (47 cM) [82], root architecture (46.57–47.9 cM) [83], flag leaf greenness (46.5 cM) [84], heading date and spike number per unit area (47.55 cM) [80], as well as grain zinc content (46.1–48.4 cM) [85]. The second QTL (*QU_DN2A2*), mapped at 61 cM (CI: 52.5–61.5 cM), lies within a well‑established zinc‑associated hotspot overlapping QTLs for grain zinc concentration at 60–64.1 cM [74, 86]. This interval has also been linked to days to maturity (55.95–65.26 cM) and plant height (65.02 cM) [77, 80], indicating multi‑trait relevance. Nevertheless, the strong concordance with zinc‑specific loci suggests that zinc accumulation may represent the primary functional driver within this region. Altogether, chromosome 2A represents a major hotspot potentially coordinating germination vigor, root traits, phenology, and zinc nutrition under drought conditions with nano‑priming.

On chromosome 3A, a single QTL (*QRNo_D3A*) was identified under drought conditions without nano‑priming and was associated with RNo. This QTL was mapped to a narrow genomic interval at 1.0 cM (CI: 0–1.5 cM). Notably, this region overlaps with chromosomal intervals previously reported to harbor QTLs for root hair length (0–6 cM) [87], days to heading (0–2.5 cM) [88], grain number per spike (0–3 cM) [89], spikelet number (0–2.5 cM) [88], and spike length (0.9–1.8 cM) [90]. The overlap between QTLs for root number and root hair length implies a potential role of this region in regulating multiple root‑related traits during early growth, highlighting its potential role in drought adaptation.

On chromosome 4A, a single QTL (*QFW_DN4A*) was identified under drought conditions with nano‑priming and was associated with FW. This QTL was mapped to 56 cM (CI: 53.5–56.5 cM). Notably, this genomic interval overlaps with chromosomal regions previously reported to harbor QTLs for drought tolerance (55.5 cM) [91], grain zinc content (59.2 cM) [92], and days to maturity (51–56 cM) [93]. The co-localization with a zinc-associated interval is noteworthy, as 4A is a key chromosomal segment for Zn accumulation and homeostasis. The proximity of *QFW_DN4A* to a Zn-related locus suggests this region may influence early biomass production alongside molecular pathways regulating zinc uptake and allocation, making it a promising target for integrated breeding strategies.

On chromosome 5A, three QTLs (*QRNo_D5A*, *QGP_D5A*, and *QU_D5A*) were identified under drought conditions without nano‑priming and were associated with RNo, GP, and U, respectively. *QRNo_D5A*, mapped at 3 cM (CI: 1.5–17.5 cM), co‑localized with chromosomal regions previously reported to harbor QTLs for abscisic acid accumulation (13.5 cM) [94], flag leaf traits (4 cM) [84], heading date (15 cM) [95], kernel length (8 cM) [96], plant height (6.4 cM) [97], and carbohydrate remobilization (10.5 cM) [91], suggesting pleiotropic regulation of early stage root development and later vegetative/reproductive traits. In addition, *QGP_D5A* and *QU_D5A* were mapped to a shared genomic interval at 127 cM (CI: 118.5–139.5 cM), coinciding with previously reported QTLs for root depth (123.6 cM) [83], grain yield (126 cM) [98], heading date (129.62 cM) [80], plant height (127.2–155.8 cM) [99], and thousand‑grain weight (127.8–130 cM) [98]. These overlaps indicate that chromosome 5A harbors key regulatory loci coordinating early vigor, developmental timing, and yield components, reinforcing its importance for integrated breeding strategies.

Chromosome 7A harbored six QTLs, including three identified under drought conditions without nano‑priming (*QGP_D7A*, *QMGR_D7A*, and *QMGT_D7A*) and three detected under drought conditions with nano‑priming (*QRL_DN7A*, *QZ_DN7A*, and *QCVt_DN7A*). These QTLs were associated with GP, MGR, MGT, RL, Z, and CVt. A pleiotropic QTL cluster located at approximately 62–63 cM (CI: 49.5–76.5 cM) was associated with GP, MGR, and MGT. Notably, this interval overlaps with chromosomal regions previously reported to harbor drought‑responsive QTLs for heading date (54.3–74.9 cM) [100], root architecture (63.74–64.27 cM) [82], grain yield (68.73 cM) [101], spike number per unit area (63.32 cM) [80], and plant height (60.45–63.45 cM) [102]. Although these loci were identified for different traits and developmental stages, their co‑localization with germination‑timing QTLs suggests that this region may contain regulatory factors influencing developmental timing under drought stress. This may account for the recurrent detection of this interval across both early germination traits and heading‑date‑related phenotypes. In addition, *QZ_DN7A*, mapped at 123 cM (CI: 121.5–123.5 cM), overlapped with genomic regions previously associated with thousand‑grain weight (121.1–126.9 cM) [103] and spike length (119.4–130.7 cM) [104]. This overlap suggests a potential genetic linkage between early germination performance and later yield‑related traits under drought conditions.

On chromosome 5B, a single QTL (*QSRR_DN5B*) was identified under drought conditions with nano‑priming and was associated with SRR. This QTL was mapped to 195 cM (CI: 191.5–196.5 cM). Notably, this genomic interval overlaps with chromosomal regions previously reported to harbor QTLs for plant height (193–195 cM) [105], indicating a shared regulatory basis for shoot elongation. Since SRR depends on shoot length, its presence in both early (SRR) and late (plant height) stages suggest this locus coordinates above-ground biomass allocation across developmental phases, making it a key target for improving growth under stress.

On chromosome 7B, two QTLs (*QGP_DN7B* and *QMGR_DN7B*) were identified under drought conditions with nano‑priming and were associated with GP and MGR, respectively. These QTLs were mapped to a common genomic interval at 50 cM (CI: 46.5–50.5 cM), indicating a pleiotropic effect. Notably, this interval overlaps with chromosomal regions previously reported to harbor QTLs for grain zinc content (44 cM) [106], thousand‑grain weight (44.4–52.1 cM) [107], spike number per plant (50.1–60.9 cM) [108], grain yield (49.8–77.1 cM) [109], and harvest index (44–52 cM) [110]. The proximity to a grain Zn locus suggests a zinc-centered regulatory interval: ZnO nano-priming may enhance zinc-linked signaling and transport during germination, while the same region contributes to Zn accumulation in grain later, reflecting pleiotropy across developmental stages.

Fourteen QTLs associated with drought tolerance indices and reduction‑related traits, under both nano‑primed and non‑primed drought conditions, were mapped to chromosomes 2A (2), 3A (4), 4A (4), 7A (2), 3B (1), and 1D (1), collectively encompassing all evaluated traits (Table 6). On chromosome 4A, two QTLs for fresh weight reduction (*QRFW_D4A1* and *QRFW_D4A2*) were mapped at 105 cM (CI: 104.5–105.5 cM) and 111 cM (CI: 108.5–112.5 cM), respectively. Notably, these genomic intervals overlap with chromosomal regions previously reported to harbor QTLs for drought and heat tolerance (67.97–108.75 cM) [110], grain number per spike (101.6–107.4 cM) [111], grain yield (105 cM) [98], grain weight per spike (103.7–106.5 cM) [112], spike length (111.2–117.8 cM) [68], spikelet number (110.8–111.2 cM) [68], and kernel weight per spike (104.9–125.7 cM) [67]. This co-localization suggests that the 4A region regulates biomass conservation and growth under drought, reinforcing its role as a key drought-adaptive genomic hotspot.

### Gene annotation, expression and mapping of the candidate genes

Functional annotation of genes located within the identified QTL intervals suggests a multilayered regulatory framework potentially underlying drought tolerance during germination and early seedling development. The enrichment of genes related to DNA repair and replication may indicate mechanisms involved in maintaining genome stability under dehydration‑induced oxidative stress [113]. Similarly, the presence of kinase‑mediated signaling components highlights phosphorylation‑based regulatory cascades that are likely to participate in coordinating transcriptional and metabolic responses to water deficit [113]. Post‑translational regulation mediated by the ubiquitin–proteasome system further suggests selective protein turnover as a potential adaptive mechanism under stress conditions, consistent with established roles of ubiquitination and SUMOylation in abiotic stress responses [114, 115]. In addition, ion transport‑related genes, including cation/proton antiporters and calcium‑related transporters, may contribute to osmotic adjustment and calcium‑dependent signaling processes under drought stress [116, 117]. The identification of transcription factors and molecular chaperones supports the notion of transcriptional reprogramming and protein stabilization as key components of stress adaptation during early growth stages [49]. Notably, the enrichment of zinc‑associated genes aligns with previous reports highlighting the involvement of zinc in antioxidant defense, osmoprotection, and early seedling vigor. This observation further supports the hypothesis that ZnO nano‑priming may enhance drought tolerance by modulating zinc‑related physiological and molecular pathways during germination and early seedling growth [51, 118]. Overall, drought tolerance at the germination stage represents a complex quantitative trait governed by polygenic control and requires the coordinated modulation of stress sensing, signal transduction, proteostasis, ion homeostasis, and metabolic adjustment pathways. The convergence of these biological functions within the identified QTL regions reinforces the biological plausibility of the prioritized candidate genes and provides a robust, hypothesis‑generating framework for subsequent functional validation and targeted breeding efforts.

The identification of three distinct expression profiles, upregulated, downregulated, and stably expressed genes, is consistent with previous transcriptomic analyses of wheat responses to drought stress (Fig. 4). The upregulated group, comprising both stress‑enhanced and strictly inducible genes, corresponds to prior RNA‑seq and RT‑qPCR evidence demonstrating the rapid activation of signaling cascades, osmotic adjustment mechanisms, and antioxidant defenses under PEG‑induced drought conditions, with stronger induction typically observed in tolerant genotypes [119–121]. In addition, several candidate loci anchored within QTL regions have been reported to exhibit drought‑responsive induction, including genes encoding transcription factors and stress‑related proteins such as GATA-binding factors (GATA), U‑box domain-containing proteins (U-box), and glutathione S‑transferases (GSTs) [122]. Conversely, downregulated genes largely reflect the repression of growth‑ and energy‑demanding processes, including translation and starch biosynthesis, which is a hallmark of dehydration stress, particularly in drought‑sensitive genetic backgrounds [123, 124]. Genes exhibiting stable expression across control and drought conditions likely represent constitutive defense and homeostasis modules, including molecular chaperones, antioxidant systems, and transporters that help maintain cellular integrity under stress. Notably, tolerant cultivars often retain the expression of these baseline regulatory layers more effectively than susceptible lines [120, 125]. Collectively, these expression patterns support a regulatory model in which inducible stress‑response modules (encompassing signaling, osmoprotection, and antioxidant pathways) are selectively upregulated, growth‑associated metabolic programs are attenuated, and constitutive homeostatic mechanisms are preserved. This coordinated response has been repeatedly linked to drought resilience in wheat and other cereals and offers a rational basis for prioritizing candidate genes for functional characterization and breeding‑oriented validation [119, 121, 125].

### Network analyses

#### Gene prioritization (Gene network direct neighborhood)

By projecting curated guide genes onto the WheatNet platform and prioritizing direct‑neighborhood clusters, drought‑responsive candidate genes were found to aggregate into functionally coherent modules rather than appearing as isolated network nodes. Twelve prioritized gene groups converged on biological processes that are central to dehydration stress responses, including oxidation–reduction pathways (potentially buffering reactive oxygen species accumulation), protein folding and quality‑control mechanisms (limiting proteotoxic stress), and translational regulation (modulating protein synthesis under conditions of limited energy and water availability) (Figure S7). This module‑level organization is consistent with previous wheat network studies, which have identified early‑drought co‑expression units enriched for abscisic acid (ABA) and reactive oxygen species (ROS) signaling, osmotic adjustment, and stress‑protective machineries, particularly when network connectivity is used as a criterion for candidate prioritization [125, 126]. Within this predictive framework, *Genegroup_2122*, supported by both co‑expression and co‑citation evidence, was associated with terms related to translational elongation and metal‑ or ion‑responsive processes. During drought stress, fine‑tuning of translational activity is thought to buffer cellular stress by reducing bulk protein synthesis while preserving the production of select stress‑protective proteins. Moreover, annotations related to metal and ion responsiveness frequently intersect with redox homeostasis and ROS detoxification pathways, reflecting their roles in oxidative stress management. Collectively, these associations position *Genegroup_2122* within a putative “proteostasis–redox” regulatory axis, a network feature that has been repeatedly linked to improved stability and stress tolerance during early developmental stages in wheat and other cereals [125, 126].

### Systems-level integration of gene co-expression and protein interaction networks in drought response

Integrated analyses of gene co‑expression and protein–protein interaction (PPI) networks suggest a hierarchical, multi‑layered regulatory architecture underlying drought tolerance (Figure S8 and S11). The identification of a tightly connected co‑expression module comprising 20 genes with relatively uniform topology indicates a distributed regulatory framework, in which coordinated contributions from multiple genes, rather than dependence on a single master regulator, may enhance network robustness under stress conditions [127, 128]. Hub genes characterized by high betweenness centrality appear to integrate drought‑responsive hormonal signals, reactive oxygen species (ROS) dynamics, and osmoprotective pathways, whereas two smaller peripheral clusters are inferred to function as secondary signal amplifiers within the broader network context [129, 130]. Consistent with these observations, the PPI network exhibited a densely interconnected core with high node degree and reduced modular fragmentation, a topological feature commonly associated with stress‑responsive proteomes. This core was supported by four major functional clusters tentatively corresponding to signaling kinases, osmoprotective enzymes, hormonal crosstalk components, and ROS detoxification modules [131, 132]. Hub proteins, frequently comprising transcription factors, kinases, and redox‑associated regulators, are predicted to act as key integrative nodes, whose perturbation could disproportionately affect overall network stability and stress responsiveness [133]. Collectively, the convergence of gene co‑expression and PPI network topologies supports a systems‑level model in which central regulatory hubs coordinate transcriptional control, protein interactions, and downstream metabolic pathways during drought stress. These integrative network features provide a rational and biologically informed basis for prioritizing candidate genes and proteins for functional genomics studies, gene‑editing approaches, and marker‑assisted or genomic breeding strategies targeting drought resilience.

### Functional enrichment analyses

Functional enrichment analysis of hub proteins suggests a multilayered drought‑response program that integrates defense activation, carbohydrate metabolism, and ion/solute transport processes (Figure S14 and S15). The over‑representation of defense‑related and secondary metabolism pathways, including terpene synthases, is consistent with the proposed roles of terpenoids as chemical defense compounds and modulators of abscisic acid (ABA)–linked stress signaling under drought conditions [132, 134]. The enrichment of sucrose and broader carbohydrate metabolic pathways highlights the importance of carbon partitioning and osmoprotection during water deficit. Under drought stress, carbon flux is often redirected toward soluble sugars, which contribute to turgor maintenance, membrane stabilization, and redox balance. Such processes are thought to be coordinated by ABA‑regulated sugar transporters, including members of the SWEET family (e.g., SWEET13/15), which have been implicated in stress‑responsive carbohydrate redistribution [134–136]. In parallel, the enrichment of ion and phosphate transport pathways underscores the critical role of membrane transport systems in maintaining ionic homeostasis during drought stress. These transporters are also likely involved in coordinating stomatal regulation and cellular signaling through interactions with ABA‑ and reactive oxygen species (ROS)‑dependent pathways [137, 138]. At the network level, the concurrent enrichment of ABA/ROS signaling, carbohydrate metabolism, and transport‑related processes points to an integrated regulatory architecture rather than isolated pathway activation. This coordinated enrichment supports the interpretation that hub proteins occupy central regulatory positions, potentially acting as key integrators of metabolic, signaling, and transport functions during drought stress [131, 133]. Collectively, the observed enrichment patterns are consistent with a conceptual model in which defense and secondary metabolism provide early protective responses, carbohydrate metabolism contributes to osmotic adjustment and energy balance, and membrane transport systems preserve ion and phosphate homeostasis. These processes appear to be orchestrated through central hub proteins that integrate ABA‑ and ROS‑dependent regulatory pathways, highlighting promising targets for further functional validation and network‑guided breeding strategies [134, 137].

### Cross-layer integration of hub genes and hub proteins

The integration of hub genes and hub proteins demonstrates multi-level regulatory convergence in drought adaptation. Nodes maintaining centrality in both gene co-expression and PPI networks highlight their dual role in coordinating transcriptional programs and post-translational interactions. This overlap is biologically significant, as stress responses require synchronized gene expression and protein function to ensure rapid and efficient deployment of protective mechanisms under water deficit. Aldehyde dehydrogenase 1 (*TraesCS3A02G417200*) detoxifies reactive aldehydes generated during drought-induced ROS accumulation. ALDH genes, such as *TraeALDH7B1-5A*, are strongly induced under drought, and their overexpression enhances tolerance by reducing oxidative damage and improving water retention [139, 140]. Elongation factor Tu (*TraesCS5D02G542300*) is essential for protein synthesis; its accumulation under drought and heat stress preserves translation capacity and reduces ROS, while transgenic studies confirm improved tolerance through stabilized protein biosynthesis [141]. This underscores translational control as a critical axis of drought resilience. Double-strand break repair protein (*TraesCS2A02G528700*) highlights the importance of genome stability under stress. Drought-induced ROS can cause DNA double-strand breaks, and robust repair pathways involving *RAD51* and *LigIV* are vital for maintaining genomic integrity [142]. The convergence of these hubs across regulatory layers reflects a system-level strategy where plants safeguard core processes, oxidative detoxification, protein biosynthesis, and genome integrity, while integrating them into broader signaling and metabolic networks. This multi-layer connectivity supports targeting these candidates for functional validation, as their disruption could compromise transcriptional and proteomic resilience. Such integrative hubs also represent promising biomarkers for breeding and biotechnological interventions to enhance drought tolerance.

### Gene/protein relationships across functional gene groups

Network-level interrogation of candidate genes provides a systems view of drought tolerance, revealing coordinated relationships among distinct gene and protein groups. By integrating heterogeneous evidence into curated networks, the analysis moves beyond list-based annotation to uncover coherent biological axes relevant to water-deficit adaptation. This framework enables group-specific interpretations without assuming gene centrality or overextending unannotated nodes.

The integration of DNA replication and repair genes highlights genomic stability as a key drought-adaptation strategy in wheat, consistent with DNA-damage-response (DDR) frameworks linking genome maintenance to stress survival [143, 144]. *ETG1*, through its interaction with *MCM* proteins, supports replication fidelity and sustained cell division under drought [144, 145]. Its regulatory link with *TaMYB3R2* reflects coordination between DNA repair and transcriptional reprogramming, in line with *MYB* roles in drought and growth regulation [146, 147]. The association of *ETG1* with shoot length, together with reports connecting it to plant height, reinforces its dual role in genome stability and structural development [144, 145]. This positions *ETG1* and *MYB* hubs such as *TaMYB31/TaMYB30-B*,as promising targets for improving drought resilience [147, 148] (Figure S16).

Within the kinase-mediated signaling network, *XA21*, a non-specific serine/threonine receptor-like kinase, emerges as a central regulator of drought-associated root traits. Its interactions with *WRKY* transcription factors align with their established roles in stress signaling and root system modulation [149, 150]. In our dataset, *XA21* was linked to RNo under drought with nano-priming, consistent with database annotations connecting it to lateral root, root length, drought tolerance, transpiration rate, and chlorophyll content. This agreement between experimental and prior functional evidence positions *XA21* as a hub linking receptor-mediated kinase signaling with *WRKY*-driven transcriptional responses, coordinating both morphological and physiological adjustments to water deficit. Such coupling suggests that the *XA21–WRKY* module orchestrates adaptive root proliferation under drought, reinforcing root architecture as a key determinant of drought resilience. Accordingly, *XA21* represents a strong candidate for functional validation and breeding efforts targeting improved stress-adaptive root traits (Fig. 5).

In the ubiquitin-mediated protein degradation network, *SAY1* emerges as a key node interacting with the transcription factor *TaMYBR1*, which is implicated in protein turnover and stress regulation [151]. The ubiquitin–proteasome system (UPS) is central to maintaining cellular homeostasis under drought by selectively degrading proteins that limit stress adaptation [115, 152]. In our dataset, *SAY1* was associated with shoot length under nano-priming, and annotation links it to plant height, indicating a developmental role. Its connections to both morphological traits (tiller number, plant height) and physiological traits (protein and grain protein content) highlight an integrative regulatory function. Together, these patterns suggest that *SAY1* regulates growth and nutrient-related traits through ubiquitin-mediated proteostasis, coordinating balancing growth and stress responses under varying environmental conditions. This position *SAY1* is a promising candidate for functional validation and breeding strategies targeting drought-resilient wheat (Fig. 6).

The ion-transporter network highlights coordinated vacuolar/tonoplast regulation of water and ion homeostasis under drought. *CAX1b*, a vacuolar Ca^2^⁺/H⁺ exchanger ortholog, aligns with evidence that *CAX1*-type transporters modulate Ca^2^⁺ signaling and redox balance during stress, thereby adjusting downstream responses [153]. Tonoplast aquaporins such as *TIP1*;*2/TIP* family members contribute to vacuolar water flux and solute exchange, and their modulation is repeatedly linked to improved dehydration tolerance and ROS balance in cereals [154]. Within this network, *PHIP1* introduces an RNA-centric regulatory layer: *PHIP1* proteins contain CCHC Zn-finger and RNA-binding motifs, localize to cytokinetic structures, and can couple ion-transport states to post-transcriptional regulation under stress. Its detection specifically under drought with ZnO-NPs priming supports a role in Zn-linked modulation of stress signaling and RNA–protein interactions [155]. Regulatory connections between *PHIP1/CAX1b* and transcription factors such as *MYB3R2* and *NACs* correspond to the well-established roles of *MYB* and *NAC* modules in abiotic stress programs and root development in wheat [156, 157] (Fig. 7).

Transcription factors are central regulators of drought adaptation. *HSFA2* enhances tolerance by activating chaperone and antioxidant pathways [158, 159], and its interaction with *DREB2* reflects activation of the ABA-independent drought pathway critical for osmotic adjustment [160, 161]. Likewise, *NAC* factors, including *NAC044*, act as stress hubs that trigger cell-cycle arrest and redirect resources toward survival, balancing growth with stress responses [162, 163] (Figure S17).The second regulatory module highlights transcription factors connecting RNA processing with reproductive development. *MYB80*, essential for tapetal PCD and pollen viability, is conserved across crops, indicating a broader stress-related role [164, 165]. Integrating RNA metabolism with stress signaling enables plants to adjust developmental timing during drought, consistent with ABA-independent drought-escape and photoperiod-regulated flowering pathways [166, 167] (Fig. 8). These combined networks position *HSFA2*, *DREB2*, *NAC044*, and *MYB80* as strong candidates for functional validation and breeding for drought-resilient performance.

A J-domain co-chaperone working with *HSP70* highlights proteostasis as an early drought-response mechanism, consistent with stress-responsive roles of plant J‑domain proteins (JDPs) in folding and cell-division interfaces [168]. Its regulatory connection to a *MADS*‑box factor (*MADS51*) aligns with evidence that *MADS* proteins fine-tune growth–stress trade-offs, with *SiMADS51* acting as a negative regulator of drought tolerance [169]. The gene’s association with metal-binding proteins and its detection specifically under ZnO-NP priming suggest coupling between chaperone capacity and zinc-mediated signaling. ZnO priming is known to enhance antioxidant defenses, osmoprotection, and seedling vigor under drought in cereals, supporting a plausible Zn‑triggered priming layer that integrates with proteostasis and transcriptional control [51, 170, 171]. Reviews emphasize zinc’s central role in ROS detoxification, aquaporin function, and Zn-finger transcription networks, offering a mechanistic bridge between metal homeostasis and adaptive growth [172, 173]. Together, these findings support a model in which *JDP/HSP70* proteostasis, *MADS51*-like regulation, and Zn-triggered priming cooperate to stabilize growth under water deficit (Figure S18).

*TaTPS14* involvement in terpene biosynthesis highlights the contribution of secondary metabolites to drought adaptation. Terpenoids modulate hormone signaling and membrane stability, influencing root and shoot architecture under water deficit [174]. Regulatory links between *TaTPS14*, *TaNAC011*, and multiple *WRKY*/*MYB* transcription factors indicate integration of terpenoid pathways with stress-responsive transcriptional networks. *WRKYs* act as molecular switches for osmoprotection and antioxidant defense [175], while *MYBs* coordinate secondary metabolism and stress signaling. Both *TaTPS14* genes were associated with shoot length after nano priming, suggesting that enhanced terpene pathway activity may underline priming-induced growth and drought readiness. Overall, this network connects terpene biosynthesis, hormonal signaling, and TF regulation to support growth and stress resilience, making *TaTPS14*, *NAC*, *WRKY*, and *MYB* components strong candidates for functional validation (Figure S19).

This metabolism subnetwork highlights a drought-responsive hormone–energy hub. *CPR/P450s* regulate stress-induced secondary and hormone metabolism [176], while tryptophan synthase supports auxin production essential for root adaptation [174]. Together, these subnetworks describe two complementary drought strategies: redox/structural protection and hormone-driven metabolic reallocation, underpinning improved root–shoot growth under drought and nano-priming (Fig. 9). Subnetwork II highlights a redox–phenylpropanoid defense module. *HIPL2* likely contributes to NADPH-dependent ROS detoxification, consistent with the established roles of Glucose-6-Phosphate Dehydrogenase (*G6PDH*) and other Pentose Phosphate Pathway (*PPP*) enzymes in maintaining redox balance under drought [177]. In parallel, *REF1* mediates oxidation of coniferaldehyde/sinapaldehyde to sinapic and ferulic acids, reinforcing cell walls and reducing water loss, functions essential for drought-related structural stability [178]. Their regulatory association with *MYB* and *MYC2* transcription factors aligns with known drought-signaling networks coordinating ABA/JA responses, root development, and reproductive resilience [147, 179–181] (Figure S20).

## Conclusion

This study provides comprehensive insights into the genetic architecture and molecular mechanisms underlying drought tolerance during wheat germination and early seedling growth. By integrating QTL mapping with gene–gene and protein–protein interaction analyses, we identified 51 QTLs associated with 22 germination and seedling traits under contrasting water conditions, with and without ZnO nanoparticle priming. Functional annotation revealed candidate genes encoding transcription factors, ion transporters, enzymes, and stress-related proteins, many of which showed differential expressions under drought stress. Network analyses highlighted hub genes and proteins involved in signaling, proteostasis, and metabolic adjustment, underscoring their central role in drought adaptation. ZnO nano-priming consistently improved germination performance and mitigated drought-induced reductions, suggesting its potential as a practical strategy for enhancing early vigor and resilience. These findings offer valuable targets for molecular breeding and integrative approaches combining nanotechnology and genomics to develop drought-tolerant wheat cultivars for sustainable agriculture under climate change. However, given the relatively small population, the identified loci should be considered as candidate regions and require further validation in larger and independent populations before their application in breeding programs.

## Supplementary Information

Supplementary Material 1.

Supplementary Material 2.

## Data Availability

The datasets generated and/or analyzed during the current study are included in this published article and its supplementary information files. Additional data supporting the findings of this study are available from the corresponding author upon reasonable request.
