# Supplementary material for: Deciphering the genetic basis of wheat germination under ZnO nano priming and drought stress through integrated QTL mapping and network analyses
Source: BMC Plant Biol. 2026 May 11;26:831. doi: 10.1186/s12870-026-08898-9 (PMC13162379; doi:10.1186/s12870-026-08898-9)
Supplement: Supplementary file 1 — Supplementary Material 1. [file 12870_2026_8898_MOESM1_ESM.pdf]

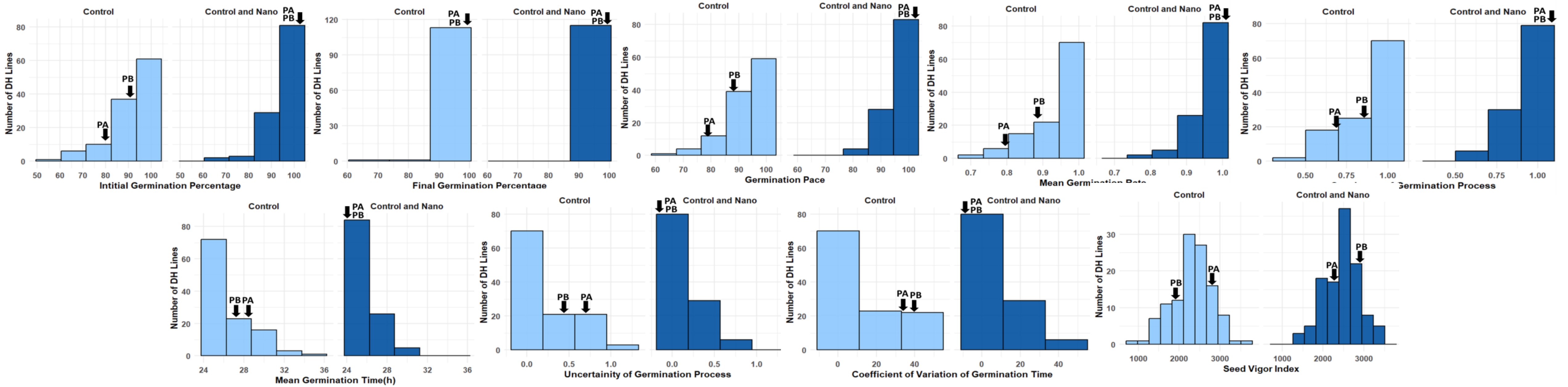

a) Germination-related traits

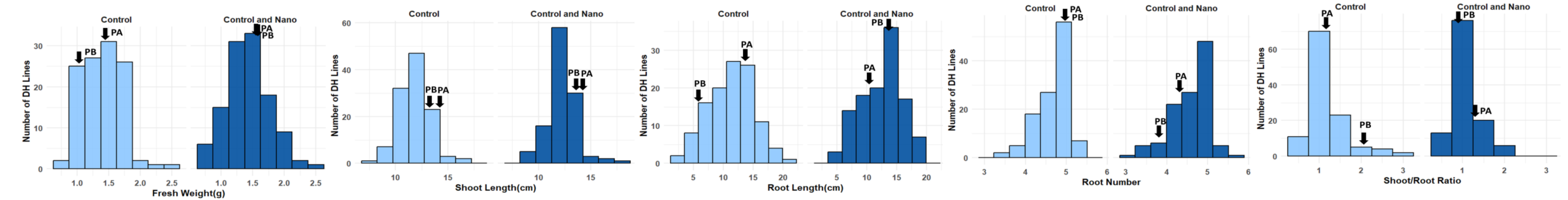

b) Seedling-establishment traits

Figure S1: Frequency distribution of germination and seedling parameters under control with and without nano priming; a) Germination-related traits and b) Seedling-establishment traits. PA= TRI 10703, PB= TRI-5310, the sky-blue color refers to control conditions with unprimed, and the deep blue color refers to control conditions with nano priming.

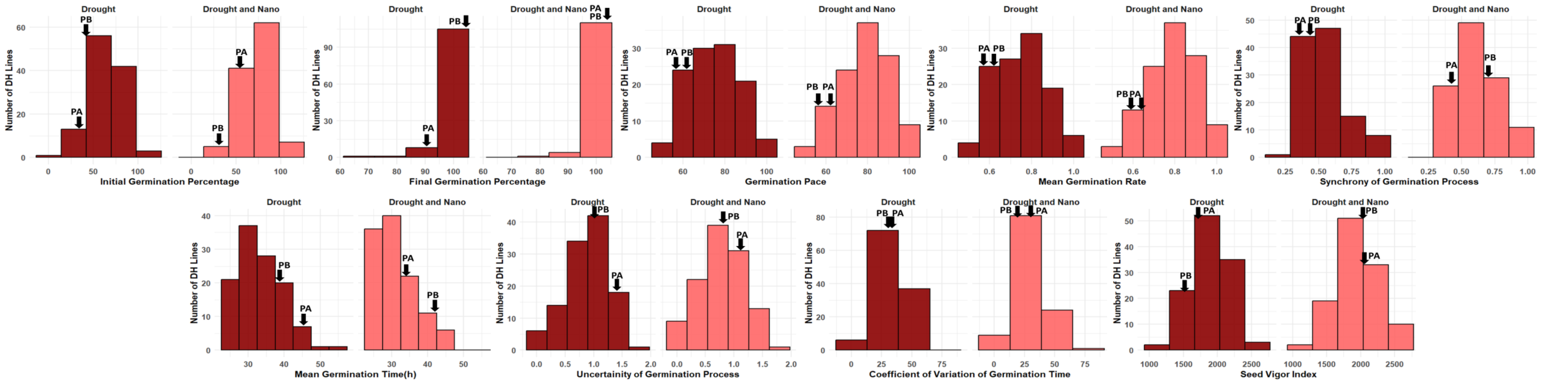

a) Germination-related traits

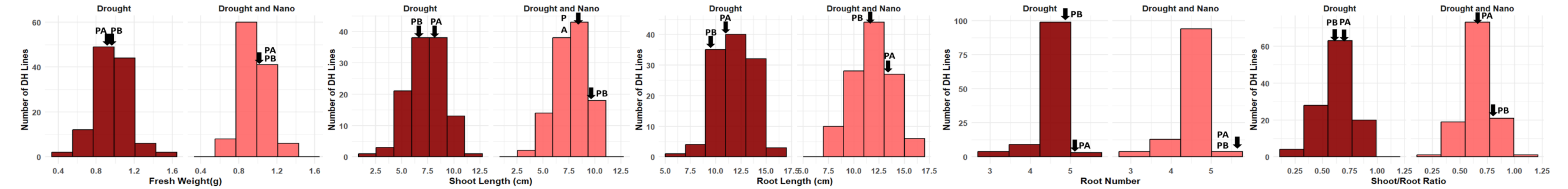

b) Seedling-establishment traits

Figure S2: Frequency distribution of germination and seedling parameters under drought with and without nano priming; a) Germination-related traits and b) Seedling-establishment traits. PA= TRI 10703, PB= TRI-5310, the deep red color refers to drought conditions with unprimed, and the light red color refers to drought conditions with nano priming.

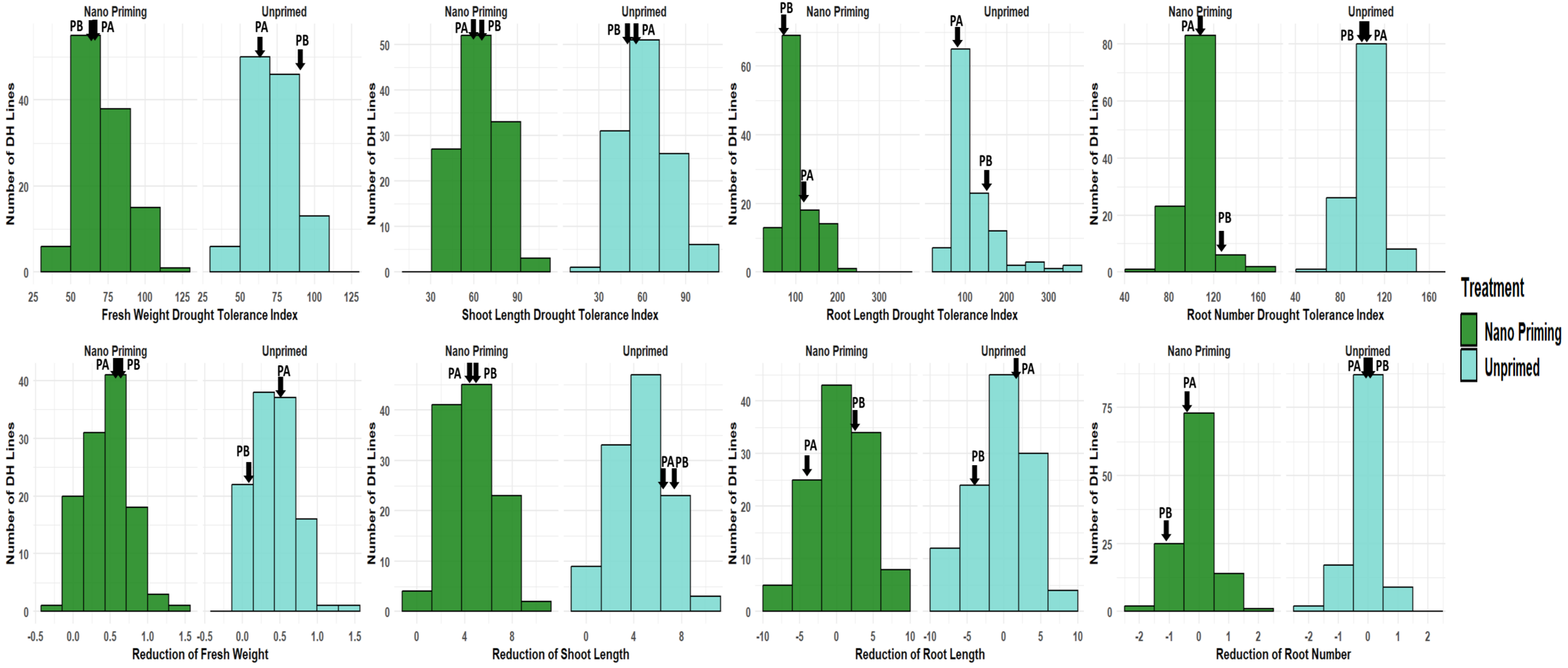

Figure S3: Frequency distribution of drought tolerance index related to seedling parameters under unprimed and nano priming. PA= TRI 10703, PB= TRI-5310, the pale green color refers to drought tolerance indexes with unprimed, and the forest green color refers to control conditions with nano priming

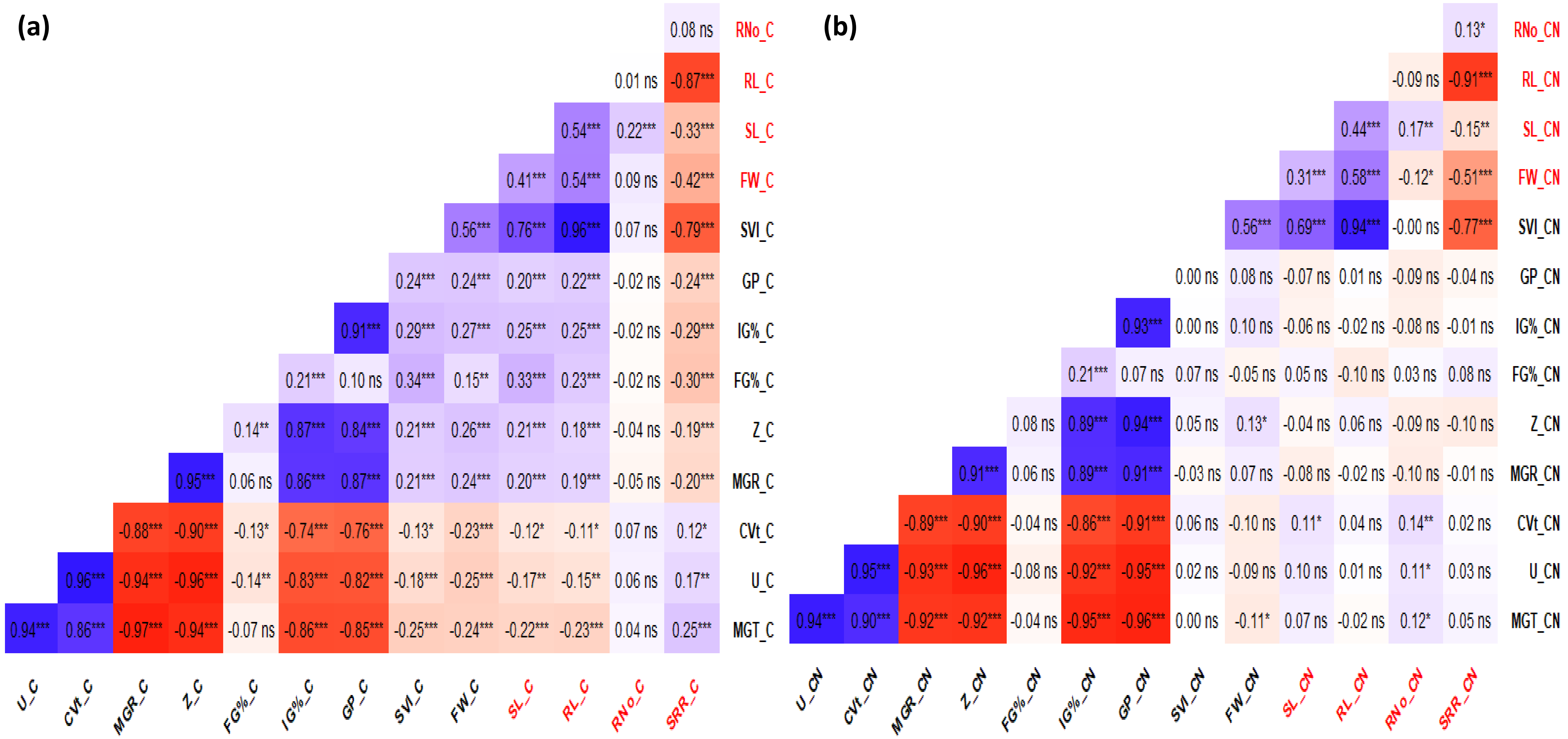

Figure S4: Phenotypic correlation matrices of germination and seedling-related traits under control conditions (a) without nano-priming and (b) with nano-priming. Trait names for seedling-related traits are shown in red, whereas germination-related traits are shown in black. The intensity and color direction represent the strength and sign of Pearson's correlation coefficients, ranging from –1 (strong negative, red) to +1 (strong positive, blue), as indicated by the accompanying scale bar. Full traits descriptions are provided in Table 1.

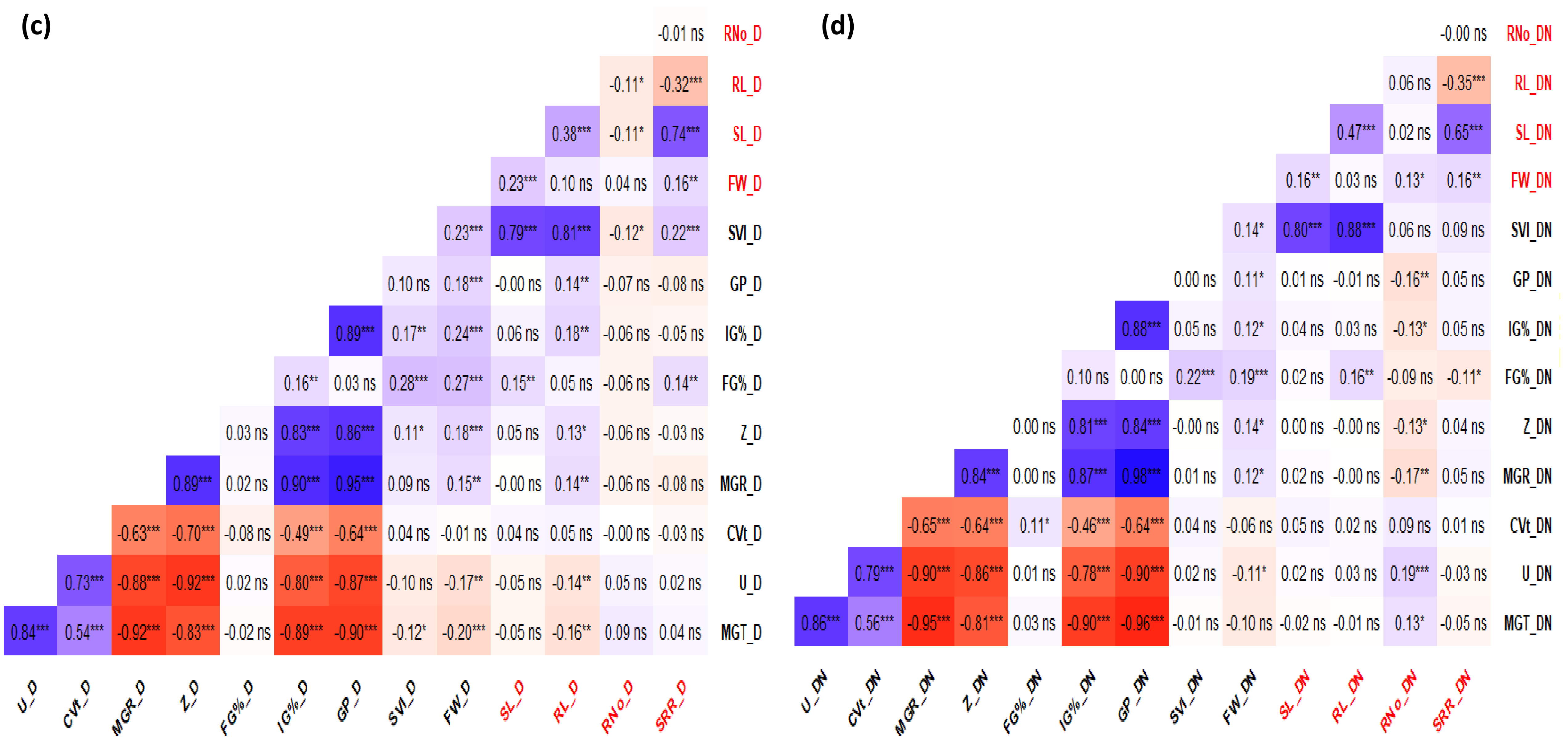

Figure S5: Phenotypic correlation matrices of germination and seedling-related traits under droght conditions (a) without nano-priming and (b) with nano-priming. Trait names for seedling-related traits are shown in red, whereas germination-related traits are shown in black. The intensity and color direction represent the strength and sign of Pearson's correlation coefficients, ranging from – 1 (strong negative, red) to + 1 (strong positive, blue), as indicated by the accompanying scale bar. Full traits descriptions are provided in Table 1.

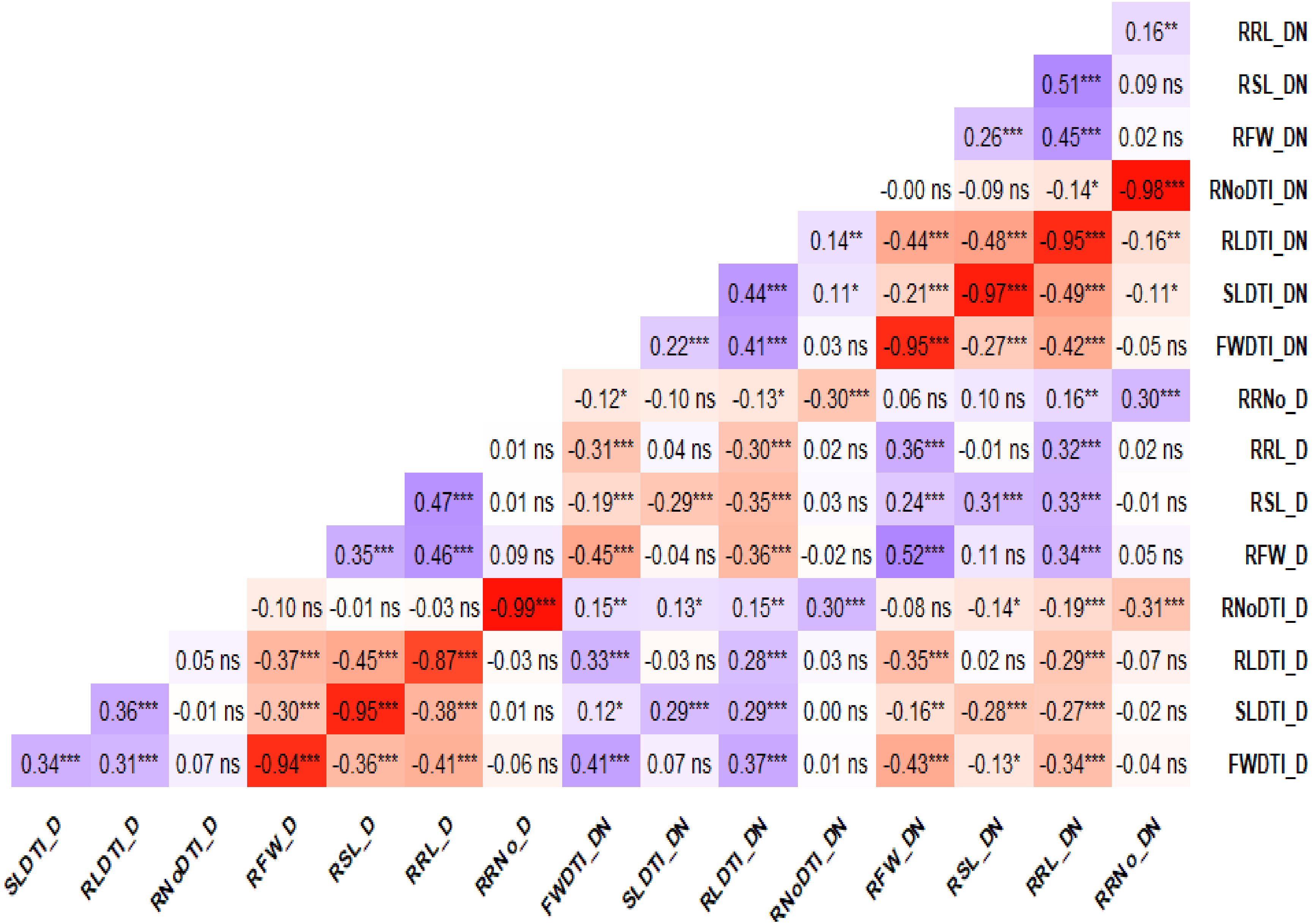

Figure S6: Phenotypic correlation matrices of drought tolerance index and reduction-related traits scored under unprimed and nano priming. The intensity and color direction represent the strength and sign of Pearson's correlation coefficients, ranging from – 1 (strong negative, red) to + 1 (strong positive, blue), as indicated by the accompanying scale bar. Full traits descriptions are provided in Table 1.

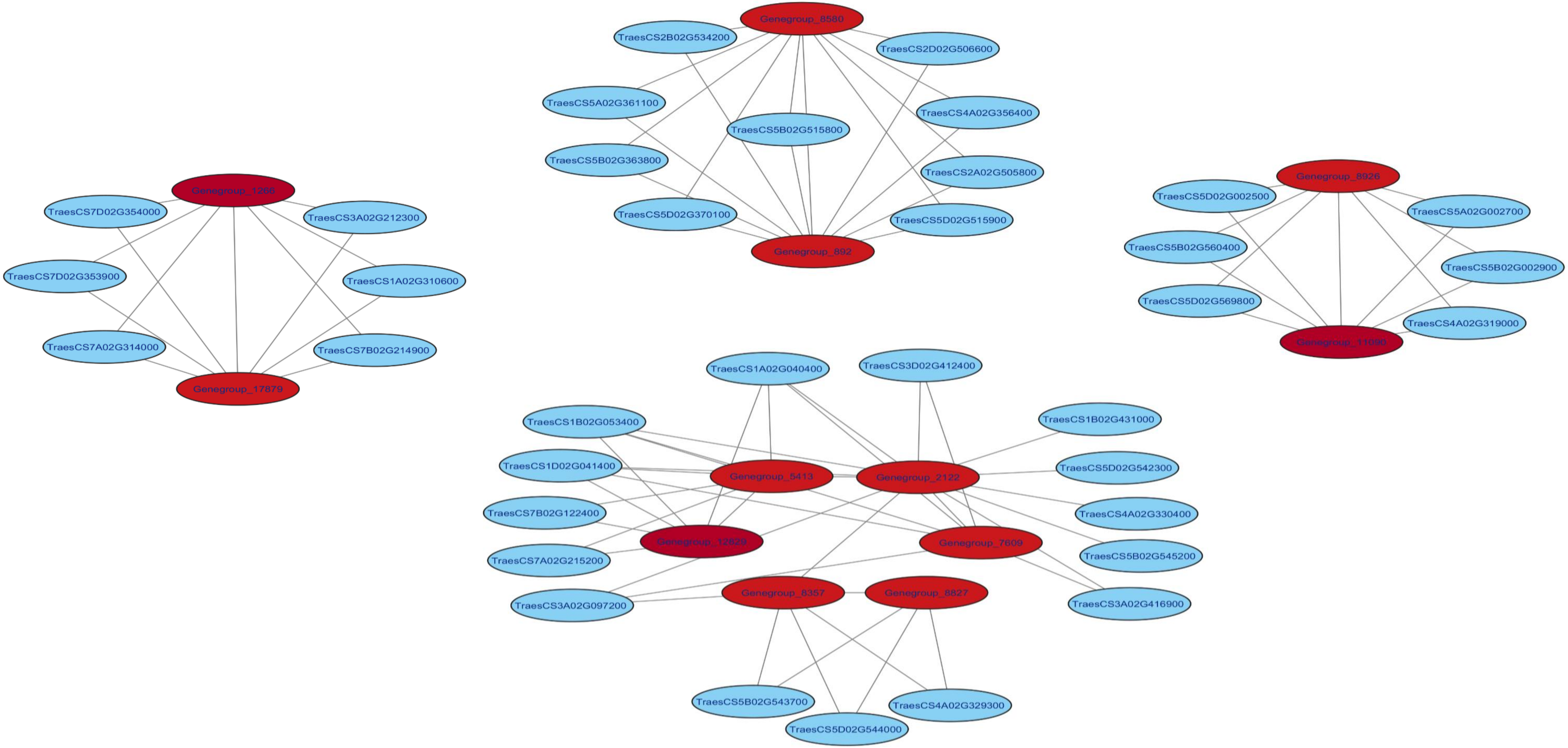

Figure S7: Network-based identification and prioritization of drought-related wheat candidate genes using WheatNet guided by QTL-derived genes under drought-related conditions.

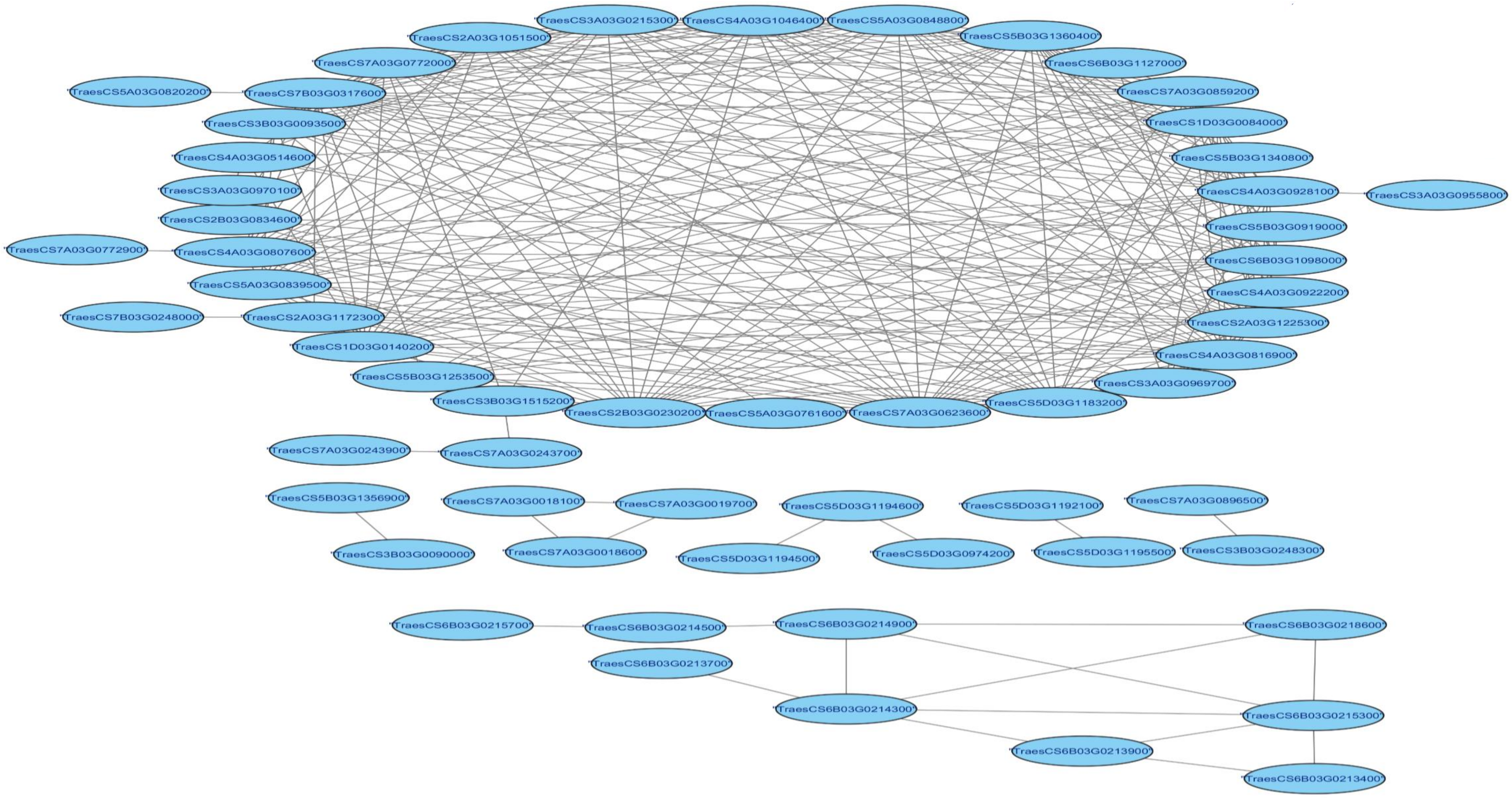

Figure S8: Co-expression network of prioritized wheat candidate genes based on WGRN correlation scores (threshold ≥ 0.6), showing nodes as genes and edges as significant co-expression links

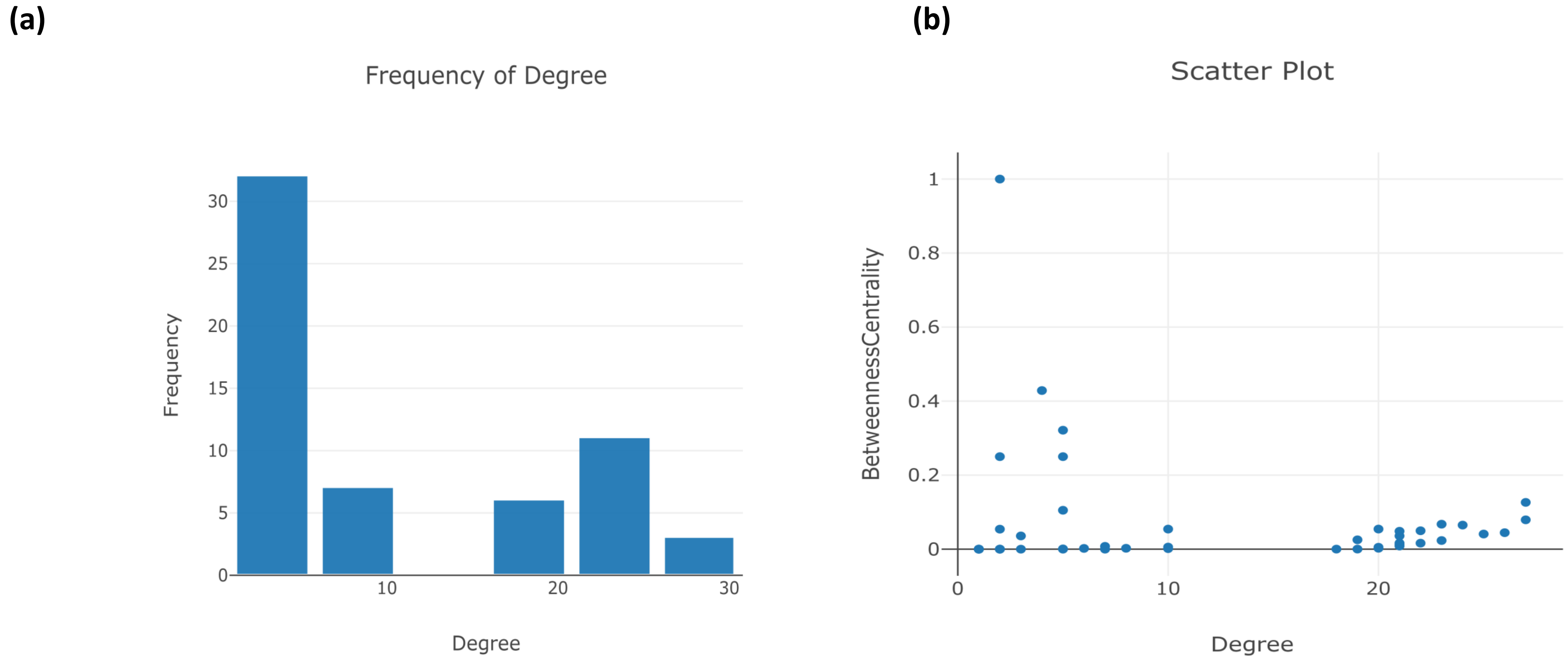

Figure S9: Node connectivity and centrality metrics of wheat candidate genes visualized through (a)degree distribution (histogram) and (b) degree–centrality relationships (scatter plot)

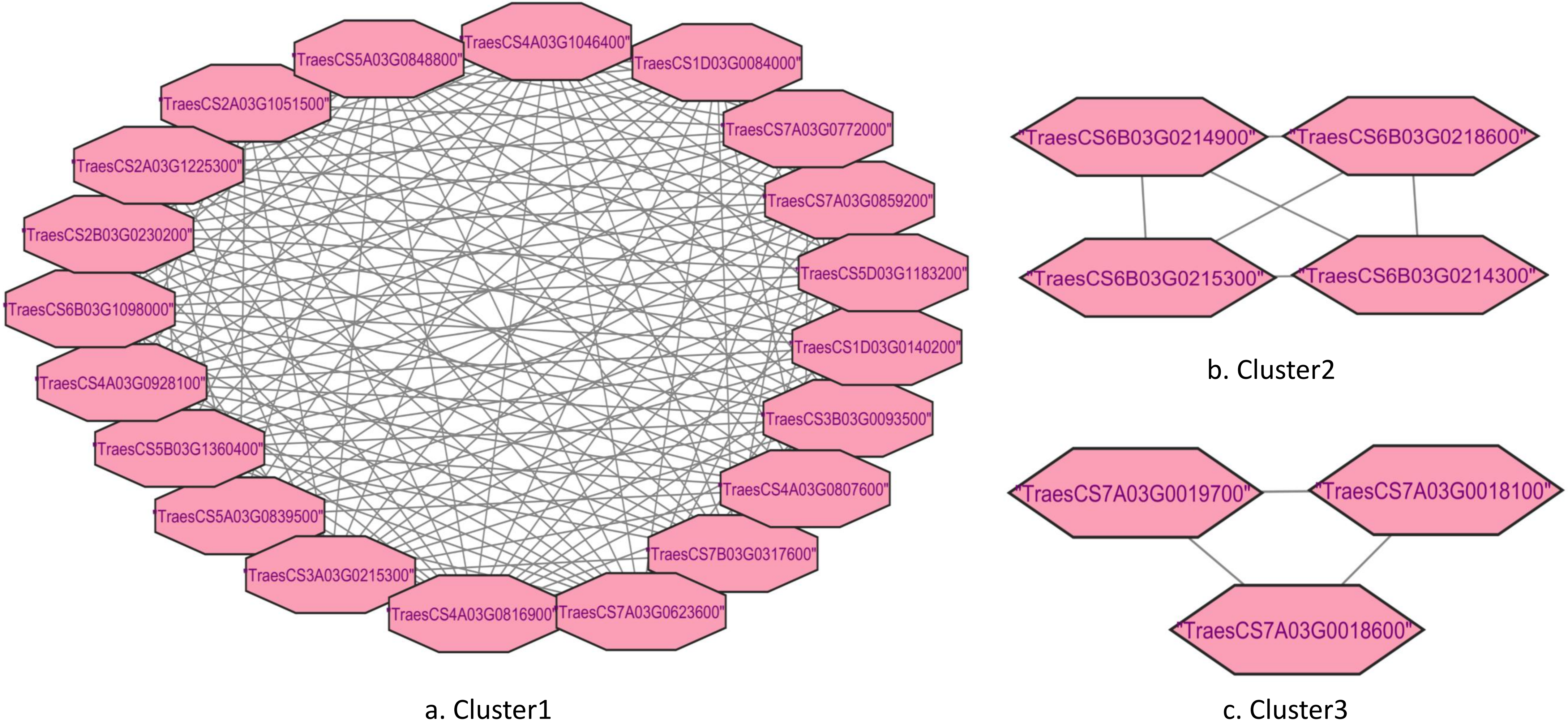

Figure S10: Co-expression network of prioritized wheat candidate genes based on WGRN correlation scores (threshold ≥ 0.6), showing gene nodes, co-expression edges, and the resulting gene clusters

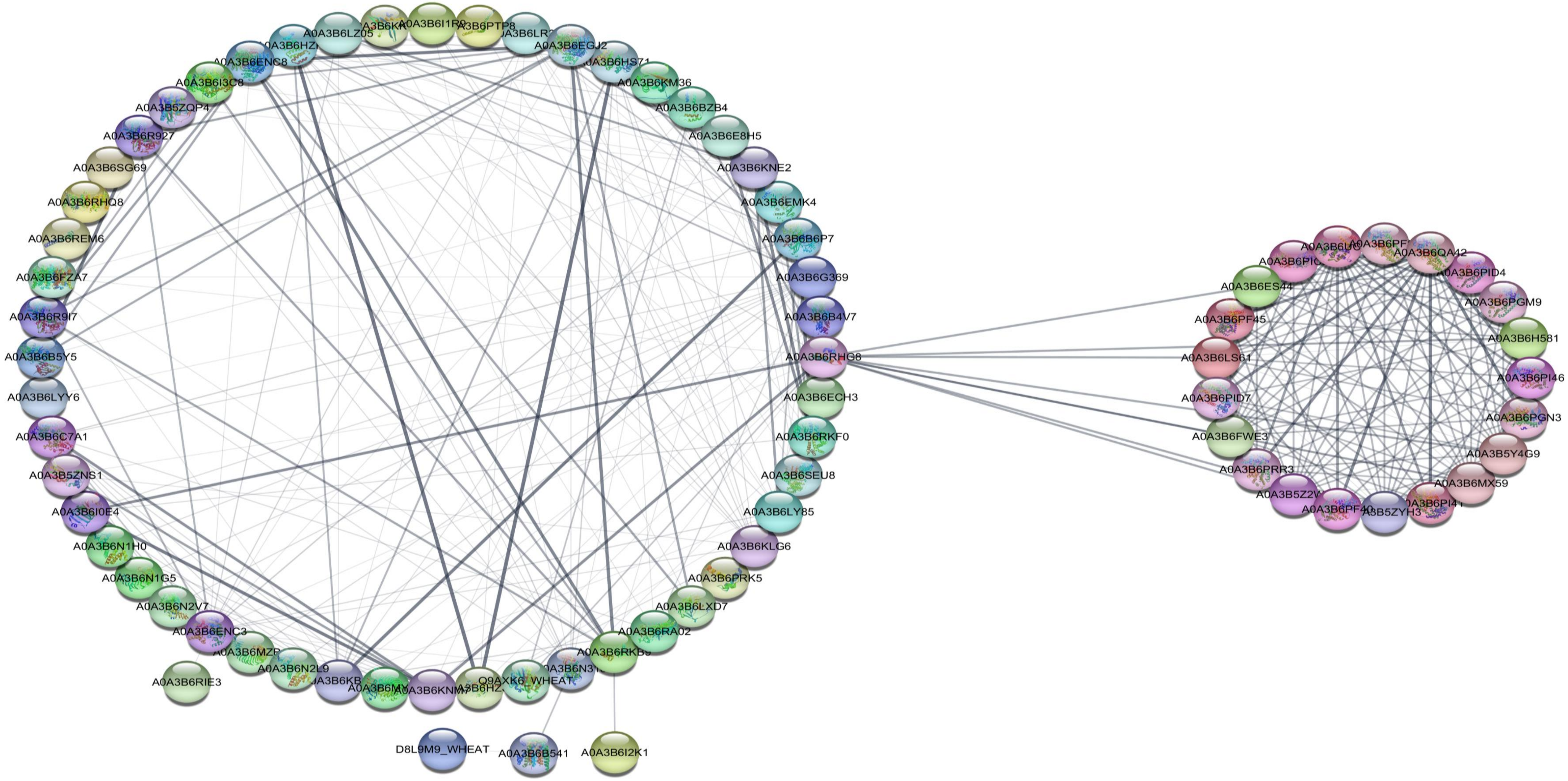

Figure S11:High-confidence Protein-Protein Interaction PPI network constructed using STRING, where nodes represent the protein products of wheat candidate genes and edges represent their interaction links.

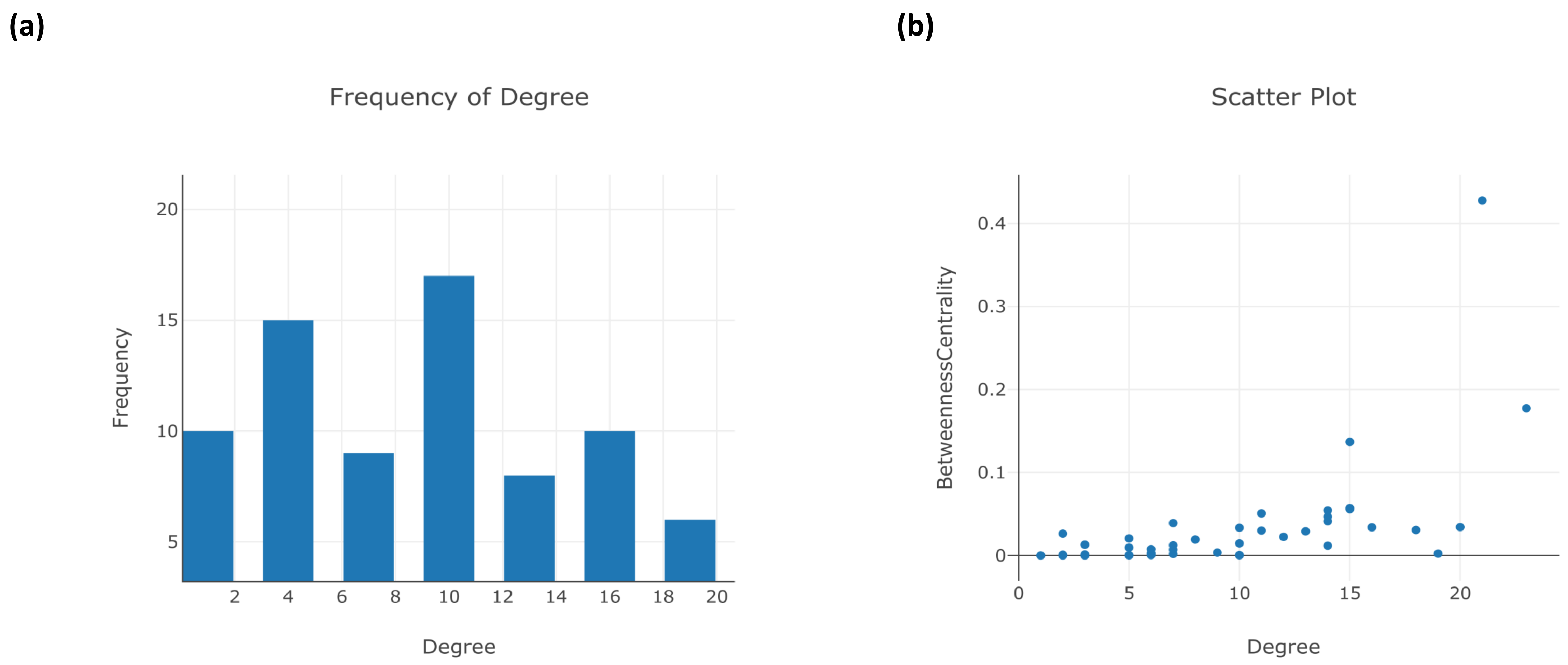

Figure S12: Node connectivity and centrality metrics of wheat candidate proteins visualized through (a) degree distribution (histogram) and (b)degree–centrality relationships (scatter plot).

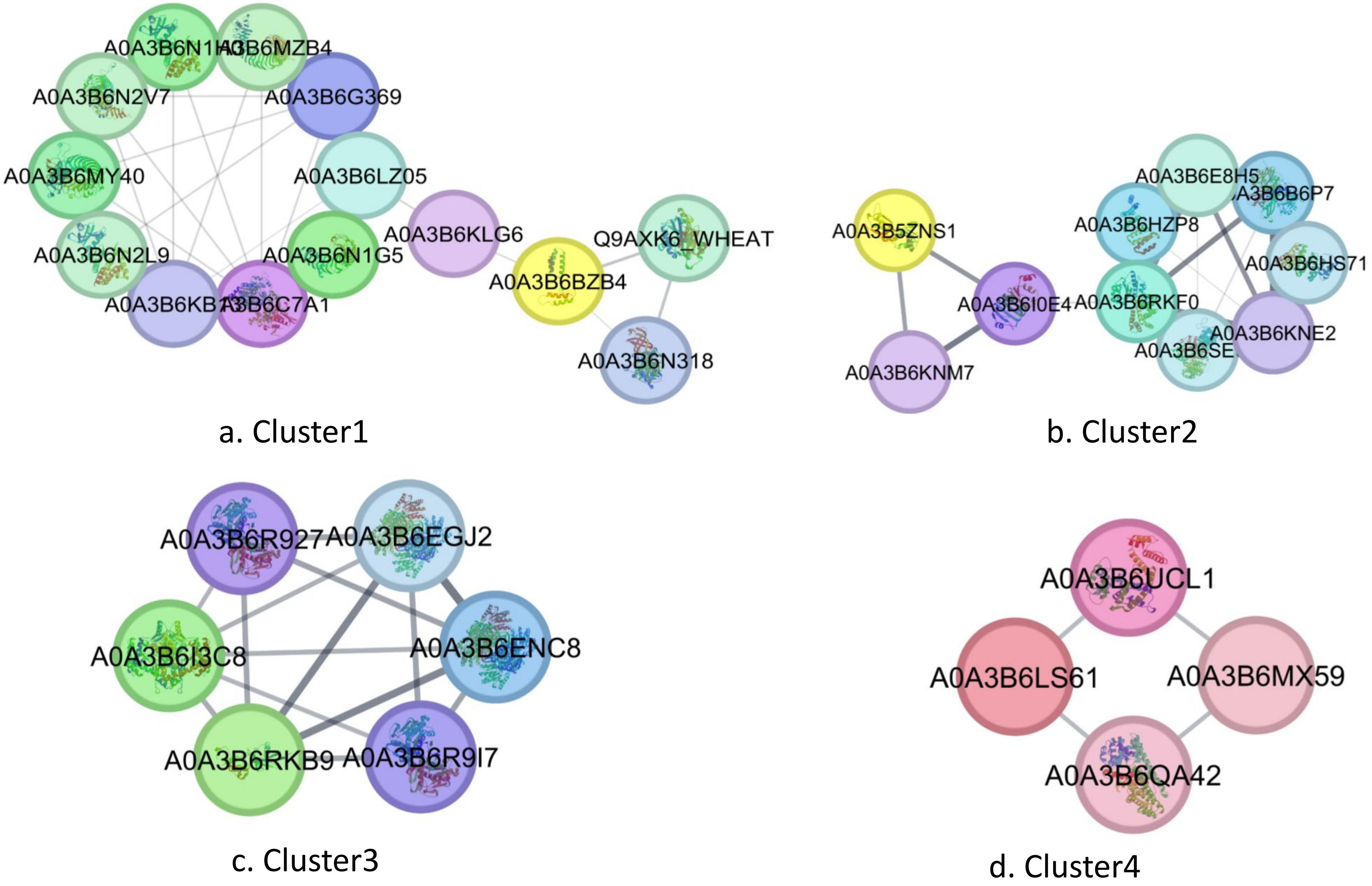

Figure S13: High-confidence PPI network constructed using STRING, where nodes represent the protein products of wheat candidate genes and edges represent their interaction links, highlighting the resulting protein interaction clusters

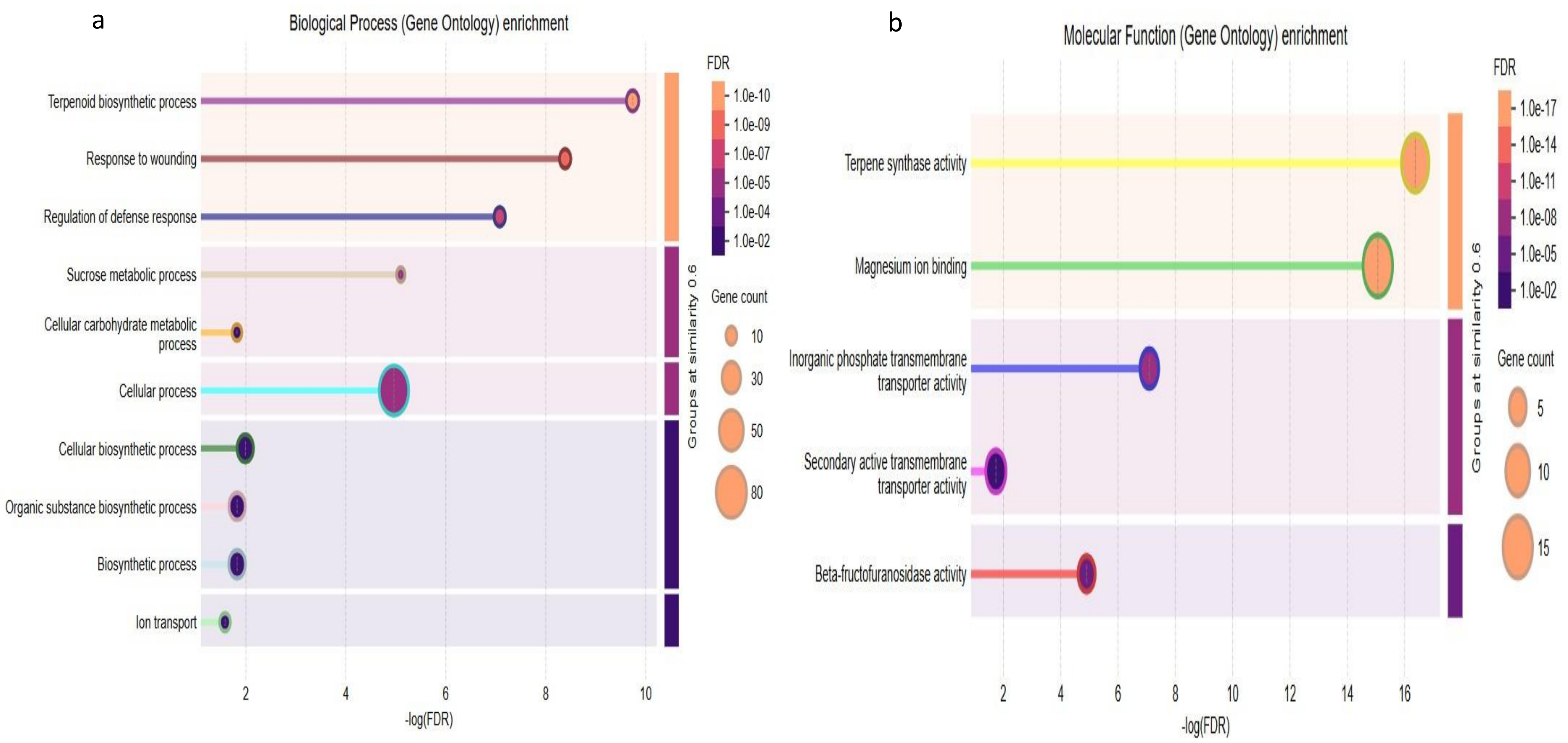

Figure S14: Functional Enrichment Analyses of biological process and molecular function.

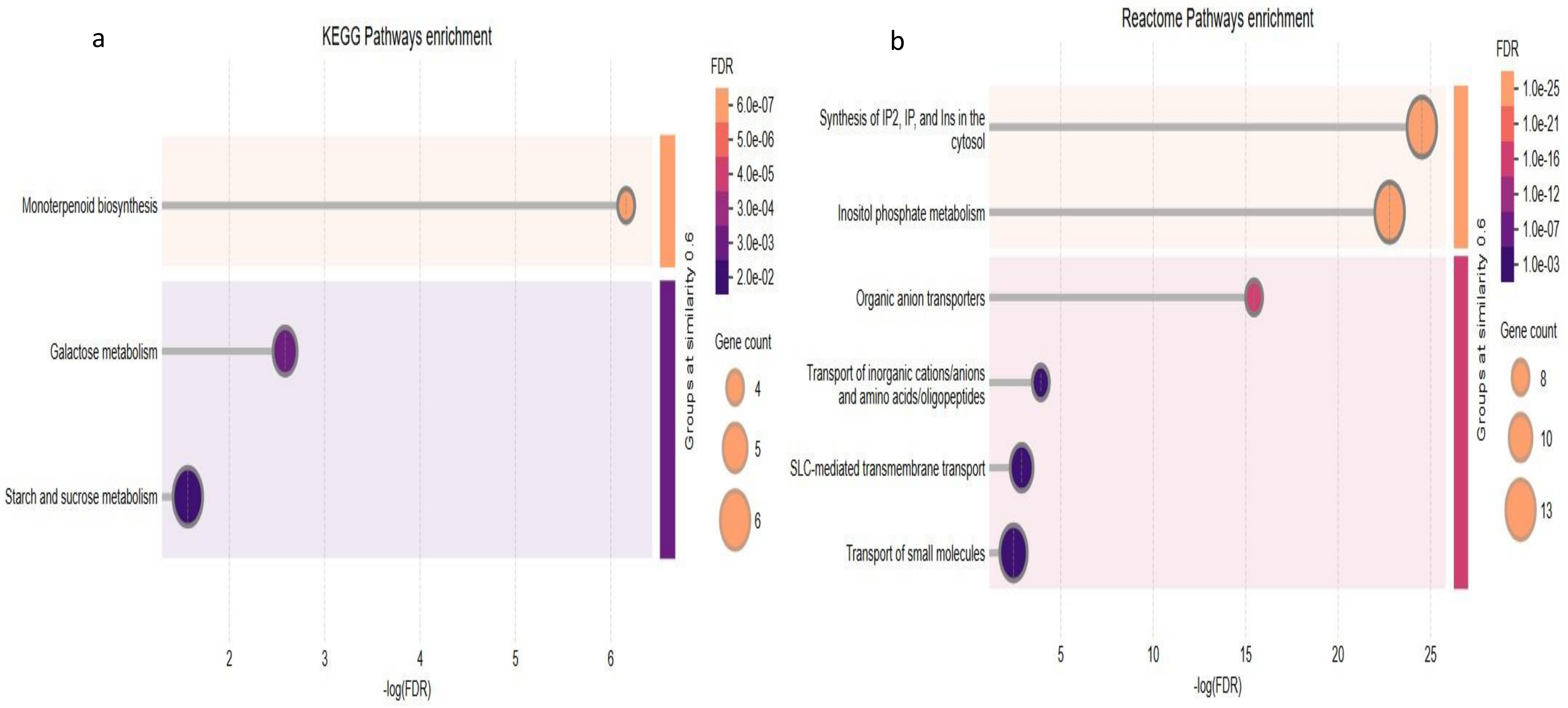

Figure S15: Functional Enrichment Analyses of KEGG and reactome pathways.

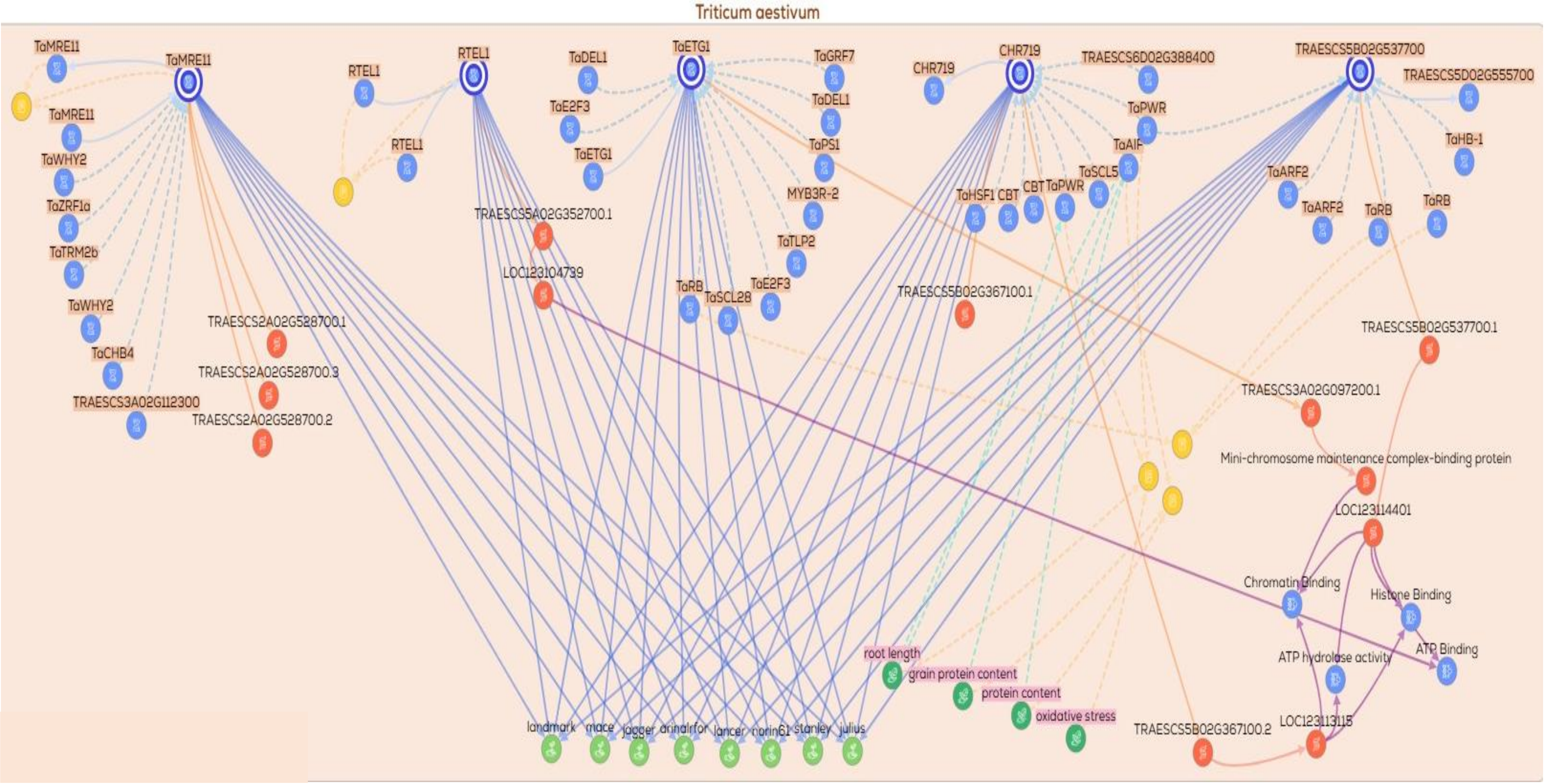

Figure S16: Gene/Protein Interaction Network of DNA Replication / Repair related genes.

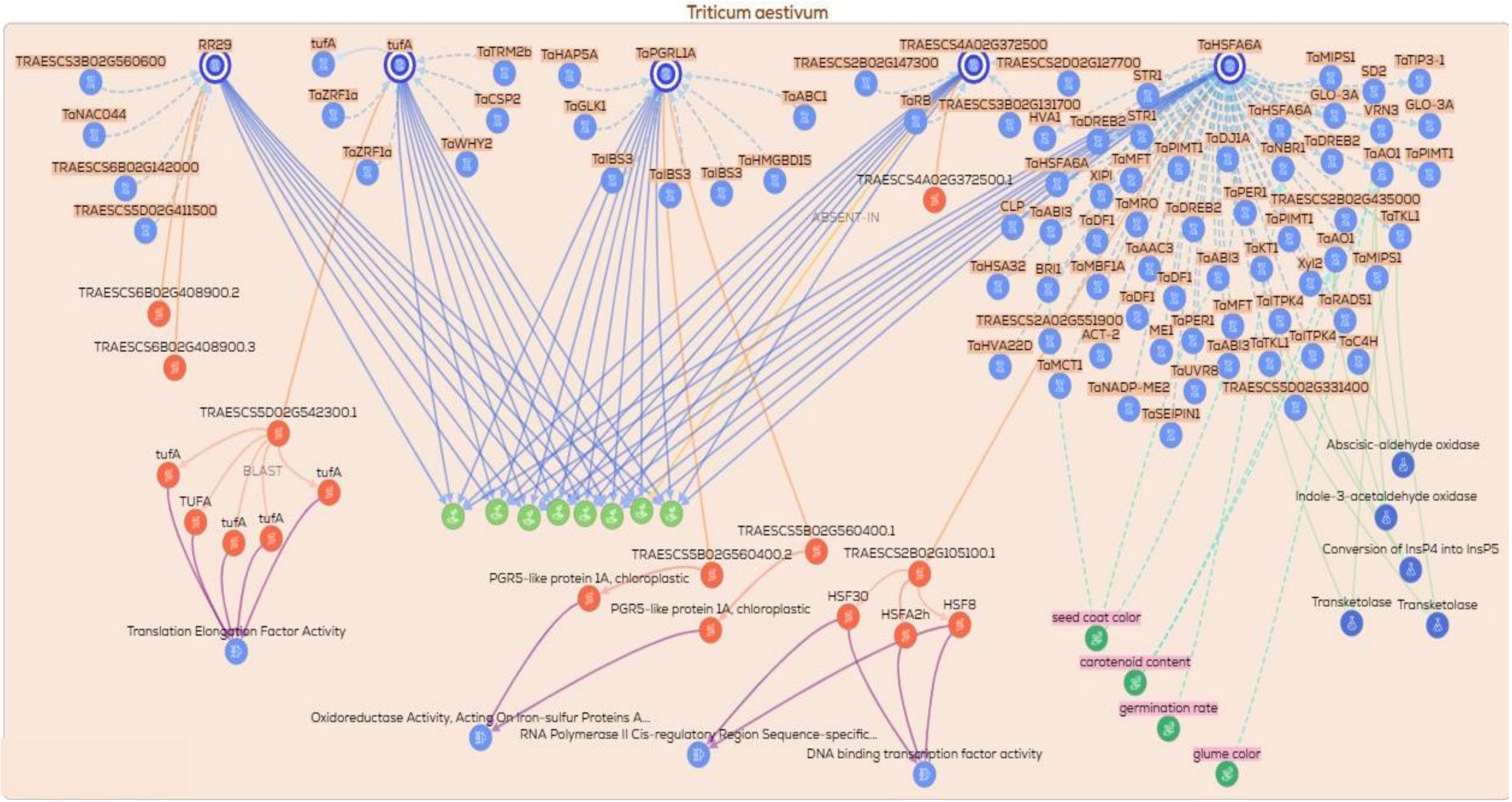

Figure S17:Gene/Protein Interaction Network of transcription factors linked to translation, protein synthesis, photosynthesis, and chloroplast-related genes.

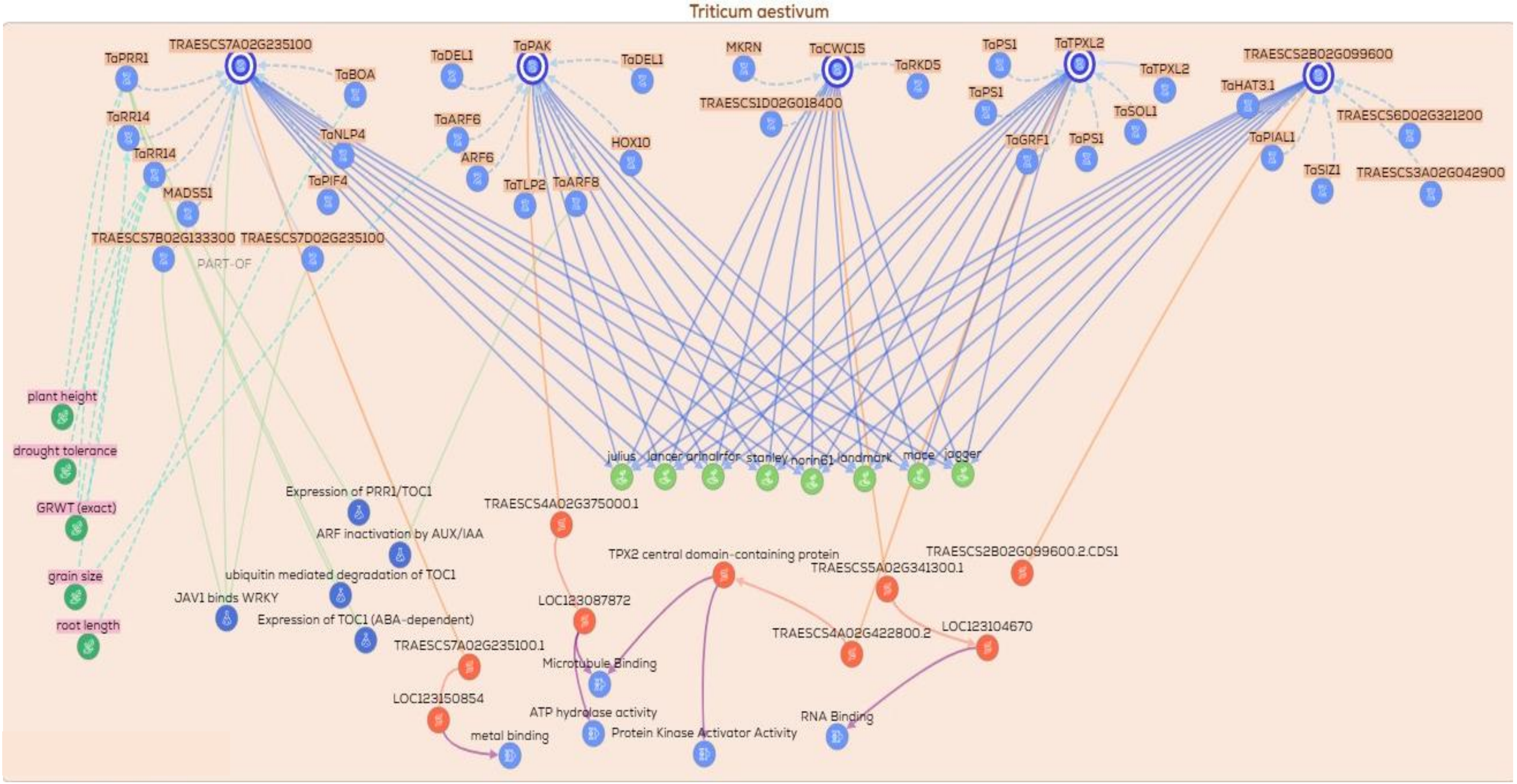

Figure S18:Gene/Protein Interaction Network of protein folding and chaperone–cytoskeleton/cell division network related genes.

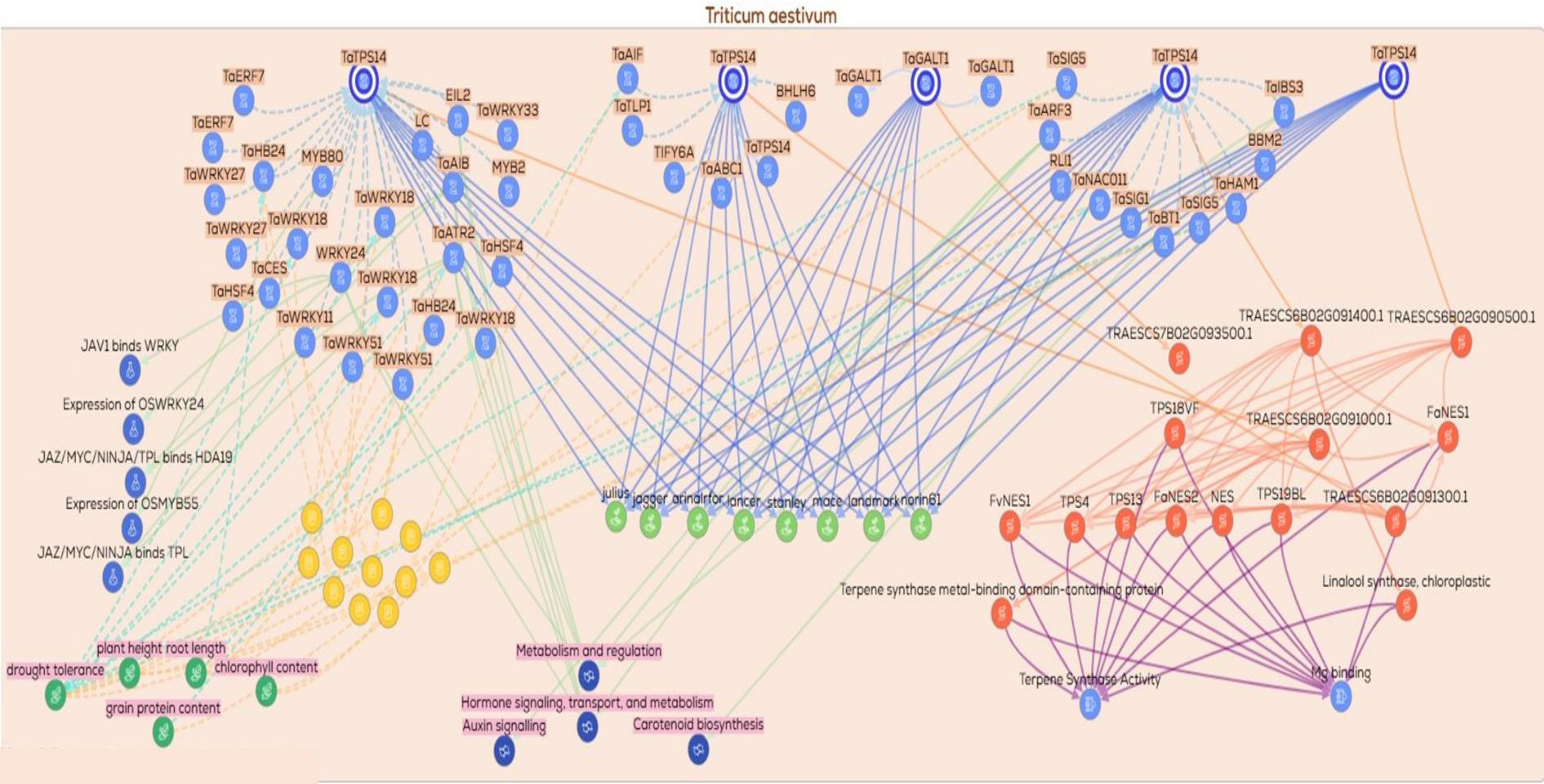

Figure S19:Gene/Protein Interaction Network of Secondary Metabolism/Specialized related genes.

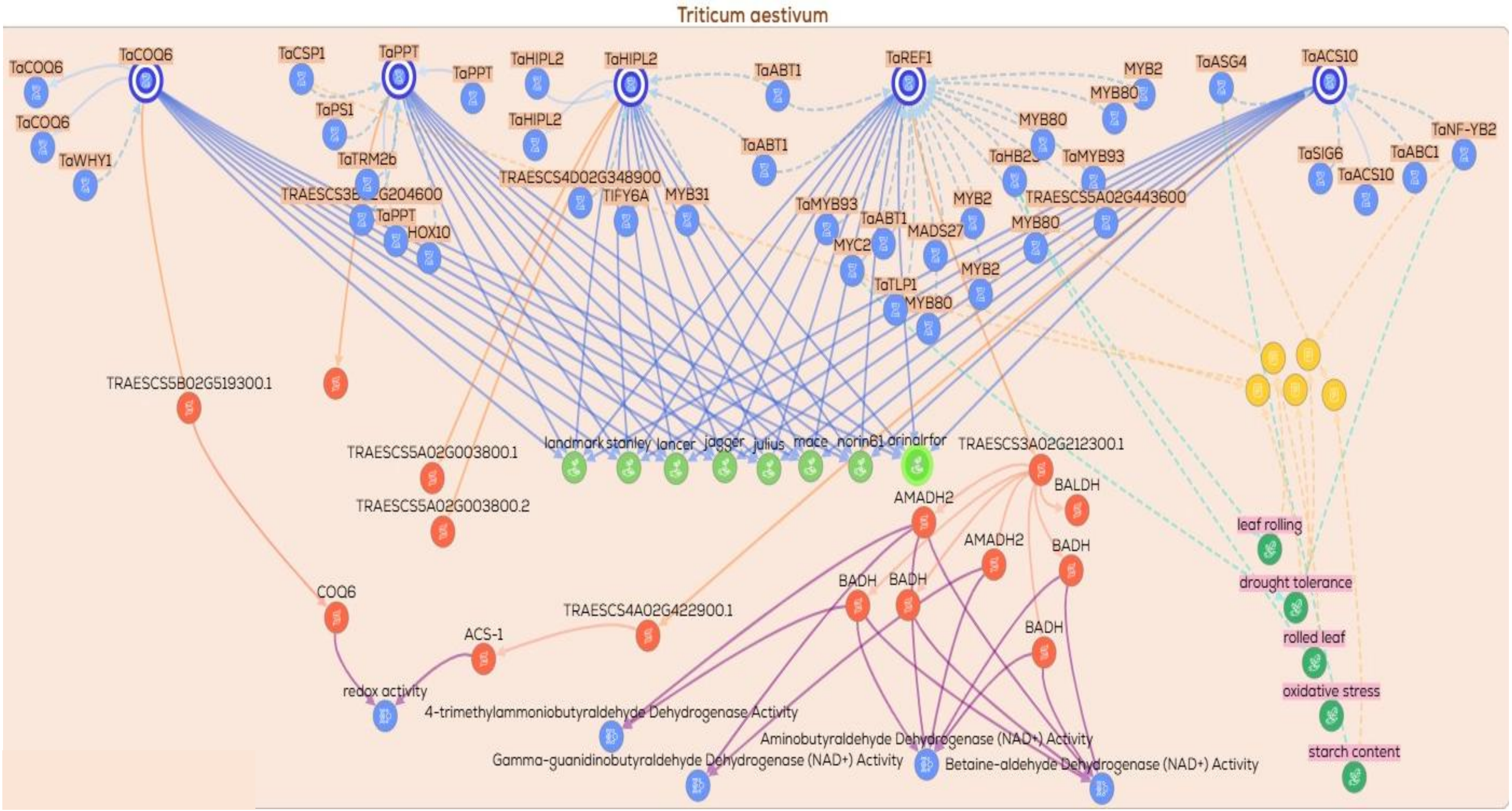

Figure S20:Gene/Protein Interaction Network 2 of Enzymes/Metabolism related genes.
